# Probing the Inhibition of Microtubule Affinity Regulating Kinase 4 by *N*-Substituted Acridones

**DOI:** 10.1038/s41598-018-38217-8

**Published:** 2019-02-08

**Authors:** Maria Voura, Parvez Khan, Savvas Thysiadis, Sotiris Katsamakas, Aarfa Queen, Gulam Mustafa Hasan, Sher Ali, Vasiliki Sarli, Md. Imtaiyaz Hassan

**Affiliations:** 1 Department of Chemistry, Aristotle University of Thessaloniki, University Campus, 54124 Thessaloniki, Greece; 2 Centre for interdisciplinary research in Basic Sciences, Jamia Millia Islamia, Jamia Nagar, New Delhi 110025 India; 3 Department of Pharmaceutical Chemistry, School of Pharmacy, Aristotle University of Thessaloniki, University Campus, 54124 Thessaloniki, Greece; 4 Department of Chemistry, Jamia Millia Islamia, Jamia Nagar, New Delhi 110025 India; 5 Department of Biochemistry, College of Medicine, Prince Sattam Bin Abdulaziz University, P.O. Box 173, Al-Kharj, 11942 Saudi Arabia

## Abstract

Microtubule affinity regulating kinase 4 (MARK4) becomes a unique anti-cancer drug target as its overexpression is responsible for different types of cancers. In quest of novel, effective MARK4 inhibitors, some acridone derivatives were synthesized, characterized and evaluated for inhibitory activity against human MARK4. Among all the synthesized compounds, three (**7b, 7d** and **7f**) were found to have better binding affinity and enzyme inhibition activity in µM range as shown by fluorescence binding, ITC and kinase assays. Here we used functional assays of selected potential lead molecules with commercially available panel of 26 kinases of same family. A distinctive kinase selectivity profile was observed for each compound. The selective compounds were identified with submicromolar cellular activity against MARK4. Furthermore, *in vitro* antitumor evaluation against cancerous cells (MCF-7 and HepG2) revealed that compounds **7b, 7d** and **7f** inhibit cell proliferation and predominantly induce apoptosis in MCF-7 cells, with IC_50_ values of 5.2 ± 1.2 μM, 6.3 ± 1.2 μM, and 5.8 ± 1.4 μM respectively. In addition, these compounds significantly upsurge the oxidative stress in cancerous cells. Our observations support our approach for the synthesis of effective inhibitors against MARK4 that can be taken forward for the development of novel anticancer molecules targeting MARK4.

## Introduction

Protein kinases are specifically being targeted in the design and development of new drugs^1,2^. Microtubule affinity regulating kinase 4 (MARK4) is a Ser/Thr kinase that comes under AMPK family and has recently been targeted for neurodegenerative diseases^3^, cancer^4^, obesity^5^ and associated metabolic disorders^6–8^. MARK4 was identified by its tau phosphorylating ability alongwith other microtubule associated proteins (MAPs) at unique Ser residues in KXGS motifs of microtubule binding repeats^9,10^. The role of MARK4 has mainly been studied in neurodegenerative disorders. Apart from the regulation of microtubule dynamics, it has versatile functions interfering with signal transduction, adipogenesis, cell polarity, cell cycle progression and positioning of organelle^11,12^.

Aberrant expression or dysregulation of MARK4 is linked with the development of a variety of diseases including different types of cancer like hepatocellular carcinoma, glioma and metastatic breast carcinomas^4,13,14^, neurological disorders like Alzheimer’s disease^3^, metabolic disorders including diet-induced obesity, cardiovascular diseases and type-II diabetes^12,15^. MARK4 also induces adipogenesis in adipocytes and stimulates apoptosis by JNK1 pathway^16^. These reports indicate that MARK4 may be a molecular target for cancer prevention or treatment interventions^17–19^.

**Figure 1:**
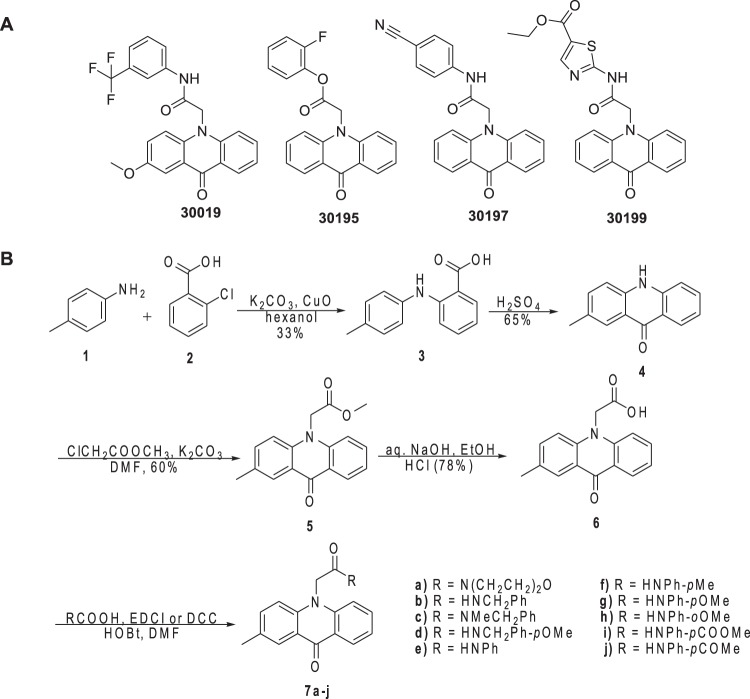
**(A)** MARK2-specific inhibitors. **(B)** Scheme for the synthesis of N-substituted acridone derivatives.

Acridones are an important class of heterocyclic compounds possessing various biological activities including anticancer^20^, antiherpes, antimalarial^21^, antileishmania^22^, and antibacterial^23^. Synthetic and naturally occurring acridones have been extensively investigated for their inhibitory effects against cathepsin^24,25^, kinases^26^, topoisomerase^27^, surviving^28^, acetylcholinesterase^29^, etc. In addition, acridones have been evaluated as modulators of P-gp mediated multidrug resistance^20^ and immunosuppressive agents^30^. In a campaign to discover probes capable of inhibiting MARK2 activity in cultured cells and primary neurons, Mandelkow and coworkers identified four compounds (30019, 30195, 30197, and 30199) sharing the acridone scaffold as MARK2-specific inhibitors with half maximal inhibitory concentration (IC_50_) values below 10 μM^31^. These lead structures provided a good basis for further studies on inhibition against MARK4 and evaluation of their anticancer properties by our group (Fig. 1A).

In the present work, we report the synthesis, characterization and biological evaluation of novel acridone derivatives as potential MARK4 inhibitors. It is shown that the synthesized compounds bind to the active site of MARK4 and display significant anticancer activities. The results of pharmacological studies by cell cytotoxicity, ROS quantification and apoptosis on MCF-7 cell line revealed that the selected acridones inhibit cell proliferation, elicit oxidative stress and induce apoptosis. Thus, these molecules can be used as lead compounds for the quest of cancer therapeutic agents in the near future.

## Results and Discussion

### Synthesis of *N*-substituted acridone derivatives

Guided by docking studies a series of *N*-substituted acridones were synthesized and evaluated as inhibitors of MARK4 phosphorylation. An Ullmann-type coupling of 2-chlorobenzoic acid **2** with *p*-toluidine provided 2-aminoarylbenzoic acid **3**, which was successively treated with H_2_SO_4_ to give 2-methylacridin-9(10*H*)-one **4** (Fig. 1B) in 65% yield^32,33^. Subsequently, *N*-alkylation of acridone **4** with methyl chloroacetate and K_2_CO_3_ as base under microwave irradiation at 100 °C furnished methyl 9-oxo-10(9*H*)-acridineacetate **5**, that was converted to the corresponding carboxylic acid **6** using NaOH in refluxing ethanol followed by acidification. The target compounds **7a-j** were prepared in good yields from acid **6** and the appropriate amines in anhydrous DMF, using ECDI or DCC as coupling agent.

### Selected compounds show significant binding with MARK4

**Figure 2:**
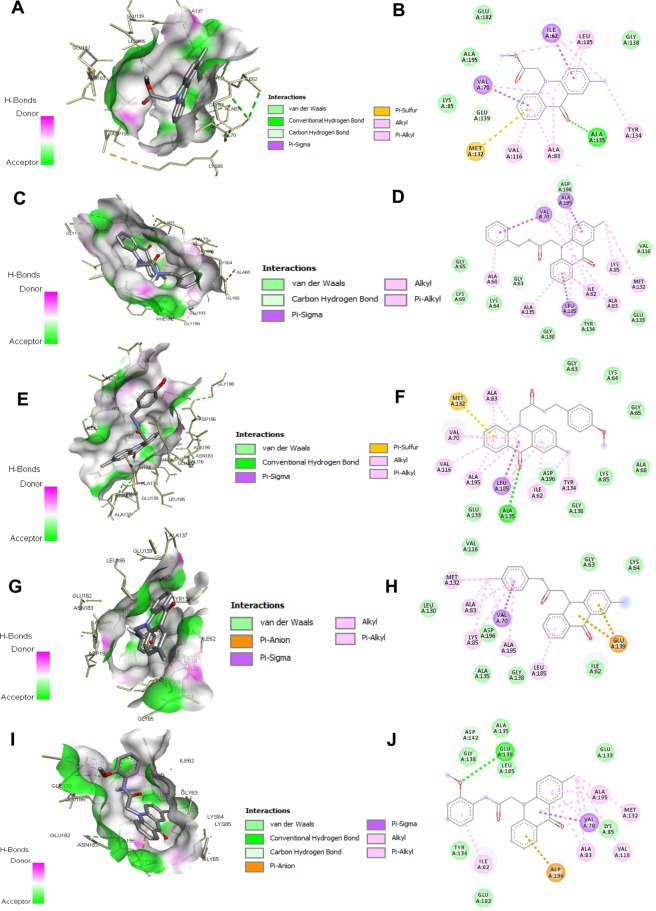
Molecular docking studies of selected compounds with MARK4: View of the catalytic pocket of MARK4 with **(A)** compound 5, **(C)** compound 7b, **(E)** compound 7d, **(G)** compound 7f, **(I)** compound 7h, shows the hydrogen bond donor-acceptor residues. 2D schematic representation of the docking model of **(B)** compound 5, **(D)** compound 7b, **(F)** compound 7d, **(H)** compound 7f, **(J)** compound 7 h with MARK4. Dotted lines in different colors reflected various types of interaction such as hydrogen bonding, charge or polar interactions, van der Waals and π-sigma interactions.

Molecular docking was carried out to predict existing molecular interactions between the synthesized acridones and amino acid residues of MARK4 utilizing Autodock, Autodock Vina^TM^ combined with PyRx^TM^ for workflow management^34,35^. The crystal structure of MARK4 with PDB code 5ES1 in the absence of 5RC module was selected and acridones were docked into the catalytic domain of the kinase. Data from docking experiments are presented in Table S1 and Figs 2, S1–S3. The MARK4-acridone complexes were stabilized by various non-covalent interactions offered by the residues present in the active site cavity of MARK4 (Figs 2, S1–S3). Further analysis of the docking results revealed that all the selected compounds showed binding energy ranging between –9.2 kcal/mol to –9.8 kcal/mol (Table S1).

Our first aim was to verify that the **5** occupies the catalytic center and second to design derivatives with increased interactions with the activation loop of MARK4 (e.g. DFG motif). All compounds **5** and **7a-j** form close interactions to active site residues of MARK4 including one hydrogen bond and several van der Waals interactions (Figs 2, S1–S3). The 2-methylacridone moiety off almost all tested compounds interacts by two different binding modes with the following amino acid residues: Ile62, Val70, Ala83, Val116, Met132, Glu133, Tyr134, Gly138, Glu139 and Leu185. In Fig. S4 two representative binding modes of acridones are illustrated: one pose with the methyl group to extend towards Ala83, Val116 and Met132 (compounds **5**, **7a**, **7b**, **7d**, **7e** and **7h**) and one pose with the same group to display interactions with Ile62 and Tyr134 (compounds **7c**, **7f**, **7g**, **7i** and **7j**). Notably, the carbonyl group of acridone forms one hydrogen bond with the amino group of Ala135 in the hinge region of kinase. Molecular docking studies revealed that compounds **7b**, **7f** and **7h** have the most promising binding energies (−9.5– −9.8 kcal/mol). Docking analysis illustrated that from these five analogues, all but **7f** get the same binding pose with the acridone’s methyl group interacting with Ala83, Val116 and Met132. The reliability of the applied docking protocol was assessed by re-docking the pyrazolopyrimidine inhibitor 5RC into the active site of the MARK4 (Fig. S5).

### Fluorescence binding studies

**Figure 3:**
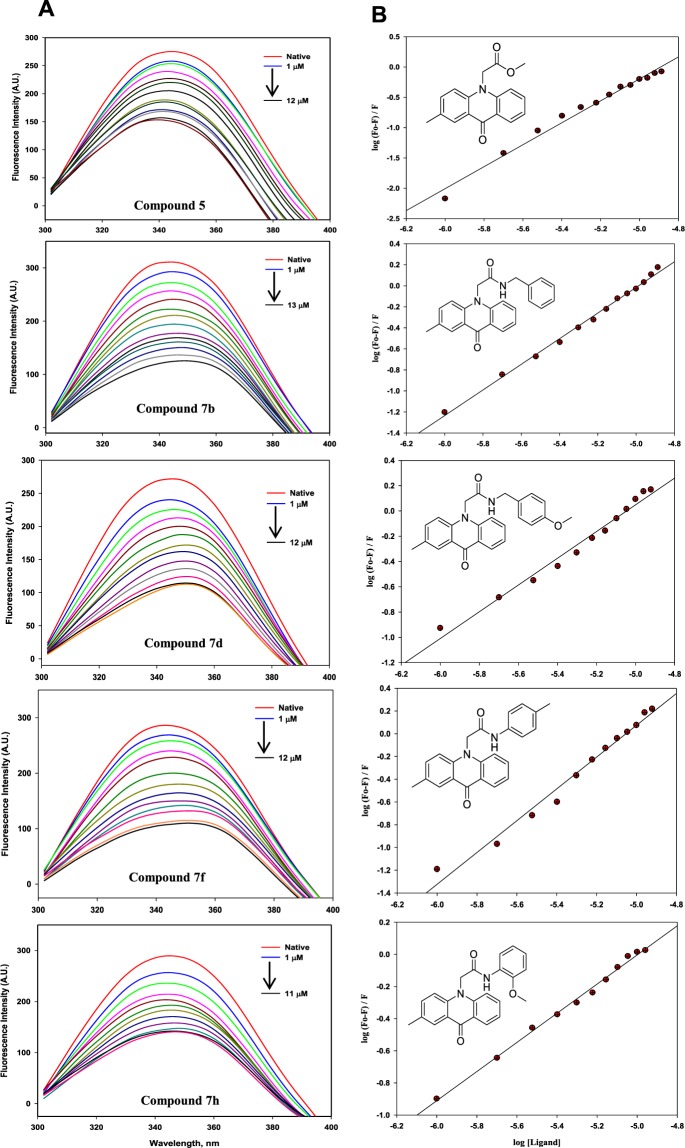
Fluorescence binding studies of compound 5, 7b, 7d, 7f and 7h with MARK4. **(A)** Fluorescence emission spectra of MARK4 (10 µM) with increasing concentrations of the respective compound (5, 7b, 7d, 7f and 7h). Excitation wavelength was fixed at 280 nm and emission was recorded in 300–400 nm range. **(B)** Modified Stern-Volmer plot obtained from fluorescence quenching of MARK4 by compound 5, 7b, 7d, 7f and 7h, respectively. It was used to calculate binding affinity (*K*a) and number of binding sites (*n*).

To determine the binding affinities of the synthesized acridones with MARK4, we used fluorescence emission spectra measurements. Protein sample was excited at 280 nm and emission was measured in 300–400 nm range with increasing concentrations of each acridone derivative (0–100 µM). A significant decrease in fluorescence intensity of MARK4 with increasing concentrations of each compound was fitted in the modified Stern-Volmer equation to calculate the binding constant, *K*_a_ and the number of binding sites per protein molecule (*n)*. All the synthesized acridones have been screened (Supplementary Fig. S6, S7 and Table S3) and those with the highest affinity have been selected (compounds 5, 7b, 7d, 7f and 7h) for further evaluation (Fig. 3). The binding constants of compound 5, 7b, 7d, 7f and 7h were estimated as 6.3 × 10^4^ M^−1^, 1.0 × 10^6^ M^−1^, 1.0 × 10^6^ M^−1^, 1.9 × 10^6^ M^−1^, and 3.1 × 10^5^ M^−1^, respectively (Fig. 3). Comparison of the measured binding affinities shows that 7b, 7d and 7 f exhibit the highest affinity towards MARK4. Additionally, it was found that each compound binds to a single binding site on MARK4.

### Enzyme inhibition assay and structure activity relationships

**Figure 4:**
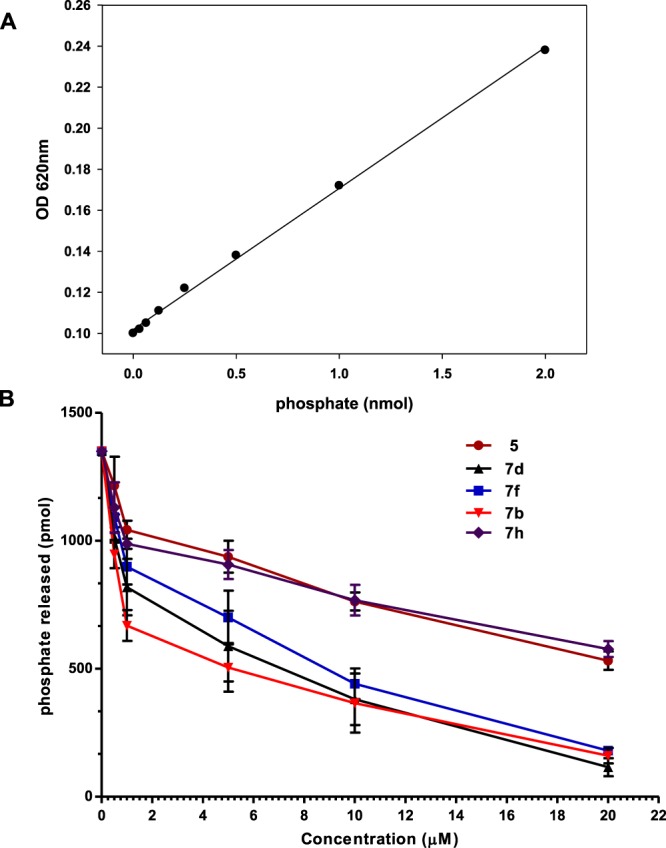
Enzyme activity profile of selected compounds with MARK4. **(A)** Standard phosphate hydrolysis curve was used to quantify the amount of phosphate released due to the kinase activity of MARK4. **(B)** ATPase inhibition (% hydrolysis of phosphate) with increasing concentrations of compound 5, 7b, 7d, 7f and 7h obtained by comparing with standard phosphate hydrolysis curve.

To evaluate the inhibition activity of synthesized acridone derivatives against recombinant MARK4, ATPase enzyme inhibition assay was performed (Table S3). Ester derivative **5** was active against MARK4 with an IC_50_ value of 14.12 ± 1.02 μM (Fig. 4). Our studies were initiated by replacing the ester group of **5** with sterically and electronically diverse amines affording the corresponding amides **7a–j**, in order to establish favorable interactions with residues Ala135, Val70, Asp196 and/or Glu182. The most potent compounds **7b** and **7d** in this screening carry a benzylamine (IC_50_ value of 1.80 ± 0.04 μM) and *p*-MeO-benzylamine (IC_50_ value of 2.20 ± 0.05 μM), respectively, whereas the methyl substituted benzylamide **7c** was much weaker inhibitor. These results are in accordance with docking studies, which show that the benzylamino moiety of **7b** and **7d** forms favorable interactions with residues Ala135, Val70, Ala68 and Gly63. Replacement of the benzylamine with morpholine resulted in a significant loss of potency, while compounds containing substituted phenylamines displayed variable activities. Compound **7e** was less potent than **7b**, while, introducing -OCOMe-, -COMe or -OMe as the C4-substituent of the phenylamino ring yielded compounds **7g**, **7i** and **7j** with lower activities and IC_50_ values higher than 20 μM. It should be noted that compound **7h** bearing an ortho-MeO-phenylamine was more active (IC_50_ = 12.15 ± 1.10 μM) than the para-MeO-phenylamino derivative **7g** (IC_50_ > 20.0 μM). In this case, the docking studies indicate a better fit of **7h** compared with **7g** in the active site of MARK4 with a binding energy of −9.5 kcal/mol and a stronger predicted hydrogen bond with Ala135 (3.06 Å for **7h**, 3.19 Å for **7g**). A *p*-methyl-phenylamine was favorable for the activity and **7f** inhibited MARK4 with an IC_50_ value of 4.5 ± 0.52 μM. It is interesting to note that the results of fluorescence binding are consistent with that of enzyme activity results suggesting that the high binding affinity compounds significantly inhibit the enzyme activity of MARK4.

### Isothermal titration calorimetry measurements

**Figure 5:**
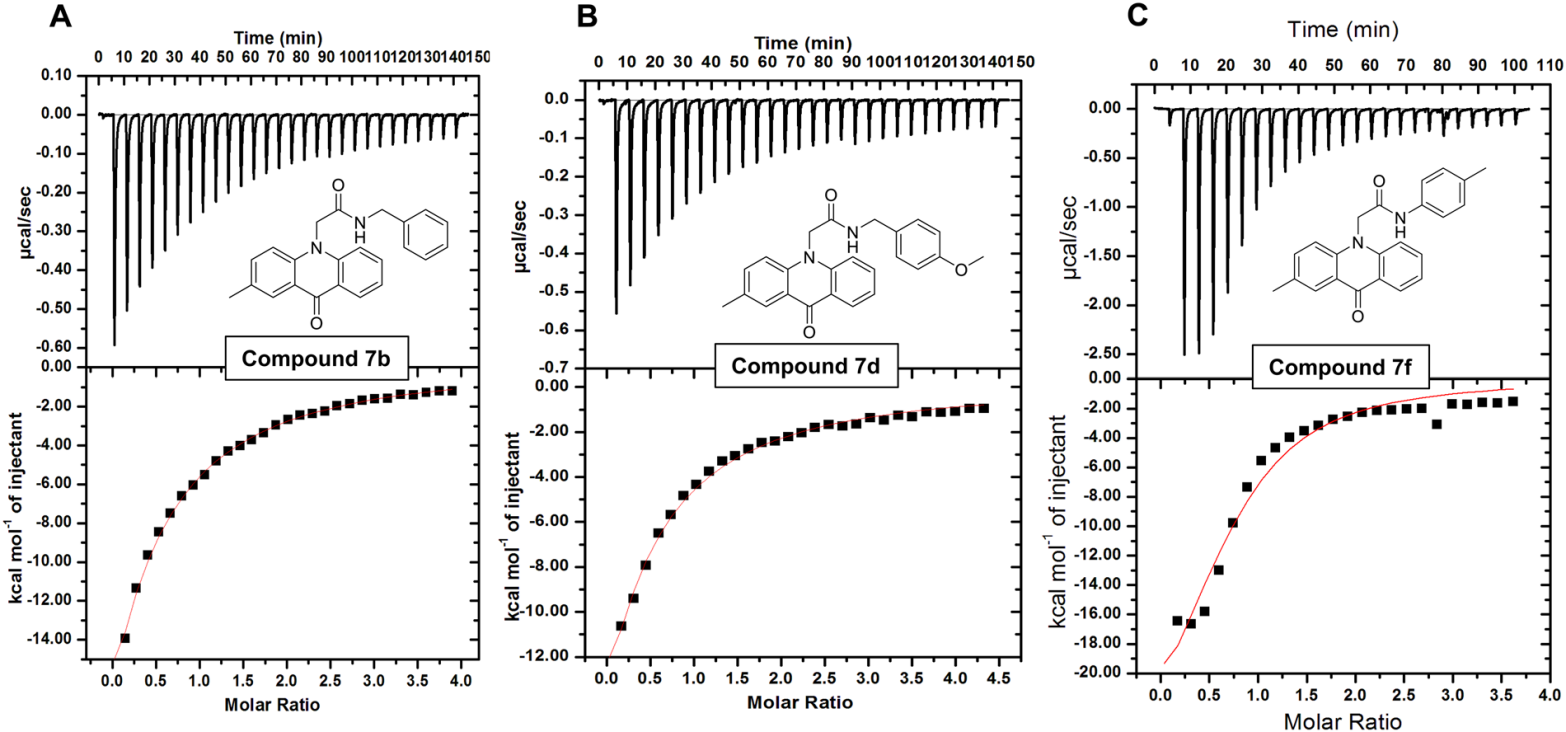
ITC measurement showing the titration of selected acridone derivatives with recombinant MARK4. (Top) Raw data plot of heat produced against time for the titration of 1000–1500 µM **(A)** compound **7b**, **(B)** compound **7d**, **(C)** compound **7f**, into 20–25 µM MARK4. (Bottom) Corresponding binding isotherm obtained after integration of peak area and normalization to yield a plot of molar enthalpy change against each compound-MARK4 ratio. The one-site fit curve is displayed as a thin red colour line.

Molecular docking, enzyme inhibition and fluorescence binding studies showed that compounds **5**, **7b**, **7d**, **7f** and **7h** are interacting to MARK4 and inhibit its activity. In order to measure the actual binding affinity and stoichiometry of selected compounds with the recombinant MARK4, isothermal titration calorimetry (ITC) has been carried out. A typical isotherm of ITC obtained after the titration of MARK4 with compound **7b**, **7d** and **7f** was shown in Fig. 5. Negative heat impulses in the upper panel of each isotherm indicate exothermic nature of binding. The amount of heat produced as a result of each injection helps to deliver the molar ratio of studied compound to that of MARK4. Thermodynamic parameters associated with binding of MARK4-compounds (*K*a, binding constant and Δ*H*, enthalpy change) are shown in Table 1. These results were obtained from single-site fitting model. We also tried ITC with compound **5** and **7h**, but at lower stoichiometry these compounds do not show significant binding and at high concentration they precipitate with MARK4. Overall inference from docking, fluorescence, ITC and enzyme inhibition suggested that the compound **5**, **7b**, **7d**, **7f** and **7h** bind with MARK4 and this binding is responsible for the inhibition of enzyme activity.

**Table 1:** Thermodynamic parameters and functional activity concentrations of MARK4 and selected acridone derivatives obtained from ITC, enzyme inhibition and MTT assays.

Compound number	^§^*K*_a_, (M^−1^)	Δ*H*, cal/mol	^¥^IC_50_, (µM), MARK4	^#^IC_50_, (µM), MCF-7
	6.39 × 10^4^ ± 4.33 × 10^3^	−1.82 × 10^9^ ± 1.23 × 10^7^	1.80 ± 0.04	5.2 ± 1.2
	6.32 × 10^4^ ± 7.00 × 10^3^	−1.75 × 10^7^ ± 2.37 × 10^5^	2.20 ± 0.05	6.3 ± 1.2
	1.35 × 10^5^ ± 3.8 × 10^2^	−3.01 × 10^4^ ± 590.10	4.5 ± 0.52	5.8 ± 1.4

^§^From ITC, ^¥^from enzyme inhibition, ^#^from MTT.

### Cell proliferation assay

Initially, all synthesized acridone derivatives were evaluated for their cytotoxicity potential on MCF-7, HepG2 and HEK293 cell lines by MTT assay. These synthesized acridone derivatives were screened in the concentration range of 0–200 µM, for 24 and 48 h. The results show that at higher concentrations all compounds inhibit the proliferation of MCF-7 as well as HepG2 cells (Table S1). Consistent with earlier studies, cell proliferation studies also showed that compounds **5**, **7b, 7d, 7f** and **7h** provoked superior toxicity in a concentration dependent manner on MCF-7 cells. It was also interesting that in the studied submicromolar concentration range these compounds don’t show considerable cytotoxicity towards HEK293 cell lines. The IC_50_ values for compound **5**, **7b**, **7d**, **7f** and **7h** were found to be 9.4 ± 1.0, 5.2 ± 1.20 μM, 6.3 ± 1.25 μM, 5.8 ± 1.4 μM, and 7.2 ± 1.33 μM, for MCF-7 cells. Cytotoxicity of these selected compounds at their respective IC_50_ value were also studied on HEK293 cells, it was observed that more than 85% of embryonic kidney cells were viable even after 72 h of treatment. Cytotoxicity results clearly suggested that the tested compounds are non-toxic to normal cells and specifically bear toxicity for cancerous cells. Thus, compounds **5**, **7b**, **7d**, **7f** and **7h** were taken for further cell based studies such as apoptosis and reactive oxygen species (ROS) production.

### Apoptosis assay

**Figure 6:**
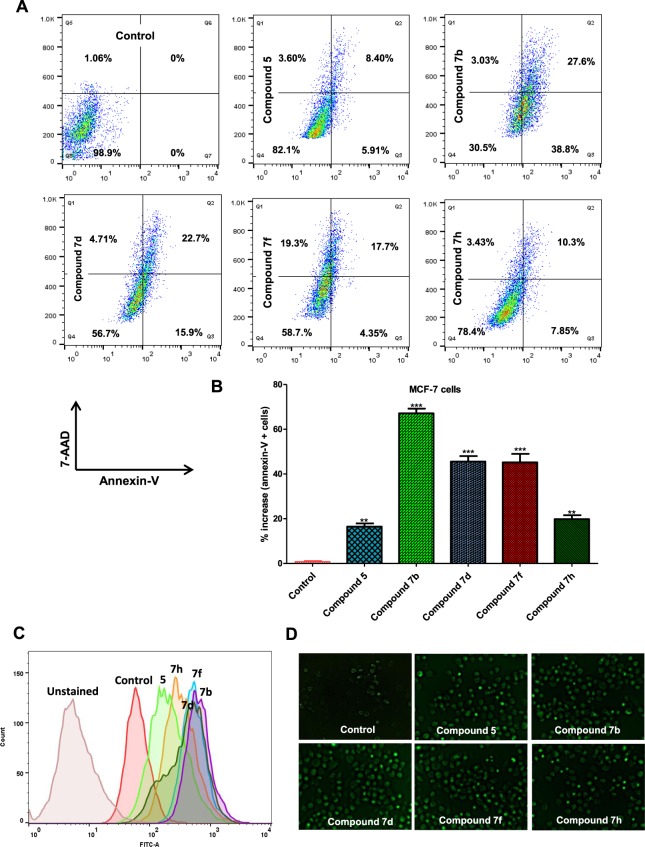
MARK4 inhibition by selected acridone derivatives induces apoptosis, elicited oxidative stress by inducing ROS production in MCF-7 cells. MCF-7 cells were treated with IC_50_ concentrations of each compound for 24 h and processed for apoptosis analysis using Annexin V-7AAD apoptosis kit by flow cytometry. (**A**) Quadrants showing the anti-FITC-Annexin-V stained cells after the treatment of each compound as indicated. (**B**) Bar graphs represents the percentage of apoptotic MCF-7 cells attained with Annexin-V for duplicate measurements ± SD. **p < 0.001, as compared to control (untreated cells). (For anticancer activities paclitaxel has been taken as positive control). (**C**) Histogram showing the fluorescence emission intensity of DCF as measured by flow cytometry, MCF-7 cells were treated with IC_50_ concentrations of each compound for 5–6 h and processed for ROS measurements using DCFDA staining and were quantified by flow cytometry. (**D**) Representative images of MCF-7 cells stained with DCFDA captured on fluorescence microscope (20 X original magnification) for the assessment of ROS after the treatment of respective compound and intensity of green color represents the levels of ROS.

Evasion of apoptosis is a striking hallmark of cancerous cells, it is an essential process that regulates abnormal growth of cells, but compromised signaling helps the cancerous cells to escape apoptosis^36^. MARK4 overexpression also supports the growth and evasion of cancerous cells^4^. Thus, the probability of apoptosis induction by inhibiting MARK4 was studied. The MCF-7 cells were serum starved and treated with IC_50_ concentration of each acridone derivative for 24 h and subsequently annexin-V staining was used to assess the apoptotic potential of these compounds. Stained cells were analyzed by flow cytometry and found that the treatment with compounds **5**, **7b**, **7d**, **7f** and **7h** considerably induces apoptosis in the MCF-7 cells (Fig. 6A). Analysis of the results suggested that treatment with compounds **5**, **7b**, **7d**, **7f** and **7h** induces apoptosis in 17.91%, 69.23%, 43.10%, 41.35%, and 21.53% of MCF-7 cells, respectively as compared to the control cells (Fig. 6B). Consistent to the results from binding, enzyme inhibition and cell proliferation tests, **7b** is active to a significantly higher extent (Fig. 6A,B). These results are consistent with previous observations which demonstrate that MARK4 as an inhibitor of hippo signalling in MCF-7 cells^3^ and negative regulator of mTORC1^12^ is a regulator of cell proliferation and migration of cancer cells.

### Selected acridones increase the levels of reactive oxygen species

The respiratory cycle of mitochondria is the foremost source of reactive oxygen species (ROS), and ROS has the potential to induce cell apoptosis^37^. Thus, it was speculated that the treatment with selected acridone derivatives might lead to the production of ROS. The MCF-7 cells were treated with the IC_50_ dose of compounds **5**, **7b**, **7d**, **7f** and **7h** for 5–6 h and ROS levels were quantified by flow cytometry using 2-dichlorofluorescein diacetate (DCFDA) staining. Interestingly, it was found that treatment with compound **5**, **7b**, **7d**, **7f** and **7h** increases the production of ROS. Representative histogram is shown in Fig. 6C, which suggests that incubation of MCF-7 cells with compounds **5**, **7b**, **7d**, **7f** and **7h** shifts the position of respective histogram towards right (higher value), that shows an increase in the levels of ROS.

Besides flow cytometry, levels of ROS were also measured by fluorescence spectroscopy, after treatment with compounds **5**, **7b**, **7d**, **7f** and **7h**, DCFDA staining and imaging on a fluorescence microscope (Fig. 6D). It is easily observed in the Fig. 6D that the intensifications of green fluorescence in case of treated cells denote the higher levels of ROS. Results of ROS measurement suggested that compound **5**, **7b**, **7d**, **7f** and **7h** considerably increase the levels of ROS that might be also a reason for cellular death of MCF-7.

Other important observation suggested by ROS experiments is that compounds **7b**, **7d** and **7f** induce ROS to a higher extent than compound **5** and **7h**. Generation and accumulation of ROS results in oxidative stress and plays a crucial role in the governing of cancer cell behavior. Higher levels of ROS trigger an array of pro-apoptotic signal pathways, such as mitochondrial dysfunction and ER stress and, which in due course leads to worsening of cell function and apoptosis^37^.

### Kinase inhibitor selectivity

**Figure 7:**
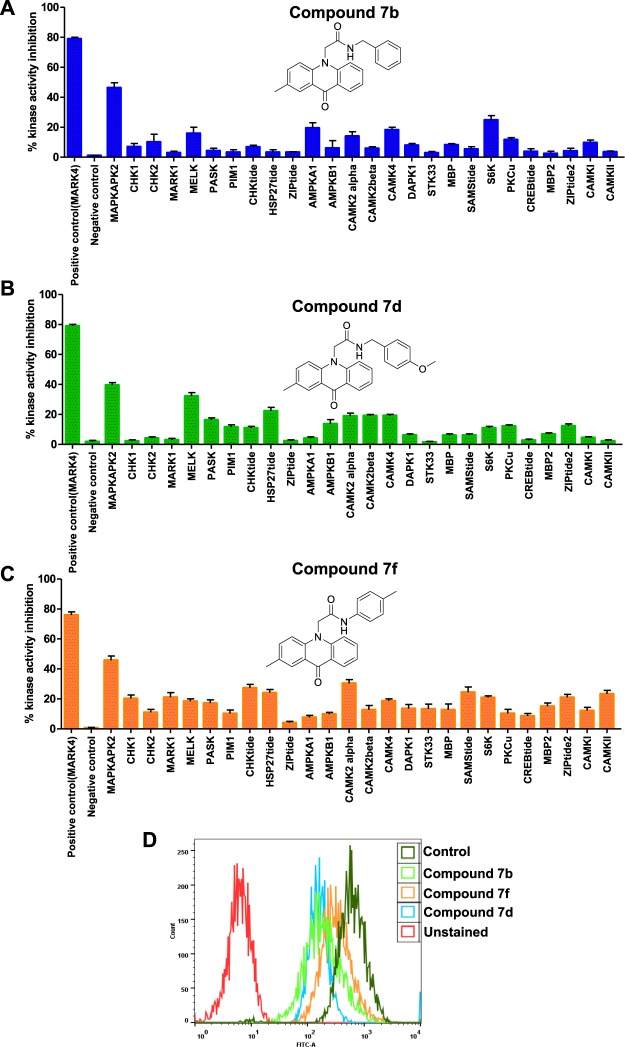
Kinase selectivity and tau-phosphorylation assay. Kinase selectivity results of **(A)** compound 7b, **(B)** compound 7d, **(C)** compound 7f. The selected panel of kinases was incubated with 10 µM dose of selected compounds and analyzed for percent activity values. The percent kinase activity was calculated in the compound-containing reactions and plotted in terms of percent kinase activity inhibition. **(D)** Representative flow cytometry histogram of SH-SY5Y cell fractions stained with phosphorylated anti-tau antibodies, each histogram represents the phosphorylation status of tau under different treatments as mentioned in inset.

The selectivity of kinase inhibitors is commonly evaluating them against a panel of closely related kinases based on the hypothesis that off-target interfaces are more likely to be associated with same/different families of kinases with similar amino acid sequence. So, we evaluated the fraction of kinase targets that are within the same kinase family (CAMK family). The results of within-family selectivity of MARK-4 targeting compounds suggested that at a single dose of 10 µM, compound **7b**, **7d** and **7f** more strongly inhibits MARK4 as compared to other kinases of same family (Fig. 7A–C). It was clearly observed that, though selected compounds inhibit other kinases also, but non-significantly. The percent inhibition shows that for other kinases the IC_50_ value will be higher than 10 µM, as at this dose none of the kinase inhibited more/equal than 50 percent (Fig. 7A–C). These kinase selectivity results signify the use of these compounds as potential and selective inhibitors towards MARK4.

### Tau phosphorylation studies

To see the effect of most potential lead molecules (compound **7b, 7d** and **7f**) on the substrate protein (tau protein) of MARK4 enzyme inhibition activity studies are extended to a cell-based tau-phosphorylation assay. Cells were allowed to grow in the presence of IC_50_ dose of compound **7b, 7d** and **7f** for 24 hrs. Following the treatment, the phosphorylation of tau has been assessed with the help of flow cytometry. Results of tau-phosphorylation assay suggested that the compound **7b, 7d** and **7f** decreases the phosphorylation level of tau protein (Fig. 7D). The outcomes of tau-phosphorylation assay as shown in Fig. 7D revealed that treatment of compounds **7b, 7d** and **7f** moves the histogram towards the lower side of untreated/control cells (shown by dark green color). These results suggested that the selected compounds inhibit the phosphorylation of tau and also clues the inhibition of MARK4 as tau is a substrate for MARK4.

### Conclusions

Conclusively, in this study it was perceived that the studied acridone derivatives significantly bind to the active site cavity of MARK4 that might be responsible for enzyme inhibition. The selected lead molecules also show the specificity towards MARK4. Inhibition of MARK4 hinders the proliferation of cancerous cells, enhance ROS generation and induce apoptosis. Results obtained from this study clearly support our assumption and previous studies that MARK4 will be an effective anticancer drug target. Furthermore, our findings indicate that acridone scaffold may be further employed in the discovery of potential MARK4 inhibitors, with an anticancer pharmacological profile, that will help to counter the progression of cancer and other MARK4 allied disorders.

## Materials and Methods

### Materials

Luria broth, Miller and Luria agar Miller for bacterial culture were obtained from BD Difco, USA. Ni-NTA column were purchased from GE healthcare (GE Healthcare Life Sciences, Uppsala, Sweden). Human embryonic kidney (HEK293) cells,), human hepatic cancer cells (HepG2), human neuroblastoma cells (SH-SY5Y) and human breast cancer cell lines (MCF-7) were obtained from National Centre for Cell Sciences, Pune, India. TrypLE express cell detachment enzyme, antibiotic cocktail, Dulbecco’s modified eagle’s media, DCFDA and fetal bovine serum and were obtained from Invitrogen/Gibco-life technologies, Carlsbad, California, United States. FITC-Annexin-V apoptosis detection kit was procured from BD-Pharmingen, BD Biosciences (USA).

### General experimental details

All reactions were carried out under an atmosphere of Ar unless otherwise specified. Commercial reagents of high purity were purchased and used without further purification, unless otherwise noted. Reactions were monitored by TLC and using UV light as a visualizing agent and aqueous ceric sulfate/phosphomolybdic acid, ethanolic *p*-anisaldehyde solution, potassium permanganate solution, and heat as developing agents. The ^1^H and ^13^C NMR spectra were recorded at 500 and 125 MHz, and tetramethylsilane was used as an internal standard. Chemical shifts are indicated in δ values (ppm) from internal reference peaks (TMS ^1^H 0.00; CDCl_3_^1^H 7.26, ^13^C 77.00; DMSO-*d*_6_^1^H 2.50, ^13^C 39.51). Melting points (mp) are uncorrected. High-resolution mass spectra (HRMS) were recorded by direct injection of a 2 μM (2 μL) solution of the compounds in water–acetonitrile (1/1; v/v) and 0.1% formic acid on a mass spectrometer (hybrid ion trap-orbitrap mass spectrometer) equipped with an electrospray ion source in positive mode (source voltage 3.5 kV, sheath gas flow 10, capillary temperature 275 °C) with resolution R = 60.000 at m/z = 400 (mass range = 150–2000) and dioctylphthalate (m/z = 391.28428) as the “lock mass”.

#### 2-(*p*-Tolylamino)benzoic acid, 3

A mixture of *p*-toluidine (1 g, 9.33 mmol), o-chlorobenzoic acid (1.285 g, 8.21 mmol), dry potassium carbonate (3.5 g, 25.32 mmol) and copper oxide (16.5 mg, 0.21 mmol) in 11 mL hexanol was heated at 140 °C for 5 h on an oil bath, under atmosphere of Ar. The reaction mixture was allowed to cool at 0 °C and then the pH was adjusted to 4–5 with 2 M HCl. The mixture was left overnight at room temperature. The brown solid was filtered off, washed with hot H_2_O and dried. The mixture was extracted with dichloromethane and the combined organic extracts were dried over Na_2_SO_4_, filtered, and concentrated under reduced pressure. The solid was further purified by recrystallization with a mixture of MeOH/H_2_O = 1/2 to afford 615 mg of colorless crystals in 33% yield. Characterization was in agreement with literature data [1]. **3**: ^1^H-NMR (500 MHz, CDCl_3_) δ 9.23 (brs, 1 H, -NH), 8.04 (dd, *J* = 8.0, 1.4 Hz, 1 H, =CH), 7.33 (m, 1 H, =CH), 7.21–7.11 (m, 5 H, =CH), 6.72 (t, *J* = 7.5 Hz, 1 H, =CH), 2.37 (s, 3 H, -CH_3_); ^13^C-NMR (126 MHz, CDCl_3_) δ 173.6 (-C=O), 149.6 (-C=C-), 137.6 (-C=C-), 135.2 (-C=C-), 134.1 (-C=C-), 132.5 (-C=C-), 130.0 (-C=C-), 123.8 (-C=C-), 116.7(-C=C-), 113.8(-C=C-), 109.9(-C=C-), 20.9 (-CH_3_).

#### 2-Methylacridin-9(10*H*)-one, 4

2-(*p*-tolylamino)benzoic acid (500 mg, 2.20 mmol) and 2.5 ml of conc. sulfuric acid were heated at 100 °C for 2 h. Appearance of green color indicated the completion of the reaction. After cooling, the green-brown solution was poured into cold water (40 mL), made alkaline by liquor ammonia and was allowed to stand for 12 h at rt. The green-yellow solid was collected by filtration, washed with H_2_O and dried under reduced pressure to give 229 mg of **4**. The filtrate was extracted with dichloromethane, the extract was dried over Na_2_SO_4_ and concentrated under reduced pressure to afford additional 69 mg of **4** (total yield 65%). Characterization was in agreement with literature data [1]. **4:** ^1^H-NMR (500 MHz, DMSO-*d*_6_) δ 11.65 (s, 1 H, -NH), 8.22 (d, *J* = 8.0 Hz, 1 H, =CH), 8.02 (brs, 1 H, =CH), 7.70 (t, *J* = 7.5 Hz, 1 H, =CH), 7.59–7.50 (m, 2 H, =CH), 7.46 (d, *J* = 8.4 Hz, 1 H, =CH), 7.23 (t, *J* = 7.5 Hz, 1 H, =CH), 2.42 (s, 3 H, -CH_3_); ^**13**^C-NMR (126 MHz, DMSO-*d*_6_) δ 176.5 (-C=O), 140.8 (-C=C-), 139.0 (-C=C-), 134.9 (-C=C-), 133.2 (-C=C-), 130.0 (-C=C-), 126.0 (-C=C-), 125.0 (-C=C-), 120.7 (-C=C-), 120.4 (-C=C-), 120.3 (-C=C-), 117.3 (-C=C-), 117.2 (-C=C-), 21.0 (-CH_3_).

#### Methyl 2-(2-methyl-9-oxoacridin-10(9*H*)-yl)acetate, 5

To a 2-methylacridin-9(10 *H*)-one (150 mg, 0.72 mmol) suspension in dry DMF (6 mL), 995 mg of dry potassium carbonate (7.20 mmol) and 406 mg of methyl chloroacetate (3.74 mmol, 0.33 mL) were added and the mixture was stirred under microwave irradiation at 100 °C for 2 h. The reaction mixture was extracted with dichloromethane and the combined organic extracts were dried over Na_2_SO_4_, filtered, and concentrated under reduced pressure. The residue was purified by flash column chromatography (eluent; dichloromethane) to afford 120 mg of **5** in 60% yield. **5:** mp = 153–159 °C; ^1^H-NMR (500 MHz, CDCl_3_) δ 8.47 (d, *J* = 7.8 Hz, 1 H, =CH), 8.33 (s, 1 H, =CH), 7.61 (t, *J* = 7.5 Hz, 1 H, =CH), 7.43 (d, *J* = 8.5 Hz, 1 H, =CH), 7.22 (m, 2 H, =CH), 7.14 (d, *J = *8.5 Hz, 1 H, =CH), 4.99 (s, 2 H, -CH_2_-), 3.77 (s, 3 H, -OCH_3_), 2.39 (s, 3 H, -CH_3_); ^13^C-NMR (126 MHz, CDCl_3_) δ 178.0 (-C=O), 168.9 (-C=O), 142.1 (-C=C-), 140.3 (-C=C-), 135.4 (-C=C-), 133.9 (-C=C-), 131.6 (-C=C-), 128.0 (-C=C-), 127.3 (-C=C-), 122.5 (-C=C-), 122.4 (-C=C-), 121.6 (-C=C-), 114.1 (-C=C-), 114.0 (-C=C-), 52.9 (-OCH_3_), 48.2 (-CH_2_-), 20.6 (-CH_3_); FT-IR (KBr) 2923 (=C-H), 2845(-C-H), 1747 (-C=O), 1637 (-C=O-), 1616 (-C=C-), 1600 (-C=C-), 1510 (-C=C-), 1490 (-C-H), 1456 (-C-H), 1433 (-C-H), 1365 (-C-H), 1222 (C-O-C), 1211 (C-O) cm^−1^; ESI-HRMS m/z for C_17_H_16_NO_3_ [M + H]^+^ calcd 282.1125; found 282.1119.

#### 2-(2-Methyl-9-oxoacridin-10(9*H*)-yl)acetic acid, 6

Methyl 2-(2-methyl-9-oxoacridin-10(9 *H*)-yl)acetate (50 mg, 0.18 mmol) was dissolved in 3.5 mL of boiling ethanol. Then, 0.1 mL of 7.5 N NaOH was added and the mixture was refluxed for 0.5 h. After 10 min a yellow solid precipitated. The reaction mixture was concentrated under reduced pressure. The resulting sodium salt was dissolved in 0.5 mL H_2_O and the solution acidified with 2 M HCl to pH 1. The precipitate was filtered off, washed with cold H_2_O and dried. The crude product was recrystallized from a DMF/H_2_O = 1/2 mixture. The product was then collected by filtration as a yellow solid (37 mg, 78% yield). **6:** ^1^H-NMR (500 MHz, DMSO-*d*_6_) δ 8.35 (d, *J* = 7.8 Hz, 1 H, =CH), 8.14 (s, 1 H, =CH), 7.79 (t, *J* = 7.3 Hz, 1 H, =CH), 7.61 (m, 3 H, =CH), 7.33 (t, *J* = 7.3 Hz, 1 H, =CH), 5.30 (s, 2 H, -CH_2_-), 2.43 (s, 3 H, -CH_3_); ^13^C-NMR (126 MHz, DMSO-*d*_6_) δ 176.5 (-C=O), 170.0 (-C=O), 142.0 (-C=C-), 140.3 (-C=C-), 135.5 (-C=C-), 134.0 (-C=C-), 130.8 (-C=C-), 126.6 (-C=C-), 125.8 (-C=C-), 121.43 (-C=C-), 121.4 (-C=C-), 121.3 (-C=C-), 115.8 (-C=C-), 115.6 (-C=C-), 47.4 (-CH_2_-), 20.2 (-CH_3_); ESI-HRMS m/z for C_16_H_14_NO_3_ [M + H]^+^ calcd 268.0968; found 268.0967.

#### 2-Methyl-10-(2-morpholino-2-oxoethyl)acridin-9(10*H*)-one, 7a

To a solution of 2-(2-methyl-9-oxoacridin-10(9 *H*)-yl)acetic acid **6** (20 mg, 0.075 mmol) in 0.2 mL of dry DMF, morpholine (13 μL, 0.15 mmol), DCC (19 mg, 0.09 mmol) and HOBt hydrate (12 mg, 0.09 mmol) were added. The reaction mixture was stirred at room temperature for 48 h. The precipitate was removed by filtration and washed with DCM. The solvent was evaporated in vacuo and the residue was purified by flash column chromatography (eluent; petroleum ether/ethyl acetate = 3/2) to give 15 mg of **7a** in 60% yield as a yellow solid. Decomposition temperature = 190 °C; ^1^H-NMR (500 MHz, CDCl_3_) *δ* 8.51 (d, *J* = 7.8 Hz, 1 H, =CH), 8.29 (s, 1 H, =CH), 7.62 (t, *J* = 7.4 Hz, 1 H, =CH), 7.45 (d, *J* = 7.6 Hz, 1 H, =CH), 7.28 (m, 1 H, =CH), 7.10 (d, *J* = 8.5 Hz, 1 H, =CH), 7.02 (d, *J* = 8.5 Hz, 1 H, =CH), 5.03 (s, 2 H, -CH_2_-), 3.84–3.69 (m, 8 H, -OCH_2_-, -NCH_2_-), 2.42 (s, 3 H, -CH_3_); ^13^C-NMR (126 MHz, CDCl_3_) δ 178.0 (-C=O), 164.9 (-C=O), 142.3 (-C=C-), 140.5 (-C=C-), 135.4 (-C=C-), 133.9 (-C=C-), 131.4 (-C=C-), 127.9 (-C=C-), 127.1 (-C=C-), 122.4 (-C=C-), 122.3 (-C=C-), 121.5 (-C=C-), 114.2 (-C=C-), 114.1 (-C=C-), 66.9 (-CH_2_-), 66.6 (-CH_2_-), 48.3 (-CH_2_-), 45.5 (-CH_2_-), 42.6 (-CH_2_-), 20.6 (-CH_3_); FT-IR (KBr) 2924 (=C-H), 2852 (-C-H), 1736 (-C=O), 1648 (-C=O), 1614 (-C=C-), 1598 (-C=C-), 1491 (-C-H), 1465 (-C-H), 1432 (-C-H), 1370 (-C-H), 1339 (-C-H), 1303 (-C-O), 1284 (-C-O), 1232 (-C-N), 1179 (-C-O), cm^−1^; ESI-HRMS m/z for C_20_H_21_N_2_O_3_ [M + H]^+^ calcd 337.1547, found 337.1550.

#### *N*-benzyl-2-(2-methyl-9-oxoacridin-10(9*H*)-yl)acetamide, 7b

To a solution of 2-(2-methyl-9-oxoacridin-10(9 *H*)-yl)acetic acid **6** (40 mg, 0.15 mmol) in 1 mL of dry DMF, benzylamine (25 μl, 0.23 mmol), EDCI (43 mg, 0.225 mmol) and HOBt hydrate (35 mg, 0.225 mmol) were successively added. The reaction mixture was stirred at room temperature for 24 h and then it was concentrated under reduced pressure. The product was purified by crystallization using a mixture of DCM/acetone/MeOH = 1/0.2/0.1. The crystals were then collected by filtration, washed with dichloromethane and petroleum ether. The product was isolated as a yellow solid in 71% yield (38 mg). **7b**: mp = 277–280 °C; ^1^H-NMR (500 MHz, CDCl_3_) δ 8.14 (d, *J* = 7.8 Hz, 1 H, =CH), 7.92 (s, 1 H, =CH), 7.69 (m, 1 H, =CH), 7.51 (dd, *J* = 8.7, 2.2 Hz, 1 H, =CH), 7.34 (d, *J* = 8.7 Hz, 1 H, =CH), 7.28–7.11 (m, 6 H, =CH), 7.03 (s, 1 H, N-H), 4.99 (s, 2 H, -CH_2_-), 4.55 (d, *J* = 6.1 Hz, 2 H, -CH_2_N-), 2.35 (s, 3 H, -CH_3_); ^13^C-NMR (126 MHz, CDCl_3_) δ 177.8 (-C=O), 167.4 (-C=O), 141.9 (-C=C-), 140.1 (-C=C-), 137.8 (-C=C-), 135.7 (-C=C-), 134.1 (-C=C-), 131.9 (-C=C-), 128.6 (-C=C-), 127.9 (-C=C-), 127.6 (-C=C-), 127.5 (-C=C-), 127.2 (-C=C-), 122.4 (-C=C-), 122.3 (-C=C-), 122.0 (-C=C-), 114.3 (-C=C-), 144.2 (-C=C-), 51.4 (-CH_2_N-), 43.5 (-CH_2_N-), 20.6 (-CH_3_); FT-IR (KBr): 3293 (N-H), 3031 (=C-H), 2928 (-C-H), 2850 (-C-H), 1654 (-C=O), 1633 (-C=O), 1615 (-C=C-), 1598 (-C=C-), 1534 (-C=C-), 1488 (-C-H), 1467 (-C-H), 1454 (-C-H), 1427(-C-H), 1357 (-C-H), 1290 (-C-O), 1269 (-C-O), 1232 (-C-N), 1180 (-C-O) cm^−1^; ESI-HRMS m/z for C_23_H_21_N_2_O_2_ [M + H]^+^ calcd 357.1598, found 357.1591.

#### *N-*benzyl-*N*-methyl-2-(2-methyl-9-oxoacridin-10(9 *H*)-yl)acetamide, 7c

To a solution of 2-(2-methyl-9-oxoacridin-10(9 *H*)-yl)acetic acid **6** (40 mg, 0.15 mmol) in 1 mL of dry DMF, *N*-methylbenzylamine (29 μL, 0.23 mmol), EDCI (43 mg, 0.225 mmol) and HOBt hydrate (35 mg, 0.225 mmol) were successively added. The mixture was stirred at room temperature for 24 h. The solvent was evaporated in vacuo and the residue was purified by flash columm chromatography (eluent: petroleum ether/ethyl acetate = 2/1) to give 25 mg of **7c** as a yellow solid (yield = 45%). **7c**: mp = 131–141 °C; mixture of rotamers: ^1^H-NMR (500 MHz, DMSO-*d*_6_) *δ* 8.35 (d, *J* = 8.2 Hz, 1 H, =CH), 8.15 (s, 1 H, =CH), 7.78 (t, *J* = 7.8 Hz, 1 H, =CH), 7.61 (m, 2 H, =CH), 7.56–7.52 (m, 2 H, =CH), 7.38 (m, 2 H, =CH), 7.34–7.27 (m, 3 H, =CH), 5.56 (s, 2 H, -CH_2_-), 4.57 (s, 2 H, -CH_2_-), 3.24 (s, 3 H, -CH_3_), 2.44 (s, 3 H, -CH_3_); ^13^C-NMR (126 MHz, DMSO-*d*_6_) *δ* 176.7 (-C = O), 166.6 (-C=O), 142.4 (-C=C-), 140.7 (-C=C-), 137.5 (-C=C-), 135.3 (-C=C-), 133.8 (-C=C-), 130.6 (-C=C-), 128.6 (-C=C-), 127.5 (-C=C-), 127.2 (-C=C-), 127.0 (-C=C-), 126.6 (-C=C-), 125.7 (-C=C-), 121.5 (-C=C-), 121.1 (-C=C-), 116.2 (-C=C-), 116.0 (-C=C-), 50.7 (-CH_2_-), 47.3 (-CH_2_-), 34.2 (-CH_3_), 20.3 (-CH_3_); FT-IR (KBr): 3024 (=C-H), 2923 (-C-H), 2853 (-C-H), 1652 (-C=O), 1635 (-C=O), 1598 (-C=C-), 1489 (-C-H), 1466 (-C-H), 1406 (-C-H), 1370 (-C-H), 1288 (-C-O), 1207 (-C-O), 1183 (-C-N), 1118 (-C-O) cm^−1^; ESI-HRMS m/z for C_24_H_23_N_2_O_2_ [M + H]^+^ calcd 371.1754, found 371.1758.

#### *N*-(4-methoxybenzyl)-2-(2-methyl-9-oxoacridin-10(9*H*)-yl)acetamide, 7d

To a solution of 2-(2-methyl-9-oxoacridin-10(9 *H*)-yl)acetic acid **6** (30 mg, 0.112 mmol) in 0.75 mL of dry DMF, *p*-methoxybenzylamine (22 μL, 0.168 mmol), EDCI (32 mg, 0.168 mmol) and HOBt hydrate (26 mg, 0.168 mmol) were successively added. The mixture was stirred at room temperature for 24 h and then it was concentrated under reduced pressure. The product was purified by crystallization using a mixture of DCM/acetone/MeOH (1/0.2/0.1). The crystals were collected by filtration, washed with DCM and petroleum ether. The product was isolated as a yellow solid in 75% yield (33 mg). **7d**: mp = 258–261 °C; ^1^H-NMR (500 MHz, CDCl_3_) *δ* 8.23 (d, *J* = 8.1 Hz, 1 H, =CH), 8.00 (s, 1 H, Ν-Η), 7.69 (t, *J* = 7.8 Hz, 1 H, =CH), 7.51 (d, *J* = 8.8 Hz, 1 H, =CH), 7.33 (d, *J = *8.7 Hz, 1 H, =CH), 7.24 (m, 1 H, overlapping with CDCl_3_, =CH), 7.19 (t, *J* = 7.4 Hz, 1 H, =CH), 7.09 (d, *J* = 8.2 Hz, 2 H, =CH), 6.76 (d, *J* = 8.2 Hz, 3 H, =CH), 4.98 (s, 2 H, -CH_2_-), 4.46 (d, *J* = 5.9 Hz, 2 H, -CH_2_-), 3.75 (s, 3 H, -OCH_3_), 2.38 (s, 3 H, -CH_3_); ^13^C-NMR (126 MHz, CDCl_3_) *δ* 177.7 (-C=O), 167.2 (-C=O), 159.0 (-C=C-), 142.0 (-C=C-), 140.1 (-C=C-) 135.8 (-C=C-), 134.2 (-C=C-), 132.1 (-C=C-), 128.7 (-C=C-), 128.9 (-C=C-), 128.0 (-C=C-), 127.3 (-C=C-), 122.4 (-C=C-), 122.1 (1 C missing due to overlapping, -C=C-), 114.3 (-C=C-), 114.2 (-C=C-), 114.0 (-C=C-), 55.3 (-OCH_3_), 51.5 (-CH_2_N-), 43.0 (-CH_2_-), 20.6 (-CH_3_); FT-IR (KBr) 3281 (N-H), 3069 (=C-H), 2921 (-C-H), 2834 (-C-H), 1644 (-C=O), 1605 (-C=C-), 1541 (-C=C-), 1515 (-C=C-), 1486 (-C-H), 1464 (-C-H), 1367 (-C-H), 1304 (-C-H), 1289 (-C-O), 1248 (-C-O), 1184 (-C-N), 1114 (-C-O) cm^−1^; ESI-HRMS m/z for C_24_H_23_N_2_O_3_ [M + H]^+^ calcd 387.1703, found 387.1697.

#### 2-(2-Methyl-9-oxoacridin-10(9*H*)-yl)-*N*-phenylacetamide, 7e

To a solution of 2-(2-methyl-9-oxoacridin-10(9 *H*)-yl)acetic acid **6** (40 mg, 0.15 mmol) in 1 mL of dry DMF, aniline (21 μL, 0.23 mmol), EDCI (43 mg, 0.225 mmol) and HOBt hydrate (35 mg, 0.225 mmol) were successively added. The reaction mixture was stirred at room temperature for 24 h. The solvent was evaporated *in vacuo* and the product was purified by crystallization using a mixture DCM/acetone/MeOH (1/0.2/0.1). The crystals were collected by filtration and washed with petroleum ether. The product was isolated as a yellow solid in 80% yield (41 mg). **7e:** mp: 290–293 °C; ^1^H-NMR (500 MHz, DMSO-*d*_6_) δ 10.60 (s, 1 H, -NH), 8.36 (d, *J* = 8.0 Hz, 1 H, =CH), 8.16 (s, 1 H, =CH), 7.79 (t, *J* = 7.8 Hz, 1 H, =CH), 7.71–7.59 (m, 5 H, =CH), 7.33 (m, 3 H, =CH), 7.08 (t, *J* = 7.8, 1 H, =CH), 5.38 (s, 2 H, -CH_2_-), 2.44 (s, 3 H, -CH_3_); ^13^C-NMR (126 MHz, DMSO-*d*_6_) δ 176.5 (-C=O), 165.8 (-C=O), 142.4 (-C=C-), 140.6 (-C=C-), 138.6 (-C=C-), 135.4 (-C=C-), 133.9 (-C=C-), 130.6 (-C=C-), 128.8 (-C=C-), 126.5 (-C=C-), 125.7 (-C=C-), 123.6 (-C=C-), 121.4 (-C=C-), 121.3 (-C=C-), 121.2 (-C=C-), 119.1 (-C=C-), 115.8 (-C=C-), 115.7 (-C=C-), 49.1 (-CH_2_-), 20.2 (-CH_3_); FT-IR (KBr) 3258 (N-H), 3198 (=C-H), 3135(=C-H), 3078 (=C-H), 2913 (-C-H), 2850 (-C-H), 1666 (-C=O), 1634 (-C=O), 1617 (-C=C-), 1596 (-C=C-), 1551 (-C=C-), 1500 (-C=C-), 1489 (-C=C-), 1459 (-C-H), 1445 (-C-H), 1365 (-C-H), 1282 (-C-H), 1257 (-C-O), 1212 (-C-N), 1184 (-C-O) cm^−1^; ESI-HRMS m/z for C_22_H_19_N_2_O_2_ [M + H]^+^ calcd 343.1441, found 343.1443.

#### 2-(2-Methyl-9-oxoacridin-10(9*H*)-yl)-*N*-(*p*-tolyl)acetamide, 7f

To a solution 2-(2-methyl-9-oxoacridin-10(9 *H*)-yl)acetic acid **6** (40 mg, of 0.15 mmol) in 1 mL of dry DMF, *p*-toluidine (24 mg, 0.23 mmol), EDCI (43 mg, 0.225 mmol) and HOBt hydrate (35 mg, 0.225 mmol) were successively added. The reaction mixture was stirred at room temperature for 24 h and then it was concentrated under reduced pressure. The product was purified by crystallization using a mixture of DCM/acetone/MeOH (1/0.2/0.1). The crystals were collected by filtration, washed with dichloromethane and petroleum ether. The product was isolated as a yellow solid in 47% yield (25 mg). **7 f**: mp = 306–309 °C; ^1^H-NMR (500 MHz, DMSO-*d*_6_) δ 10.49 (s, 1 H, -NH), 8.35 (d, *J* = 7.6 Hz, 1 H, =CH), 8.15 (s, 1 H, =CH), 7.79 (t, *J* = 7.0 Hz, 1 H, =CH), 7.71–7.58 (m, 3 H, =CH), 7.50 (d, *J* = 7.6 Hz, 2 H, =CH), 7.32 (t, *J* = 7.0 Hz, 1 H, =CH), 7.13 (d, *J* = 7.6 Hz, 2 H, =CH), 5.36 (s, 2 H, -CH_2_-), 2.44 (s, 3 H, -CH_3_), 2.25 (s, 3 H, -CH_3_); ^13^C-NMR (126 MHz, DMSO-*d*_6_) δ 177.0 (-C=O), 166.1 (-C=O), 142.9 (-C=C-), 141.1 (-C=C-), 136.6 (-C=C-), 135.9 (-C=C-), 134.4 (-C=C-), 133.0 (-C=C-), 131.1 (-C=C-), 129.7 (-C=C-), 127.1 (-C=C-), 126.3 (-C=C-), 121.9 (-C=C-), 121.9 (-C=C-), 121.7 (-C=C-), 119.7 (-C=C-), 116.3 (-C=C-), 116.2 (-C=C-), 49.6 (-CH_2_-), 20.9 (-CH_3_), 20.7 (-CH_3_); FT-IR (KBr) 3263 (N-H), 3130 (=C-H), 2920 (-C-H), 1664 (-C=O), 1633 (-C=O), 1595 (-C=C-), 1547 (-C=C-), 1499 (-C=C-), 1460 (-C=C-), 1445 (-C-H), 1406 (-C-H), 1364 (-C-H), 1310 (-C-H), 1282 (-C-H), 1256 (-C-O), 1212 (-C-N), 1185 (-C-O) cm^−1^; ESI-HRMS m/z for C_23_H_21_N_2_O_2_ [M + H]^+^ calcd 357.1598, found 357.1599.

#### *N-*(4-methoxyphenyl)-2-(2-methyl-9-oxoacridin-10(9*H*)-yl)acetamide, 7g

To a solution of 2-(2-methyl-9-oxoacridin-10(9 *H*)-yl)acetic acid (20 mg, 0.075 mmol) in 0.5 mL of dry DMF, *p*-anisidine (13.8 mg, 0.112 mmol), EDCI (21.5 mg, 0.112 mmol) and 17 mg of HOBt hydrate (0.112 mmol) were successively added. The reaction mixture was stirred at room temperature for 24 h and then it was concentrated under reduced pressure. The product was purified by crystallization using a mixture of DCM/acetone/MeOH (1/0.2/0.1). The crystals were collected by filtration and washed with petroleum ether. The product was isolated as a yellow solid in 50% yield (14 mg). **7 g**: mp = 282–286 °C; ^1^H-NMR (500 MHz, CDCl_3_) δ 8.33 (d, *J* = 7.6 Hz, 1 H, =CH), 8.11 (s, 1 H, =CH), 8.00 (s, 1 H, -NH), 7.74 (t, *J* = 7.8 Hz, 1 H, =CH), 7.56 (d, *J* = 8.8 Hz, 1 H, =CH), 7.46–7.38 (m, 3 H, =CH), 7.35 (d, *J* = 8.8 Hz, 1 H, =CH), 7.25 (m, 1 H, overlapping with CDCl_3_, =CH), 6.82 (d, *J* = 8.6 Hz, 2 H, =CH), 5.07 (s, 2 H, -CH_2_-), 3.76 (s, 3 H, -OCH_3_), 2.42 (s, 3 H, -CH_3_); ^13^C-NMR (126 MHz, CDCl_3_) δ 177.8 (-C=O), 165.6 (-C=O), 157.1 (-C=C-), 142.1 (-C=C-), 140.3 (-C=C-), 136.0 (-C=C-), 134.4 (-C=C-), 132.3 (-C=C-), 129.7 (-C=C-), 128.1 (-C=C-), 127.4 (-C=C-), 122.6 (-C=C-), 122.5 (-C=C-), 122.3 (-C=C-), 114.4 (-C=C-), 114.3 (-C=C-), 114.1 (-C=C-), 55.5 (-OCH_3_), 52.1 (-CH_2_-), 20.6 (-CH_3_); FT-IR (KBr) 3246 (N-H), 3129 (=C-H), 3072 (=C-H), 2951 (-C-H), 2926 (-C-H), 2831 (-C-H), 1661(-C=O), 1635 (-C=O), 1616 (-C=C-), 1597 (-C=C-), 1551 (-C=C-), 1511 (-C=C-), 1488 (-C=C-), 1464 (-C=C-), 1414 (-C=C-), 1365 (-C-H), 1302 (-C-H), 1283 (-C-H), 1247 (-C-O), 1212 (-C-N), 1182 (-C-O) cm^−1^; ESI-HRMS m/z for C_23_H_21_N_2_O_3_ [M + H]^+^ calcd 373.1547, found 373.1543.

#### *N*-(2-methoxyphenyl)-2-(2-methyl-9-oxoacridin-10(9*H*)-yl)acetamide, 7h

To a solution of 2-(2-methyl-9-oxoacridin-10(9 *H*)-yl)acetic acid **6** (40 mg, 0.15 mmol) in 1 mL of dry DMF, *o*-anisidine (25 μL, 0.23 mmol), EDCI (43 mg, 0.225 mmol) and HOBt hydrate (35 mg, 0.225 mmol) were successively added. The reaction mixture was stirred at room temperature for 24 h and then it was concentrated under reduced pressure. The residue was purified by flash column chromatography (eluent; hexane/ethyl acetate = 2/1) to afford 37 mg of **7h** in 66% yield. **7h**: mp = 214–217 °C; ^1^H-NMR (500 MHz, CDCl_3_) *δ* 8.59 (d, *J* = 8.0 Hz, 1 H, =CH), 8.37 (s, 1 H, =CH), 8.28 (d, *J* = 8.0 Hz, 1 H, =CH), 8.13 (s, 1 H, -NH), 7.72 (t, *J* = 7.8 Hz, 1 H, =CH), 7.55 (d, *J* = 8.7 Hz, 1 H, =CH), 7.43 (d, *J* = 8.7 Hz, 1 H, =CH), 7.39–7.30 (m, 2 H, =CH), 7.01 (t, *J* = 7.8 Hz, 1 H, =CH), 6.93 (t, *J* = 7.8 Hz, 1 H, =CH), 6.70 (d, *J* = 8.0 Hz, 1 H, =CH), 5.11 (s, 2 H, -CH_2_-), 3.39 (s, 3 H, OCH_3_), 2.47 (s, 3 H, -CH_3_); ^13^C-NMR (126 MHz, CDCl_3_) δ 177.9 (-C=O), 165.1 (-C=O), 148.1 (-C=C-), 141.9 (-C=C-), 140.0 (-C=C-), 135.8 (-C=C-), 134.3 (-C=C-), 132.1 (-C=C-), 128.1 (-C=C-), 127.4 (-C=C-), 126.3 (-C=C-), 124.7 (-C=C-), 122.6 (-C=C-), 122.5 (-C=C-), 122.1 (-C=C-), 121.0 (-C=C-), 120.0 (-C=C-), 114.5 (-C=C-), 114.3 (-C=C-), 110.1 (-C=C-), 55.5 (OCH_3_), 51.5 (-CH_2_N-), 20.6 (-CH_3_); FT-IR (KBr): 3418 (N-H), 2924 (-C-H), 1688 (-C=O), 1635 (-C=O), 1597 (-C=C-), 1532 (-C=C-), 1505 (-C=C-), 1489 (-C=C-), 1460 (-C=C-), 1433 (-C=C-), 1370 (-C-H), 1326 (-C-H), 1287 (-C-H), 1250 (-C-O), 1211 (-C-N), 1181 (-C-O) cm^−1^; ESI-HRMS m/z for C_23_H_21_N_2_O_3_ [M + H]^+^ calcd 373.1547, found 373.1542.

#### Methyl 4-(2-(2-methyl-9-oxoacridin-10(9*H*)-yl)acetamido)benzoate, 7i

To a solution of 2-(2-methyl-9-oxoacridin-10(9 *H*)-yl)acetic acid (30 mg, 0.112 mmol) in 0.75 mL of dry DMF, 4-methyl 4-aminobenzoate (26 mg, 0.168 mmol), EDCI (32 mg, 0.168 mmol) and HOBt hydrate (26 mg, 0.168 mmol) were successively added. The reaction mixture was stirred at room temperature for 48 h and then it was concentrated under reduced pressure. The product was purified by crystallization using a mixture of DCM/acetone/MeOH (1/0.2/0.1). The crystals were collected by filtration, washed with DCM and petroleum ether. The product was isolated as a yellow solid in 16% yield (7 mg). **7i**: Decomposition temperature = 309 °C; ^1^H-NMR (500 MHz, DMSO-*d*_6_) δ 10.96 (s, 1 H, -NH), 8.36 (d, *J* = 7.9 Hz, 1 H, =CH), 8.16 (s, 1 H, =CH), 7.95 (d, *J* = 8.5 Hz, 2 H, =CH), 7.83–7.74 (m, 3 H, =CH), 7.70–7.59 (m, 3 H, =CH), 7.33 (t, *J* = 7.5 Hz, 1 H, =CH), 5.42 (s, 2 H, -CH_2_-), 3.82 (s, 3 H, -OCH_3_), 2.44 (s, 3 H, -CH_3_); ^13^C-NMR (126 MHz, DMSO-*d*_6_) δ 176.6 (-C=O), 166.5 (-C=O), 165.7 (-C=O), 143.0 (-C=C-), 142.4 (-C=C-), 140.6 (-C=C-), 136.0 (-C=C-), 135.5 (-C=C-), 134.0 (-C=C-), 130.8 (-C=C-), 130.4 (-C=C-), 126.6 (-C=C-), 125.8 (-C=C-), 121.5 (-C=C-), 121.4 (-C=C-), 121.3 (-C=C-), 118.7 (-C=C-), 115.8 (-C=C-), 115.7 (-C=C-), 51.9 (-OCH_3_), 49.2 (-CH_2_-), 20.2 (-CH_3_); FT-IR (KBr): 3236 (N-H), 3062 (-C-H), 2923 (-C-H), 2847 (-C-H), 1715 (-C=O), 1664 (-C=O), 1639 (-C=O), 1547 (-C=C-), 1505 (-C=C-), 1488 (-C=C-), 1467 (-C=C-), 1435 (-C=C-), 1409 (-C=C-), 1361 (-C-H), 1281 (-C-H), 1259 (-C-O), 1211 (-C-N), 1181 (-C-O) cm^−1^; ESI-HRMS m/z for C_24_H_21_N_2_O_4_ [M + H]^+^ calcd 401.1496, found 401.1489.

#### *N*-(4-acetylphenyl)-2-(2-methyl-9-oxoacridin-10(9*H*)-yl)acetamide, 7j

A solution of 2-(2-methyl-9-oxoacridin-10(9 *H*)-yl)acetic acid (30 mg, 0.112 mmol) in 0.75 mL of dry DMF, *p*-acetylaniline (23 mg, 0.168 mmol), EDCI (32 mg, 0.168 mmol) and HOBt hydrate (26 mg, 0.168 mmol) were successively added. The mixture was stirred at room temperature for 24 h, and then it was concentrated under reduced pressure. The product was crystallized using a DCM/acetone/MeOH (1/0.2/0.1). The crystals were collected by filtration and washed with petroleum ether. The product was isolated as a yellow solid in 16% yield (7 mg). **7j**: mp: 306–309 °C; ^1^H-NMR (500 MHz, DMSO-*d*_6_) δ 10.93 (s, 1 H, -NH), 8.36 (d, *J* = 8.0 Hz, 1 H, =CH), 8.16 (s, 1 H, =CH), 7.96 (d, *J* = 8.6 Hz, 2 H, =CH), 7.80 (t, *J* = 7.8 Hz, 1 H, =CH), 7.75 (d, *J* = 8.6 Hz, 2 H, =CH), 7.69–7.64 (m, 3 H, =CH), 7.34 (t, *J* = 7.5 Hz, 1 H, =CH), 5.42 (s, 2 H, -CH_2_-), 2.53 (s, 3 H, -CH_3_), 2.44 (s, 3 H, -CH_3_); ^13^C-NMR (126 MHz, DMSO-*d*_6_) δ 196.5 (-C=O), 176.5 (-C=O), 166.5 (-C=O), 142.9 (-C=C-), 142.4 (-C=C-), 140.6 (-C=C-), 135.5 (-C=C-), 134.0 (-C=C-), 132.0 (-C=C-), 130.8 (-C=C-), 129.6 (-C=C-), 126.6 (-C=C-), 125.8 (-C=C-), 121.5 (-C=C-), 121.3 (-C=C-), 118.5 (-C=C-), 115.8 (-C=C-), 115.7 (1 C missing due to overlapping, (-C=C-), 49.2 (-CH_2_N-), 26.4 (-CH_3_), 20.2 (-CH_3_); FT-IR (KBr): 3291 (N-H), 1678 (-C=O), 1634 (-C=O), 1613 (-C=O), 1595 (-C=C-), 1528 (-C=C-), 1504 (-C=C-), 1489 (-C=C-), 1468 (-C=C-), 1404 (-C=C-), 1367 (-C=C-), 1318 (-C-H), 1269 (-C-H), 1213 (-C-H), 1183 (-C-O), 1120 (-C-N) cm^−1^; ESI-HRMS m/z for C_24_H_21_N_2_O_3_ [M + H]^+^ calcd 385.1547, found 385.1545.

### Expression and purification of MARK4

The human *MARK4* was cloned, expressed and purified by following our previously reported protocols^38,39^. In brief, the recombinant cells harbouring the expression construct of MARK4 were grown in Luria-Bertani broth and culture were induced by IPTG (1 mM) followed by overnight culture of cells at 16 °C with continuous vigorous shaking. The pellet was obtained by centrifuging the culture, dissolved in lysis buffer (50 mM Tris, 20 mM EDTA, 0.1 mM PMSF and 1% Triton-100) and inclusion bodies were prepared. Definite amount of inclusion bodies were taken and dissolved in sarcosine buffer (50 mM CAPS, 1.5% N-laurosyl sarcosine, pH 11.0) and were centrifuged for 25 min at 12,000 rpm and the supernatant was allowed to bind on preequlibrated Ni-NTA column GE healthcare (GE Healthcare Life Sciences, Uppsala, Sweden). After washing (with 5 mM imidazole in sarcosine buffer), protein was eluted with increasing concentration of imidazole (10 mM to 400 mM). Fractions containing MARK4 protein were pooled down and further purity was accessed using sodium dodecyl sulphate polyacrylamide gel electrophoresis (SDS-PAGE) and confirmed with the help of Western blot using peptide specific primary antibodies^40^.

### Kinase inhibition assay

Enzyme activity of MARK4 was evaluated with the help of standard Malachite Green (Biomol Green reagent, Enzo Life Sciences) microtitre-plate assay as described earlier^41^. Estimated amount of MARK4 (200 ng) and 20 µM ATP as a substrate were incubated for 10–15 min at 25 °C with increasing concentrations (0–20 µM) of synthesized acridone derivatives, in 20 mM Tris buffer, pH 8.0. Reaction was terminated by the adding 100 µl of Biomol Green reagent and absorbance of final reaction products was recorded at 620 nm in a multiplate ELSIA reader (BioRad). In order to quantify the hydrolyzed phosphate, inorganic phosphate standard curve were prepared from the phosphate standard solutions as supplied by the manufacturer (Biomol Green reagent, Enzo Life Sciences). All data points represent triplicate measurements from at least three independent experiments.

### Molecular docking

Autodock Vina and AutoDock 4 package was used for molecular docking^34^. Autodock Vina uses an advanced docking algorithm and scoring function of protein ligand interactions. Atomic coordinates of MARK4 was retrieved from RCSB Protein Data Bank (www.rcsb.org) using the PDB ID 5ES1^42^. Prior to docking analysis, the structure was emended by removing water and co-crystallized ligand , followed by addition of polar hydrogens and Gasteiger charges using Auto Dock Tool (ADT). The 2D and 3D structures of all the synthesized compounds **5**, **7a-7j** were generated and energy minimized by ChemBio3D Ultra 12.0. Both ligands and receptor were transformed to the proper format for docking through PyRx. Following the standard docking procedure, ligands were docked by defining a grid box with spacing 1 Å and size of 20 × 20 × 20 (Å) pointing in x, y and z directions around the protein active site. After preparing the coordinate files of MARK4 and respective compounds, they were subjected to molecular docking in order to see the bound conformations, binding affinity and possible protein-ligand interactions. The “exhaustiveness” was set to the value of 100 instead of the default 8 for all docking analysis. PyMOL viewer (Schrödinger, LLC) and “Receptor-Ligand Interactions” modules of BIOVIA/Discovery Studio 2017R2 were used for the visualization and structure analysis of the docked complexes of MARK4 and to generate two dimensional docking for the analysis of hydrogen bonds and hydrophobic interactions^43^.

### Fluorescence measurements

Binding affinities of synthesized acridone derivatives with recombinant MARK4 was carried out by observing the fluorescence intensity change of emission spectrum of MARK4 by following our previously published protocol^44,45^. Each titration of protein was performed in triplicates and the average was taken for analysis. A significant decreased in fluorescence intensity of protein with increase in the concentration of acridone derivatives were used as the criteria for deducing the binding constant (*K*_a_) as well as number of binding sites (*n*) present on the protein molecule using the modified Stern-Volmer equation^46^:

1 $$\mathrm{log}({{\rm{F}}}_{{\rm{o}}}-{\rm{F}})/{\rm{F}}=\,\mathrm{log}\,{{\rm{K}}}_{{\rm{a}}}+{\rm{nlog}}[{\rm{L}}]$$

where, F_o_ = Fluorescence intensity of native protein, F = Fluorescence intensity of protein in the presence of ligand, *K*_a_ = Binding constant, *n* = number of binding sites, L = concentration of ligand. The values for *K*_a_ and *n* were derived from the intercept and slope, respectively.

### Isothermal titration calorimetry

ITC experiments were performed on a VP-ITC microcalorimeter (MicroCal, Inc. GE, MicroCal, USA) by following our previously published protocols^41,47^. For sample preparation, recombinant MARK4 was extensively dialyzed in 20 mM Tris buffer, pH 8.5 and the working solutions of acridone derivatives were prepared in last dialyzing buffer. The titration data so obtained was analyzed with the help of MicroCal Origin 7.0 software provided with the instrument. The thermodynamic parameters of binding such as association constant (*K*_a_), stoichiometry of binding (*n*), and enthalpy change (Δ*H*) were determined by fitting the binding isotherm into the ‘one-set of sites’ binding model.

### Cell culture

HEK-293 and MCF-7 human cell lines were grown and maintained in a DMEM supplemented with 10% heat-inactivated fetal bovine serum (Gibco) and 1% penicillin, streptomycin solution (Gibco), in a 5% CO_2_ humidified incubator at 37 °C. Routinely cells were cultured, maintained and trypsinized not more than 30 passages.

### Cell viability assay

To study the effect of synthesized acridone derivatives on cell viability and proliferation, a standard MTT method was carried out. Cells were plated (8000–9000/well) in a 96-well plate and incubated overnight. On the next day, cells were treated with increasing concentrations (0.1–80 μM) of synthesized acridone derivatives in a final volume of 200 µl for 48 h at 37 °C in a humidified chamber. At the end of treatment time point, 20 µl of MTT solution (from 5 mg/ml stock solution in phosphate buffer saline, pH 7.4) was added to each well and incubated further for 4–5 h at 37 °C in the CO_2_ incubator. After stipulated time the supernatant was aspirated and the colored formazan crystal produced as a result of MTT reduction was dissolved in 100 µl of DMSO. The absorbance (A) of colored product was then measured at 570 nm on a multiplate ELISA reader (BioRad). The percentage of viable cells was calculated and used to estimate the IC_50_ (50% inhibitory concentration) values for each acridone derivative. For cell proliferation studies paclitaxel has been taken as positive control.

### Cell apoptosis assay

Annexin-V staining was used to analyse the apoptotic potential of synthesized acridone derivatives as described previously^47,48^. Briefly, MCF-7 cells were dosed with IC_50_ concentration of selected acridone derivative for 24 h at 37 °C. The control cells were treated with media only. After 24 h treatment, nearly 2.0–2.5 × 10^6^ cells were trypsinized and collected by centrifuging the cell suspension at 1800 rpm for 4 min. Collected cells were washed two times with 5 ml of PBS. Finally, cells were stained with FITC labeled Annexin-V antibodies using FITC-Annexin-V kit according to the manufacturer’s guidelines (BD-Biosciences, USA). Approximately, 10,000 events were analyzed for each sample through flow cytometry on BD LSR II Flow Cytometry Analyzer and FlowJo.

### Reactive oxygen species measurement

DCF fluorescence helps to measures innumerable ROS such as H_2_O_2_ and hydroxyl radicals^49^. That why DCFDA staining was used to quantify the reactive oxygen species (ROS) level inside the cell as described earlier^17,41^. Briefly, the MCF-7 cells (70–80% confluent) were incubated with IC_50_ concentration of each compound and positive control H_2_O_2,_ respectively in a 24-well culture plates. After 5–6 h incubation of cells with respective compounds, cells were gently washed with 500 μl of prewarmed (at 37 °C) Krebs Ringer buffer (20 mM HEPES, 2 mM MgSO_4_, 10 mM dextrose, 127 mM NaCl, 1 mM CaCl_2_ and 5.5 mM KCl), subsequently 10 µM DCFDA (Invitrogen Grand Island, NY) has been added to each well and incubated further for 30 min in dark at 37 °C in a humidified CO_2_ incubator. After 30 min incubation, the cells were collected by trypsinization and ROS levels were quantified with the help of Flow Cytometry. Nearly 10,000 events for each sample were collected on BD LSR II Flow Cytometry Analyzer, and analyzed with help of FlowJo. For intact cell imaging, cells were washed with PBS, pH 7.4 and imaged for ROS levels estimation using the DCFDA fluorescent dye. Fluorescence images were taken on Nikon-EclipseTS100 microscope.

### Single Dose Kinase Inhibition Profiling

*In vitro* profiling of the twenty five members of CAMK family (CAMK-1 and CAMK-2) of kinases was performed with the help of kinase screening kit by following the manufacturer’s protocols (Promega, Madison, USA). Briefly, add desired concentration of each compound (10 µM) to corresponding well of 384-well assay plate. Consecutively, 2 µl of kinase working stock solution was added to each wells of assay plate. Gently mix the reaction mixture on a plate shaker for 2 minutes, centrifuge the assay plate to bring mixture of reagents to the bottom of the wells. Incubate the plate at room temperature (25 °C) for 10 minutes. Transfer 2 µl of working stocks of ATP/substrate each well, making sure to use the appropriate substrate for each kinase. Mix the assay plate, centrifuge and incubate at room temperature (25 °C) for 60 minutes. After 60 minute incubation 5 µl of ADP-Glo™ Reagent was added to all reactions in the assay plate. Mix the plate for 2 minutes, and incubated at room temperature for 40 minutes. Finally, 10 µl of Kinase Detection Reagent was added to each assay wells and incubate the plate at room temperature for 30 minutes. After the completion of reaction, measure the luminescence using an integration time of 0.5 seconds per well. Using net luminescence of the no-compound control (negative control) reactions to represent 100% kinase activity, the percent kinase activity was calculated in the compound-containing reactions and plotted in terms of percent kinase activity inhibition.

### Tau-phosphorylation assay

To study the effect of selected lead molecules on the phosphorylation of tau protein (a substrate of MARK4) the SH-SY5Y cells were treated with IC_50_ dose of compound 7b, 7d and 7 f for 24 h and subjected to FACS based analysis as per our previously published protocols^17,50^.

### Statistical analysis

Data were expressed as mean ± standard error from three independent experiments. The statistical analysis of each data was performed using the two-tailed Student t-test for unpaired samples and value of P < 0.05 were considered as significant.

## Supplementary information

Supplementary_Information
