# Supplementary material for: Probing the Inhibition of Microtubule Affinity Regulating Kinase 4 by N-Substituted Acridones
Source: Sci Rep. 2019 Feb 8;9:1676. doi: 10.1038/s41598-018-38217-8 (PMC6368574; doi:10.1038/s41598-018-38217-8)
Supplement: Supplementary file 1 — Supplementary_Information [file 41598_2018_38217_MOESM1_ESM.pdf]

Probing the Inhibition of Microtubule Affinity Regulating Kinase 4 by *N*-Substituted Acridones

Maria Voura[1,#], Parvez Khan[2,#], Savvas Thysiadis[1], Sotiris Katsamakas[3], Aarfa Queen[2,4], Gulam Mustafa Hasan[5], Sher Ali[2], Vasiliki Sarli[*,1] , Md. Imtaiyaz Hassan [*,2]

[#]*=these authors contributed equally to this work.*

[1]*Department of Chemistry, Aristotle University of Thessaloniki, University Campus, 54124, Thessaloniki, Greece, sarli@chem.auth.gr*
[2]*Centre for interdisciplinary research in Basic Sciences, Jamia Millia Islamia, Jamia Nagar, New Delhi-110025, India, mihassan@jmi.ac.in*
[3]*Department of Pharmaceutical Chemistry, School of Pharmacy, Aristotle University of Thessaloniki, University Campus, 54124, Thessaloniki, Greece*
[4]*Department of Chemistry, Jamia Millia Islamia, Jamia Nagar, New Delhi-110025, India*
[5]*Department of Biochemistry, College of Medicine, Prince Sattam Bin Abdulaziz University, P.O. Box 173, Al-Kharj – 11942, KSA.*

Running head: Acridones as MARK4 Inhibitors

***Correspondence**

Dr. V. Sarli
Department of Chemistry,
Aristotle University of Thessaloniki,
University Campus, 54124, Thessaloniki, Greece,
E-mail: sarli@chem.auth.gr

Dr. Md. Imtaiyaz Hassan
Centre for Interdisciplinary Research in Basic Sciences,
Jamia Millia Islamia, New Delhi-110025, India
Tel.: +91-9990323217
E-mail: mihassan@jmi.ac.in

Table S1: Molecular docking results showing the binding energy and specific interacting residues of MARK4 with the synthesized acridone derivatives

Compound	Docking score (Kcal/mol)	Protein-ligand interactions		
		Hydrogen bonds		Other interacting residues
		Amino acid residues	Distance (Å)	
**5**	-8.0	Ala135	3.20	Leu185, Ile62, Gly138, Glu139, Gly63, Asp196 Val70, Ala195, Val116, Ala83, Met132 Glu133 Tyr134, Ala135
**7a**	-9.4	Ala135	3.14	Leu185, Gly138, Ile62, Gly63, Glu139, Lys64 Gly65, Glu182, Asp196, Ala195, Lys85, Val70 Met132, Glu133, Ala83, Val116, Tyr134 Ala135
**7b**	-9.8	Ala135	3.27	Leu185, Ile62, Gly138, Glu139, Gly63, Lys64 Ala68, Lys69, Gly65, Asp196, Lys85, Val70 Glu182, Ala195, Met132, Ala83, Val116 Glu133, Tyr134, Ala135
**7c**	-9.2	Ala135	3.18	Ala83, Glu133, Val116, Met132, Gly63, Val70 Ala195, Ala68, Lys64, Lys85, Lys69, Gly65 Asp196, Asn183, Glu182, Ile62, Gly138 Leu185, Tyr134, Ala135
**7d**	-9.2	Ala135	3.26	Leu185, Ile62, Gly138, Glu139, Gly63, Lys64 Lys69, Gly65, Lys85, Asp196, Ala68, Glu182 Val70, Val116, Ala195, Ala83, Met132 GLu133, Tyr134, Ala135
**7e**	-9.3	Ala135	3.21	Leu185, Ile62, Gly138, Glu139, Gly63, Lys64 Ala68, Lys69, Gly65, Asp196, Lys85, Val70 Met132, Glu133, Ala83, Ala195, Val116 Tyr134, Ala135
**7f**	-9.5	Ala135	3.25	Ala83, Glu133, Ala195, Val116, Val70, Gly63 Met132, Asp196, Ala68, Lys85, Gly65, Ile62, Gly138, Leu185, Tyr134, Ala135
**7g**	-9.3	Ala135	3.19	Ala83, Glu133, Met132, Val116, Ala195 Val70, Gly63, Lys85, Gly65, Lys64, Asn66 Ala68, Asp196, Gly138, Ile62, Leu185, Tyr134 Ala135
**7h**	-9.5	Ala135	3.06	Leu185, Ile62, Gly138, Glu139, Gly63, Lys64 Ala68, Lys69, Gly65, Lys85, Asp196, Val70 Met132, Ala83, Glu133, Val116, Ala195 Tyr134, Ala135
**7i**	-9.3	Ala135	3.29	Leu185, Ala83, Glu133, Val116, Val70, Ala195 Met132, Gly63, Gly65, Ala68, Lys85, Asp196 Asn183, Gly138, Ile62, Tyr134, Ala135
**7j**	-9.4	Ala135	3.33	Ala83, Val70, Glu133, Val116, Met132 Ala195, Gly63, Lys85, Ala68, Gly65, Asp196 Asn183, Gly138, Ile62, Leu185, Tyr134 Ala135

Table S2: Functional activity concentrations of synthesized acridone derivatives obtained MTT assays.

Compound Name	IC_{50} (µM)		
	**HepG2**	**MCF-7**	**HEK293**
**5**	**16.4**	**9.4**	
**7a**	**24.3**	**12.3**	
**7g**	**21.2**	**14.3**	
**7d**	**15.1**	**6.3**	
**7i**	**25.1**	**14.1**	
**7c**	**19.4**	**11.9**	**>200**
**7f**	**15.2**	**5.8**	
**7b**	**13.6**	**5.2**	
**7e**	**23.8**	**13.8**	
**7h**	**19.0**	**7.2**	
**7j**	**24.4**	**16.3**	

A) Compound 7a

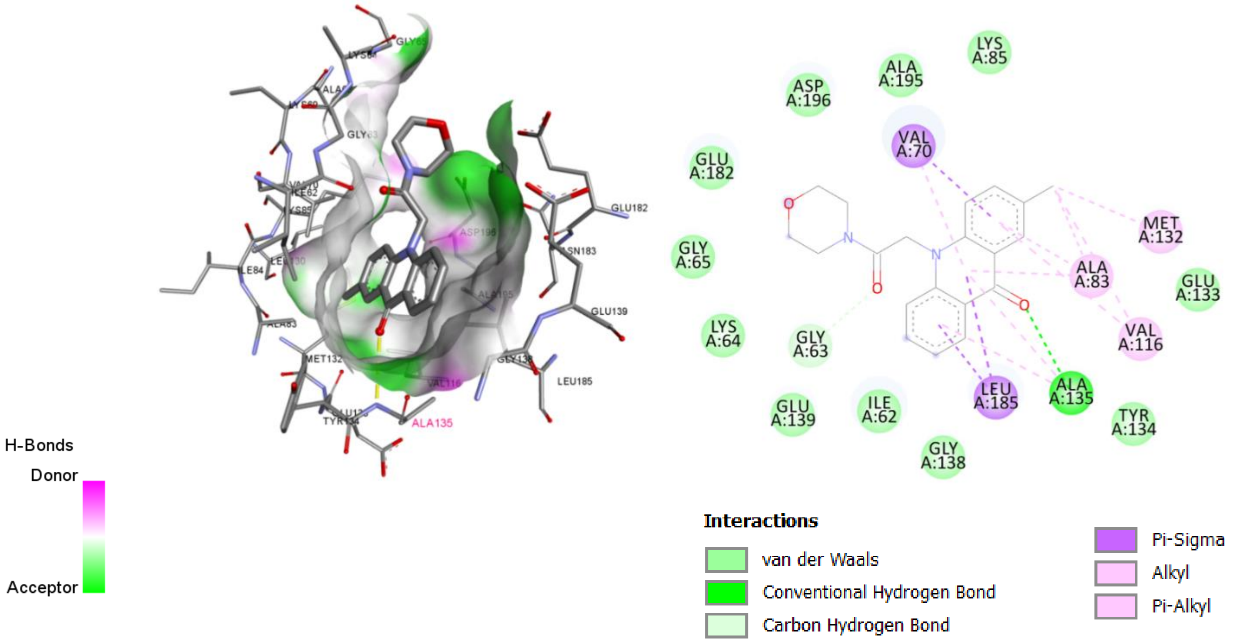

B) Compound 7c

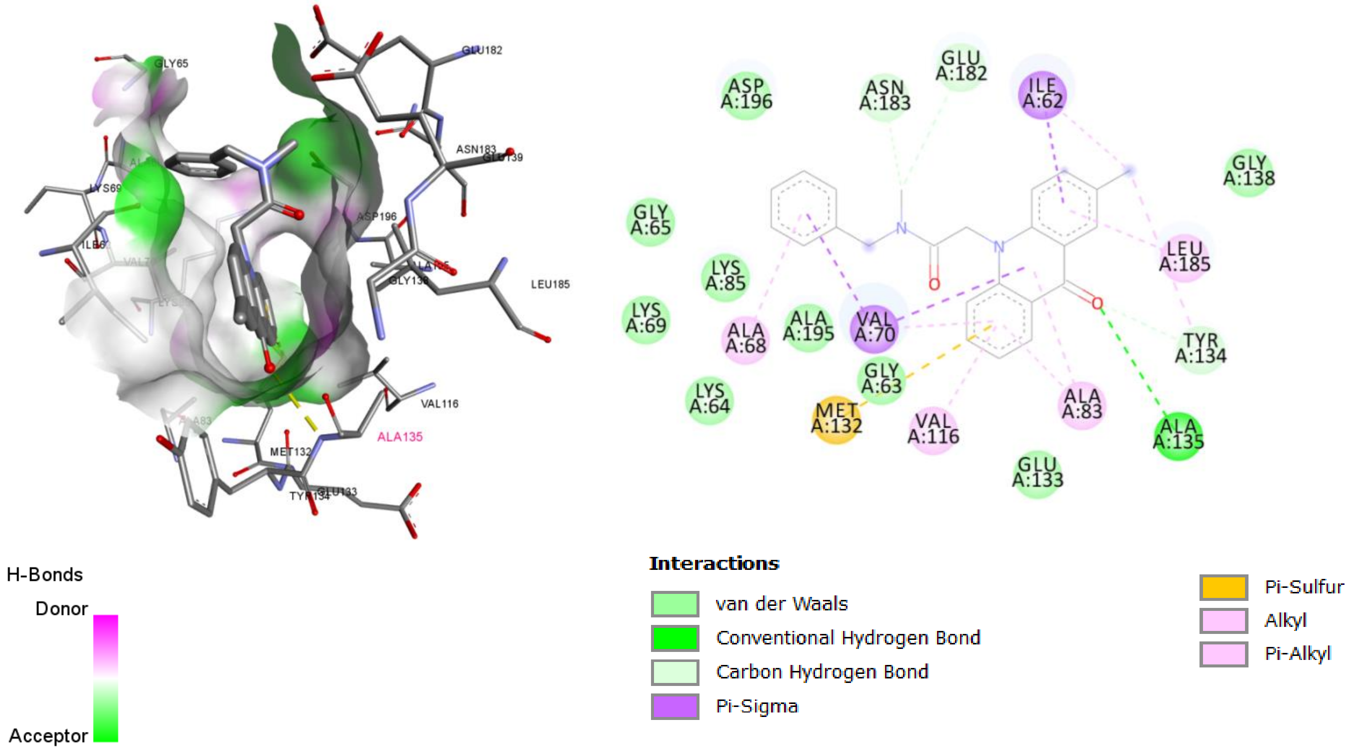

Figure S1. Molecular docking studies of selected compounds with MARK4: View of the catalytic pocket of MARK4 with (A) compound **7a**, (B) compound **7c**, and 2D schematic representation of the docking models. Dotted lines in different colors reflected various types of interaction such as hydrogen bonding, charge or polar interactions, van der Waals and π-sigma interactions.

A) Compound 7e

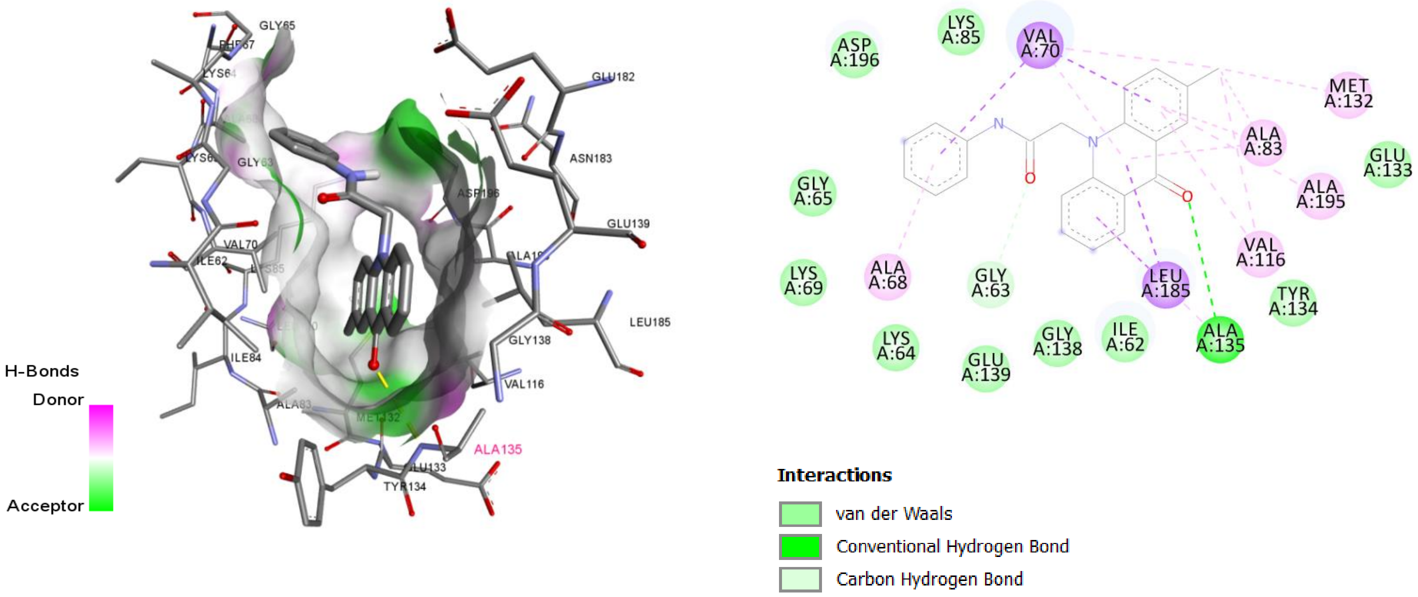

B) Compound 7g

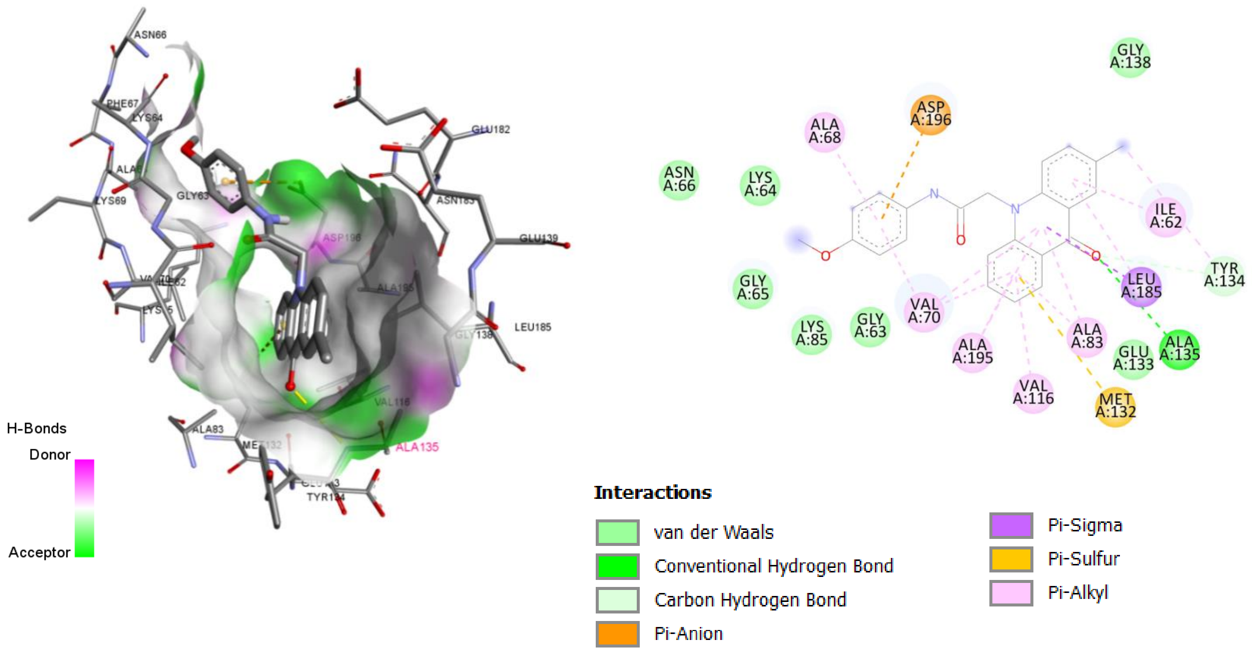

Figure S2. Molecular docking studies of selected compounds with MARK4: View of the catalytic pocket of MARK4 with (A) compound **7e**, (B) compound **7g**, and 2D schematic representation of the docking models. Dotted lines in different colors reflected various types of interaction such as hydrogen bonding, charge or polar interactions, van der Waals and π-sigma interactions.

A) Compound 7i

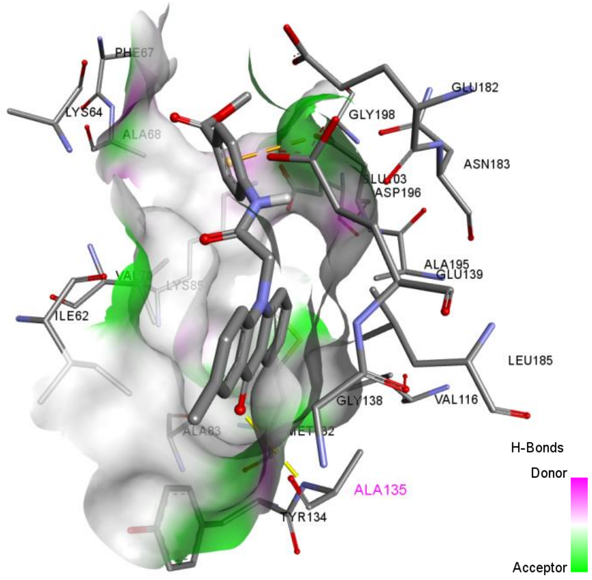

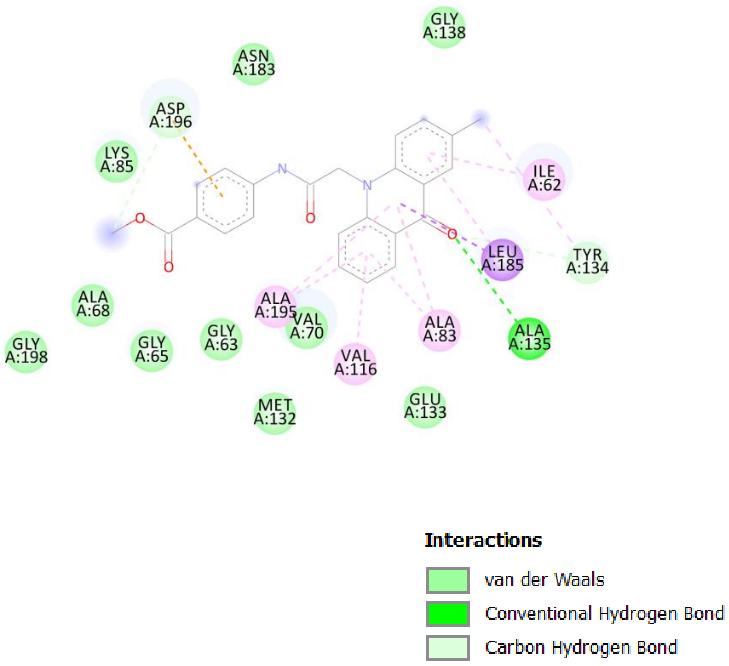

B) Compound 7j

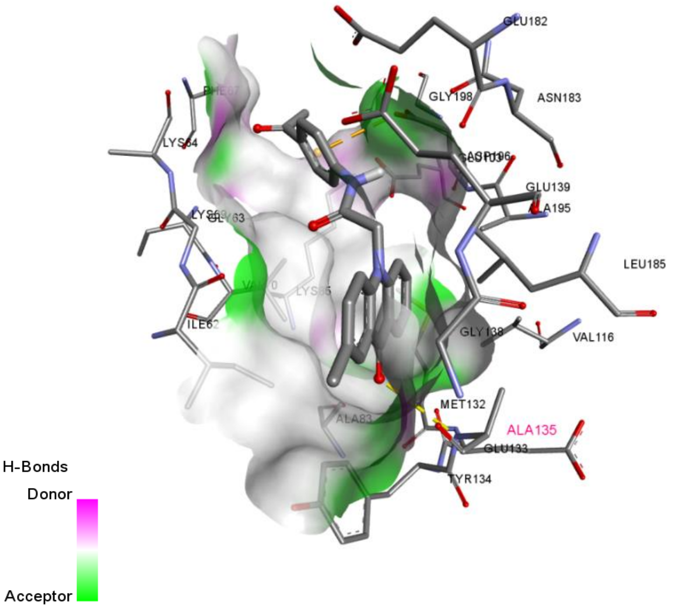

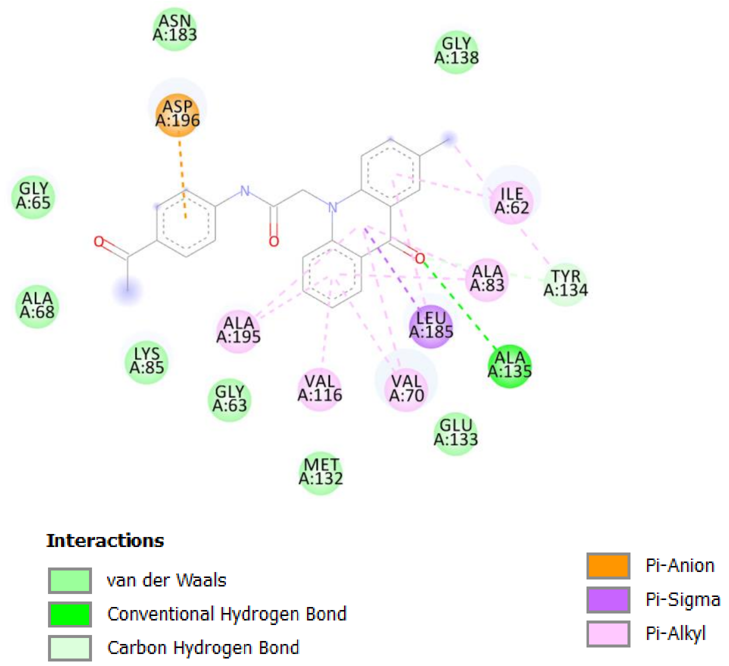

Figure S3. Molecular docking studies of selected compounds with MARK4: View of the catalytic pocket of MARK4 with (A) compound **7i**, (B) compound **7j**, and 2D schematic representation of the docking models. Dotted lines in different colors reflected various types of interaction such as hydrogen bonding, charge or polar interactions, van der Waals and π-sigma interactions.

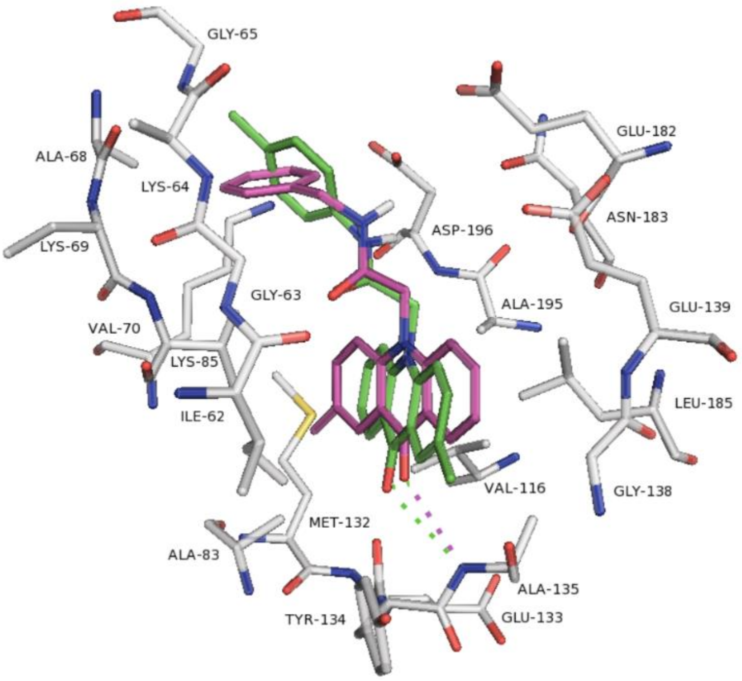

Figure S4. The two major binding modes of docked **7b** and **7f** into the active site of MARK4 (grey sticks). Both compounds form one hydrogen bond with Ala135. Compound **7b** (purple): the acridone methyl group extend towards Ala83, Val116 and Met132; Compound **7f** (green): the methyl group displays interactions with Ile62 and Tyr134.

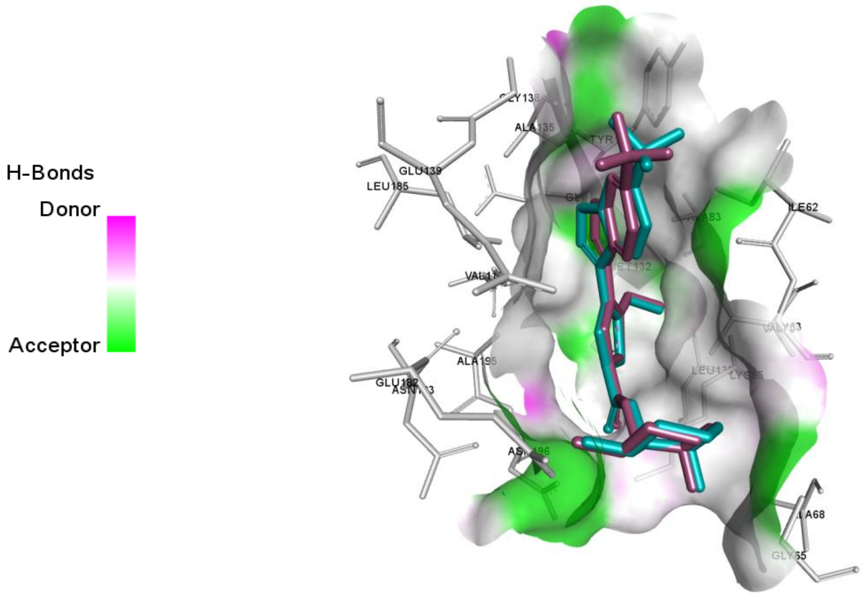

Figure S5. Docking the pyrazolopyrimidine inhibitor 5RC into the active site of the MARK4 active site (silver sticks) Compound **5RC** from the crystal structure (purple), Compound **5RC** from the docking studies (blue).

Compound 7a:

A

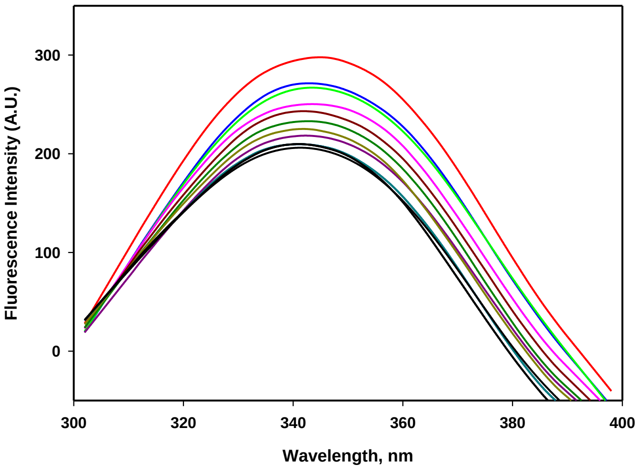

B

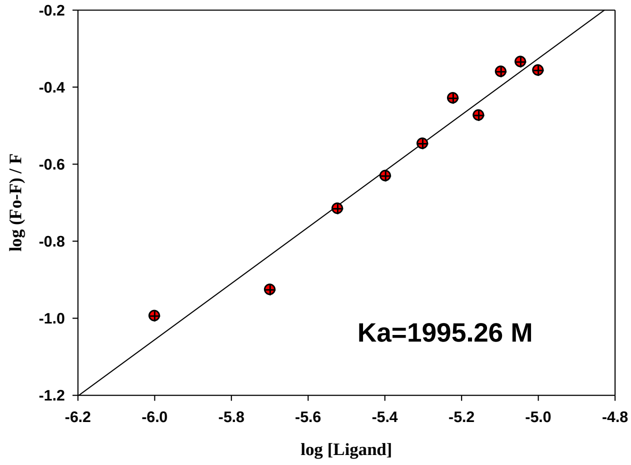

Compound 7g:

A

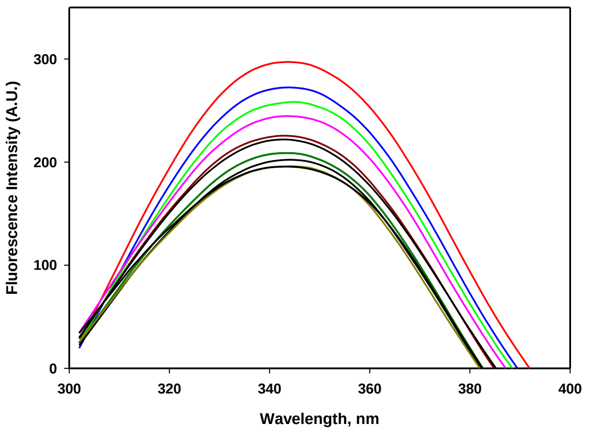

B

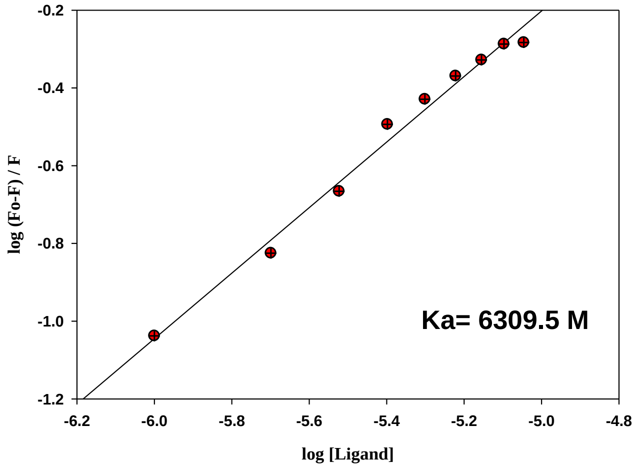

Compound 7i :

A

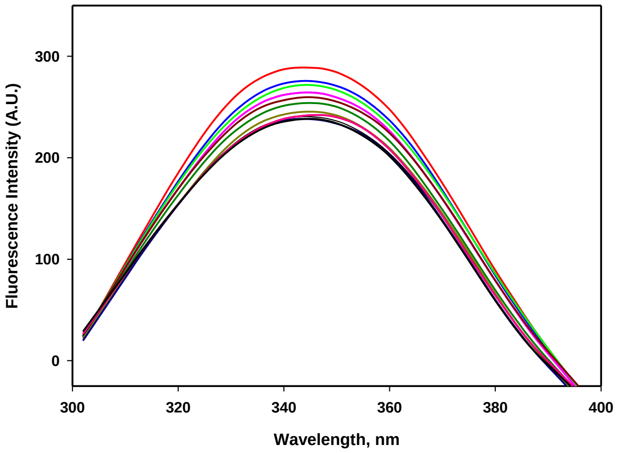

B

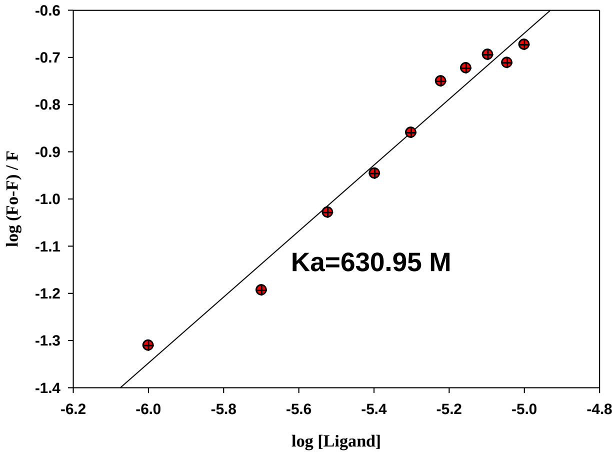

Figure S6. Fluorescence binding studies of compound 7a, 7g, and 7i with MARK4

Compound 7c:

A

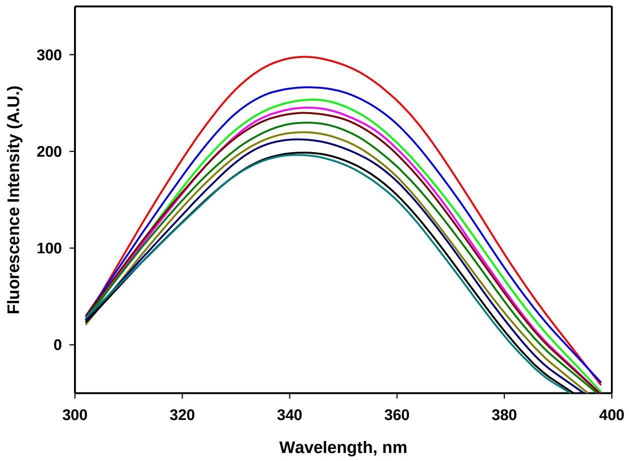

B

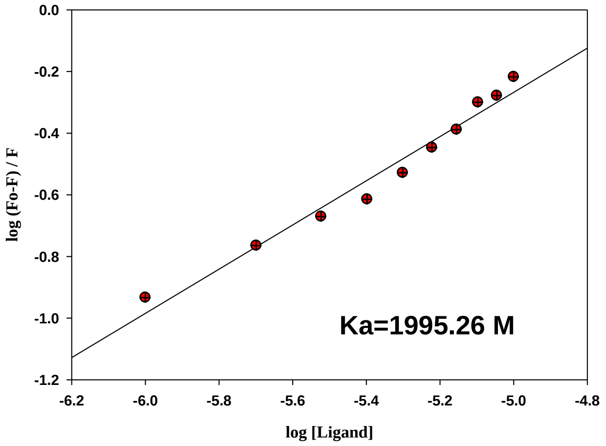

Compound 7e:

A

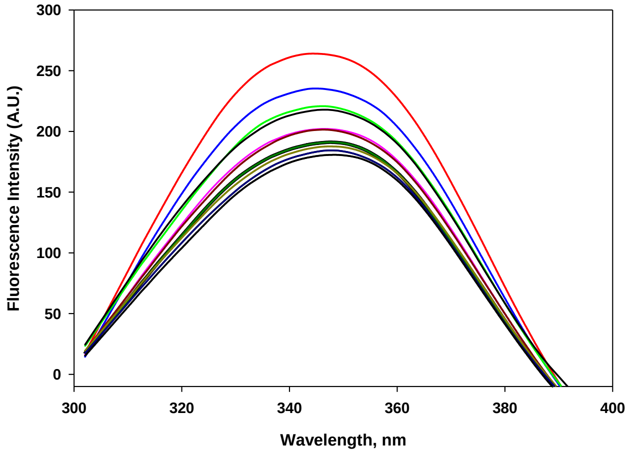

B

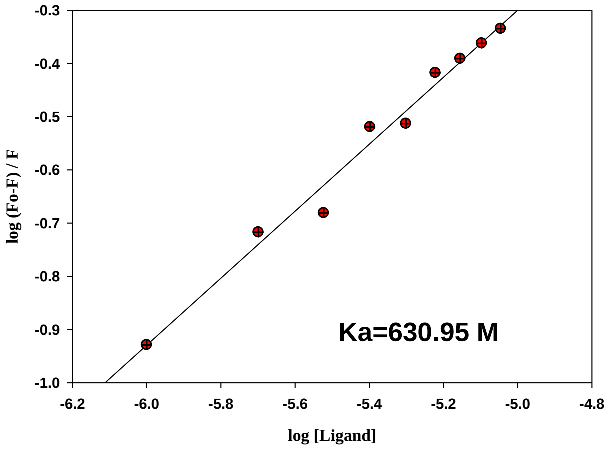

Compound 11:

A

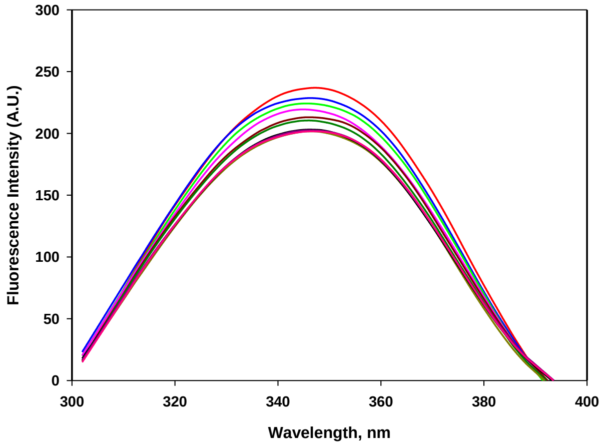

B

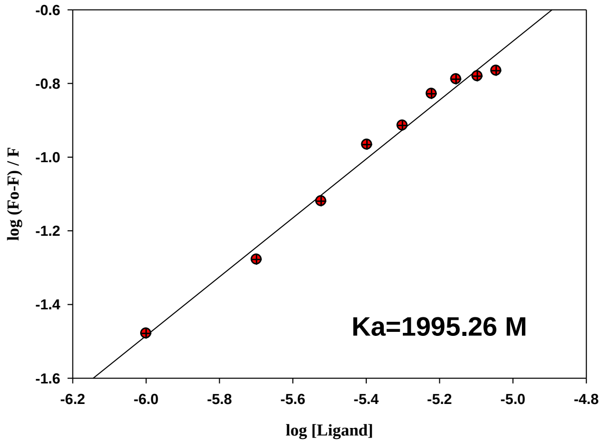

Figure S7. Fluorescence binding studies of compound 7c, 7e, and 11 with MARK4

Table S3: Structures of synthesized acridone derivatives and their enzyme inhibition IC_{50} values and binding affinity to MARK4.

Compound No.	Structure	IC_{50}, (μM), for MARK4 Inhibition	Binding affinity (Ka), M^{-1}	Compound No.	Structure	IC_{50}, (μM), for MARK4 Inhibition	Binding affinity (Ka), M^{-1}
**5**	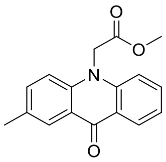	**14.12 ± 1.02**	**6.3 x 10^4**	**7c**	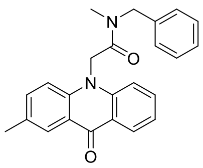	**> 20.0**	**1995.26**
**7a**	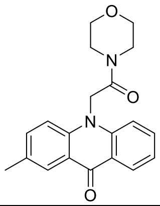	**> 20.0**	**1995.26**	**7f**	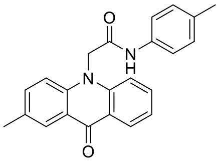	**4.5 ± 0.52**	**1.9 x 10^6**
**7g**	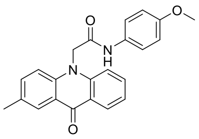	**> 20.0**	**6309.5**	**7b**	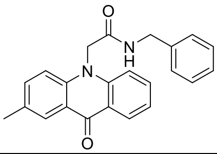	**1.80 ± 0.04**	**1.0 x 10^6**
				**7e**	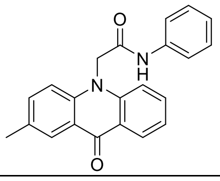	**> 20.0**	**630.95**
**7d**	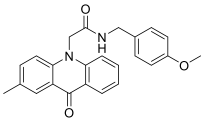	**2.20 ± 0.05**	**1.0 x 10^6**	**7h**	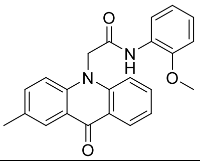	**12.15± 1.10**	**3.1 x 10^5**
**7i**	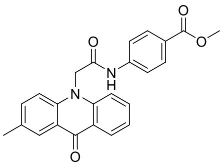	**> 20.0**	**630.95**	**7j**	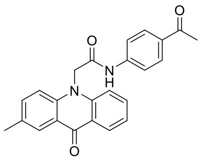	**> 20.0**	**1995.26**

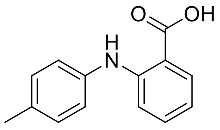

3

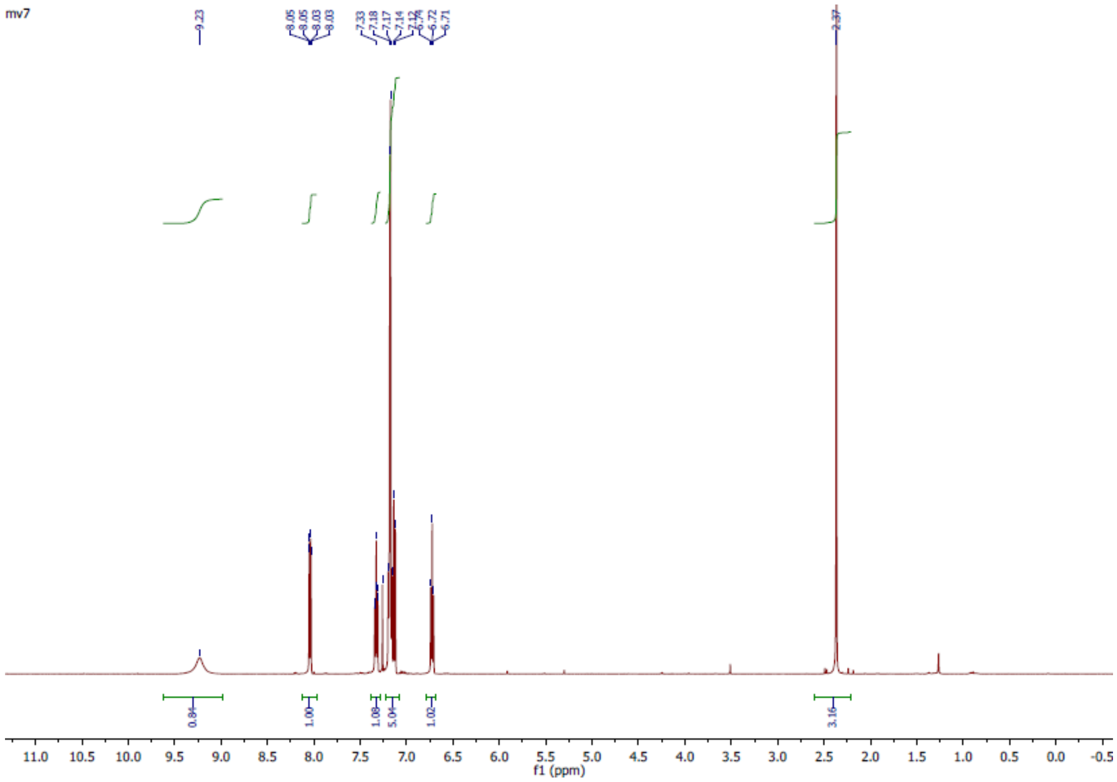

mv7
9.23
8.05
8.05
8.03
8.03
7.33
7.18
7.17
7.14
7.12
6.74
6.72
6.71
2.37
0.84
1.00
1.08
5.04
1.02
3.16
f1 (ppm)

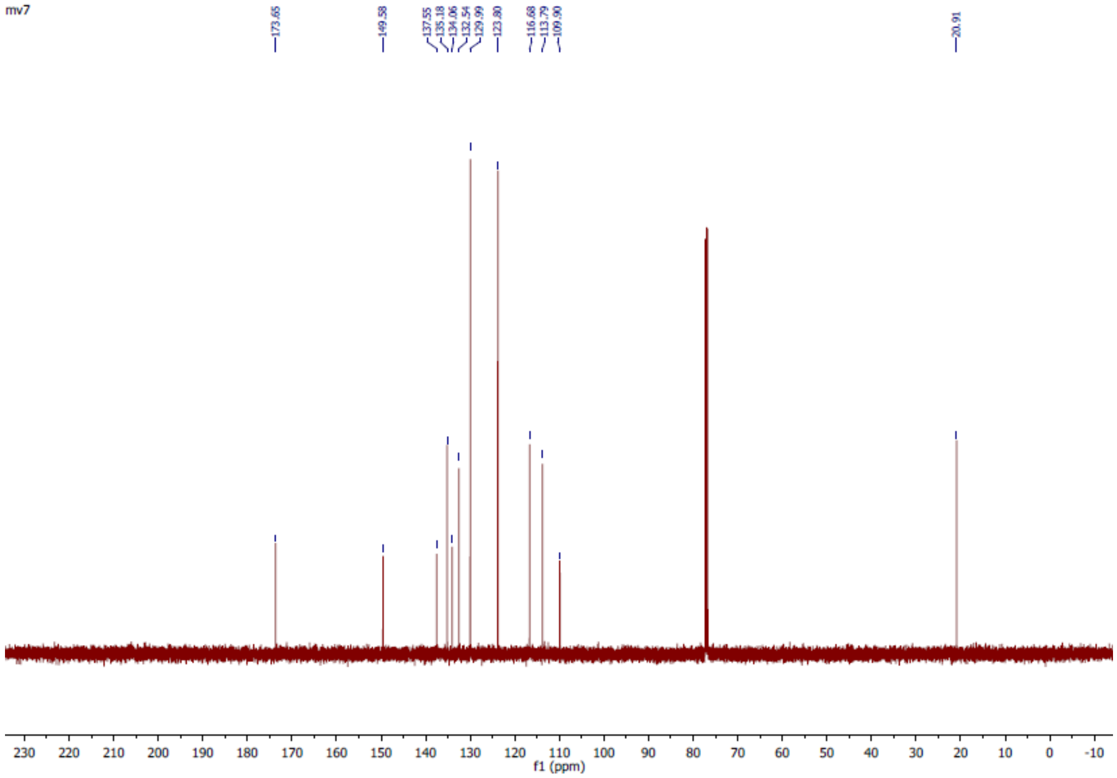

mv7
173.65
149.58
137.55
135.18
134.06
132.54
129.99
123.80
116.68
113.79
109.90
20.91
f1 (ppm)

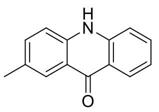
H
N
O

4

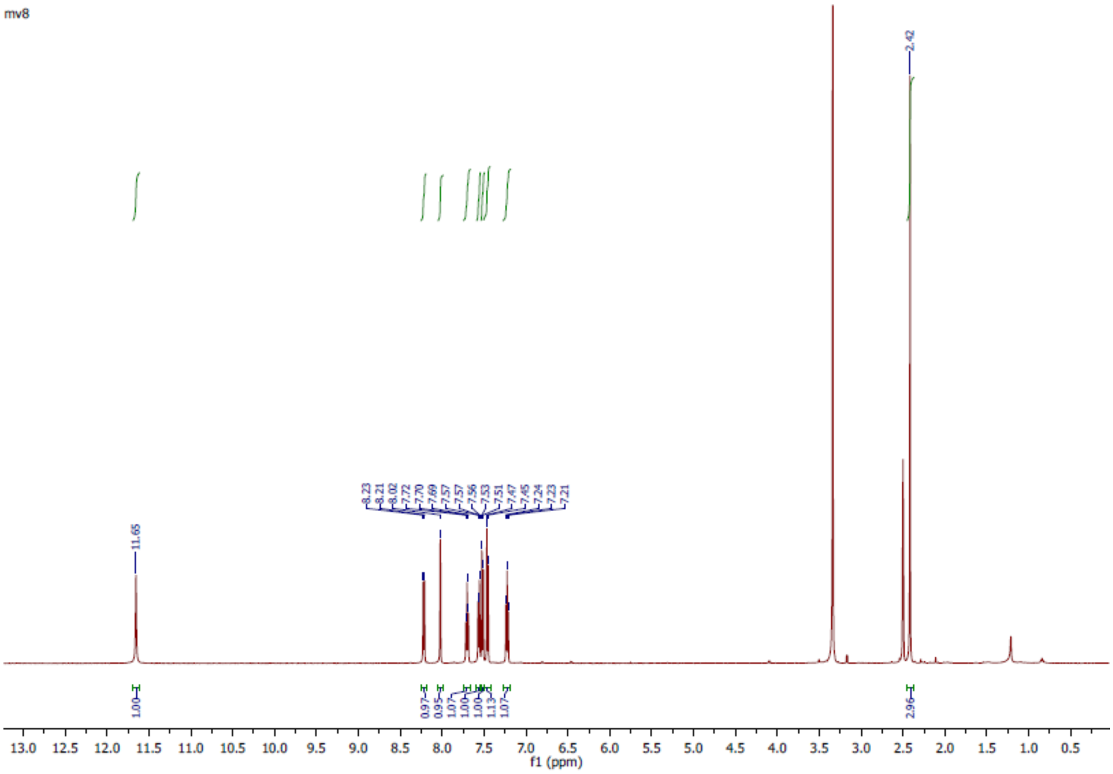
mv8
2.42
11.65
8.23
8.21
8.02
7.72
7.70
7.69
7.57
7.57
7.56
7.53
7.51
7.47
7.45
7.24
7.23
7.21
1.00
0.97
0.95
1.07
1.00
1.00
1.13
1.07
2.96
13.0 12.5 12.0 11.5 11.0 10.5 10.0 9.5 9.0 8.5 8.0 7.5 7.0 6.5 6.0 5.5 5.0 4.5 4.0 3.5 3.0 2.5 2.0 1.5 1.0 0.5
f1 (ppm)

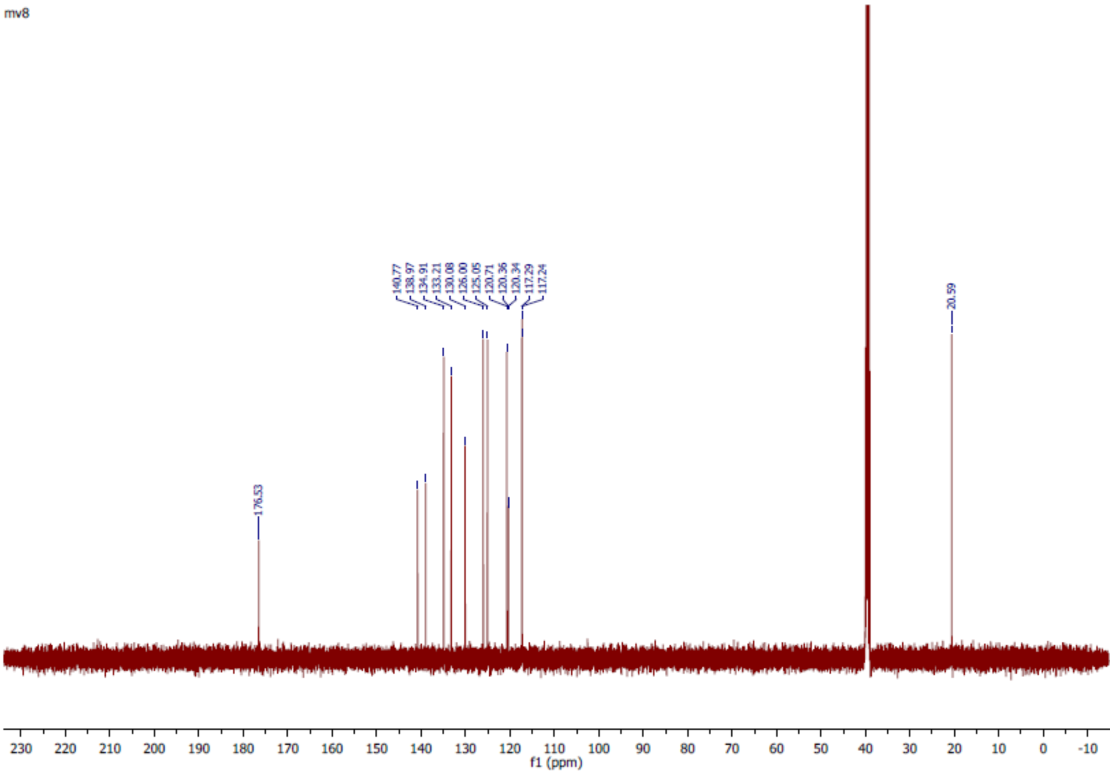
mv8
140.77
138.97
134.91
133.21
130.08
126.00
125.05
120.71
120.36
120.34
117.29
117.24
20.59
176.53
230 220 210 200 190 180 170 160 150 140 130 120 110 100 90 80 70 60 50 40 30 20 10 0 -10
f1 (ppm)

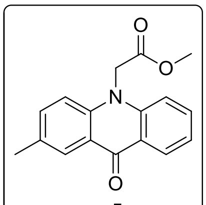

5

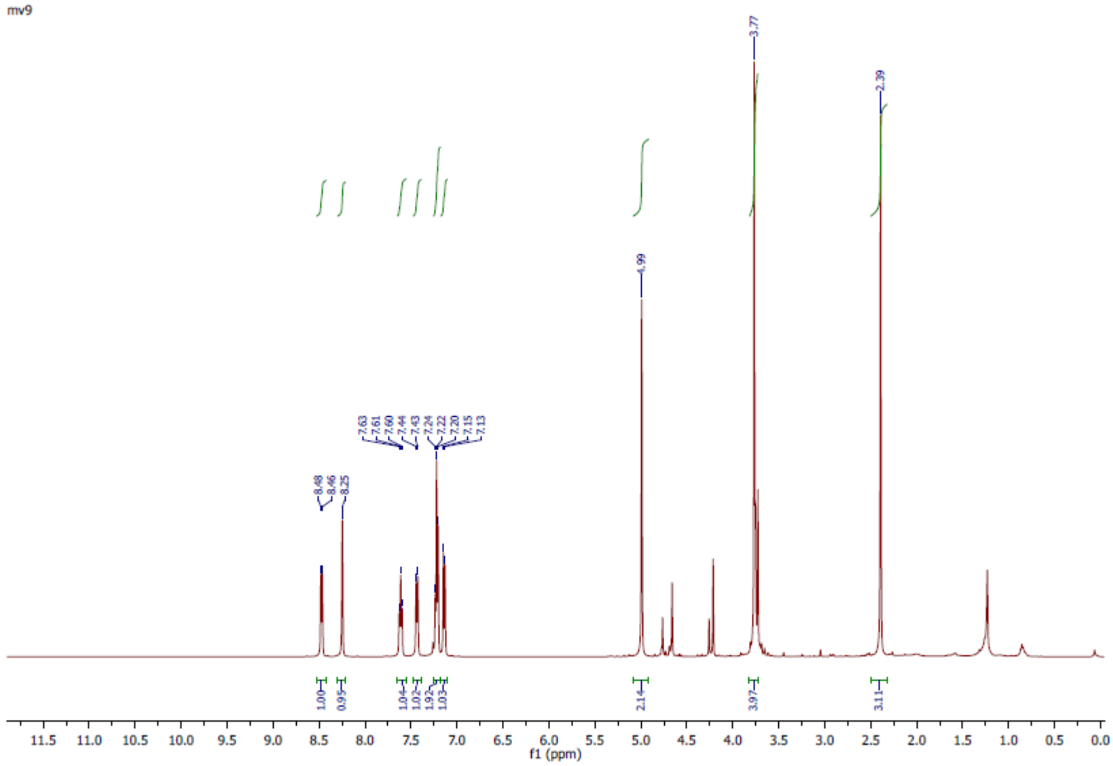
mv9
3.77
2.39
4.99
7.63
7.61
7.60
7.44
7.43
7.24
7.22
7.20
7.15
7.13
8.48
8.46
8.25
1.00
0.95
1.04
1.02
1.92
1.03
2.14
3.97
3.11
11.5 11.0 10.5 10.0 9.5 9.0 8.5 8.0 7.5 7.0 6.5 6.0 5.5 5.0 4.5 4.0 3.5 3.0 2.5 2.0 1.5 1.0 0.5 0.0
f1 (ppm)

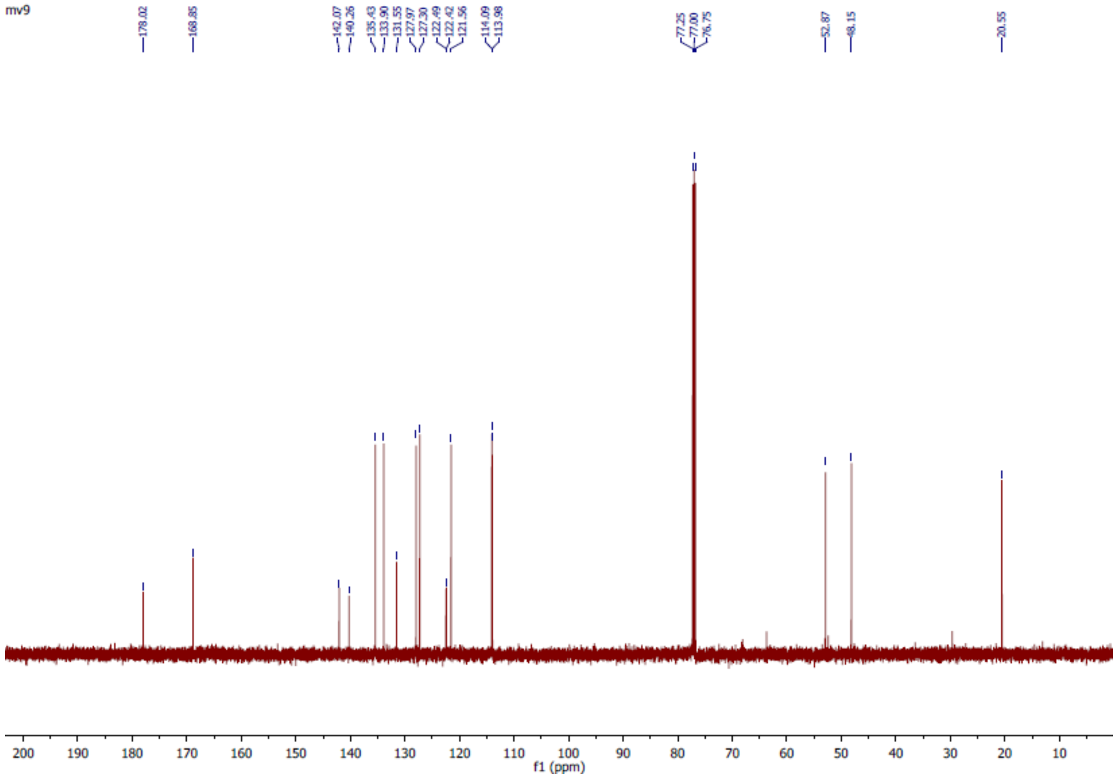
mv9
178.02
168.85
142.07
140.26
135.43
133.90
131.55
127.97
127.30
122.49
122.42
121.56
114.09
113.98
77.25
77.00
76.75
52.87
48.15
20.55
200 190 180 170 160 150 140 130 120 110 100 90 80 70 60 50 40 30 20 10
f1 (ppm)

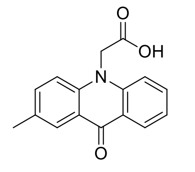
O
OH
N
O

6

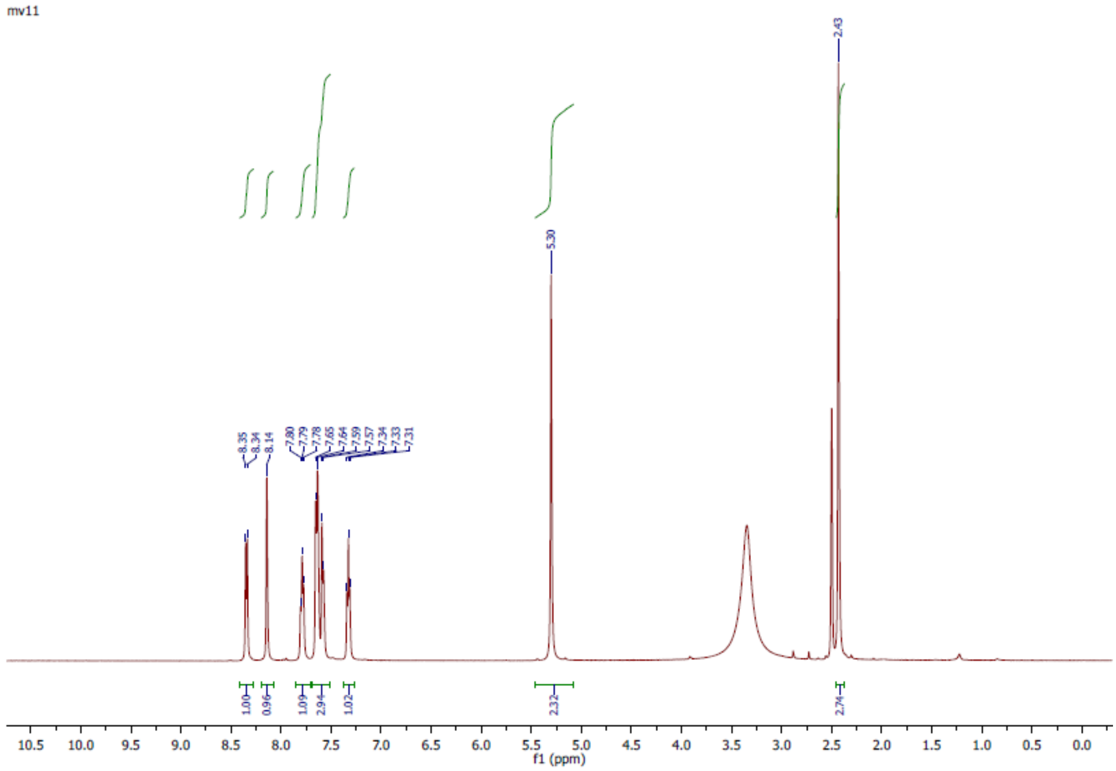
mv11
2.43
5.30
8.35
8.34
8.14
7.80
7.79
7.78
7.65
7.64
7.59
7.57
7.34
7.33
7.31
1.00
0.96
1.09
2.94
1.02
2.32
2.74
f1 (ppm)

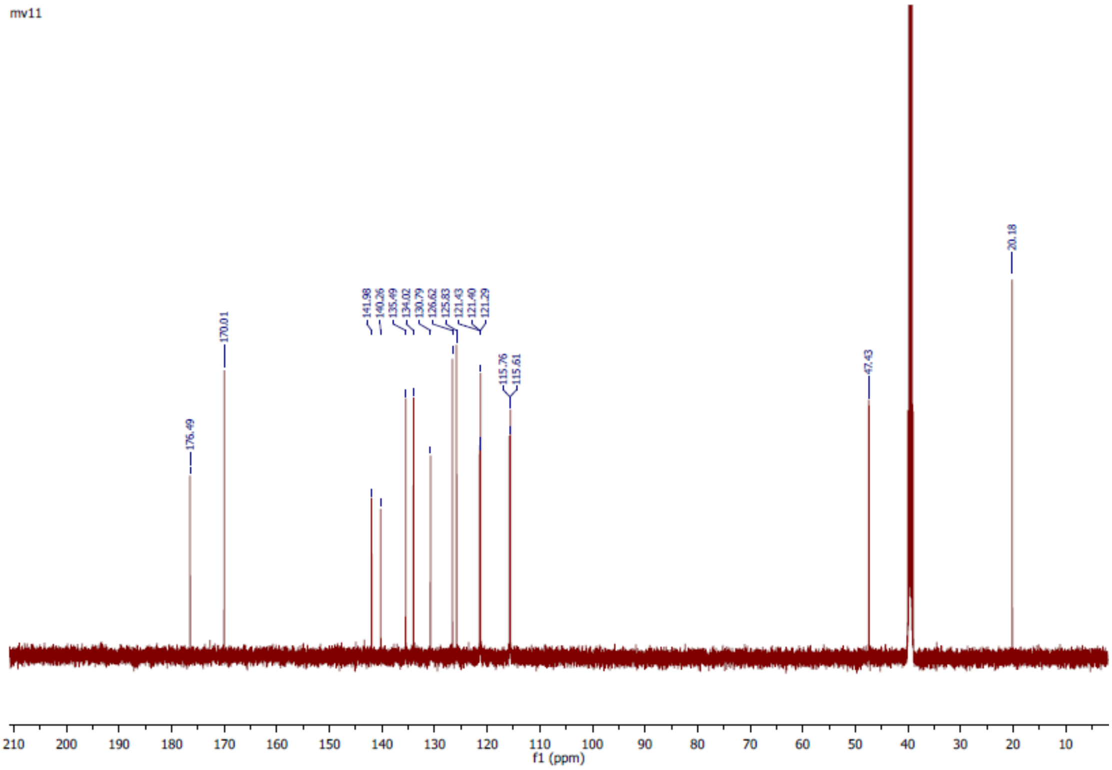
mv11
20.18
141.98
140.26
135.49
134.02
130.79
126.62
125.83
121.43
121.40
121.29
170.01
115.76
115.61
47.43
176.49
f1 (ppm)

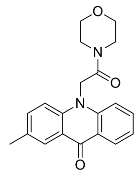

7a

mv12

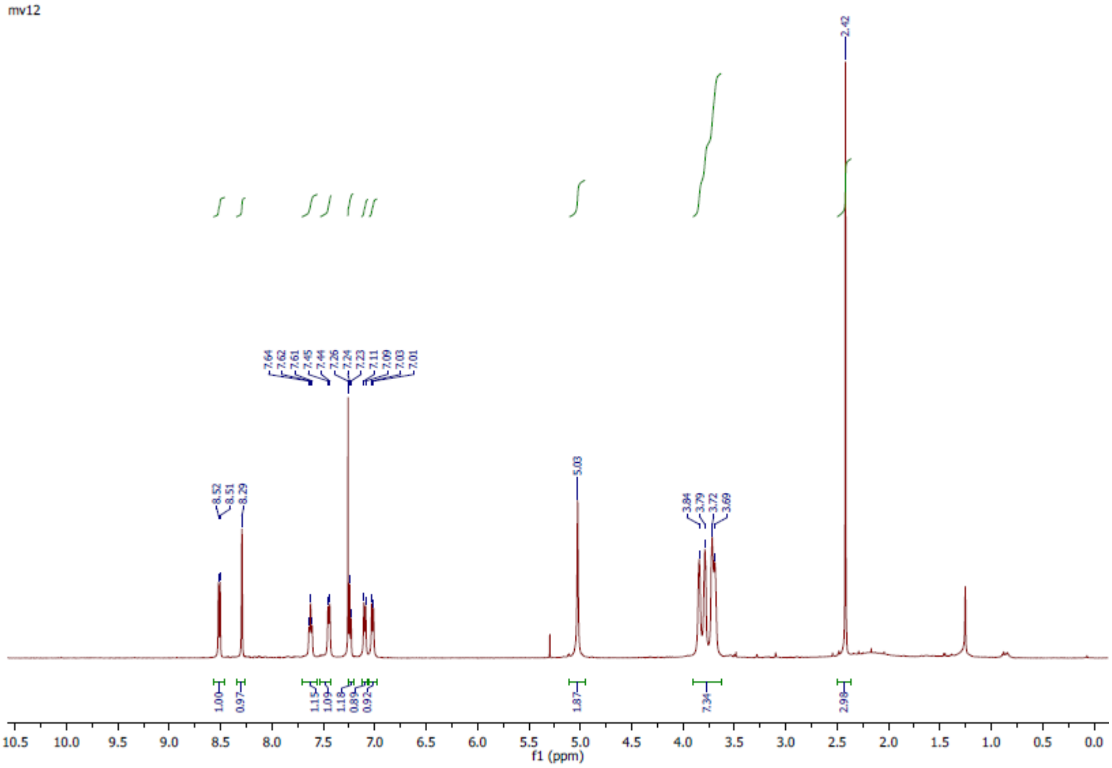

2.42
7.64 7.62 7.61 7.45 7.44 7.26 7.24 7.23 7.11 7.09 7.03 7.01
5.03
8.52 8.51 8.29
3.84 3.79 3.72 3.69
1.00 0.97 1.15 1.09 1.18 0.89 0.92 1.87 7.34 2.98
10.5 10.0 9.5 9.0 8.5 8.0 7.5 7.0 6.5 6.0 5.5 5.0 4.5 4.0 3.5 3.0 2.5 2.0 1.5 1.0 0.5 0.0
f1 (ppm)

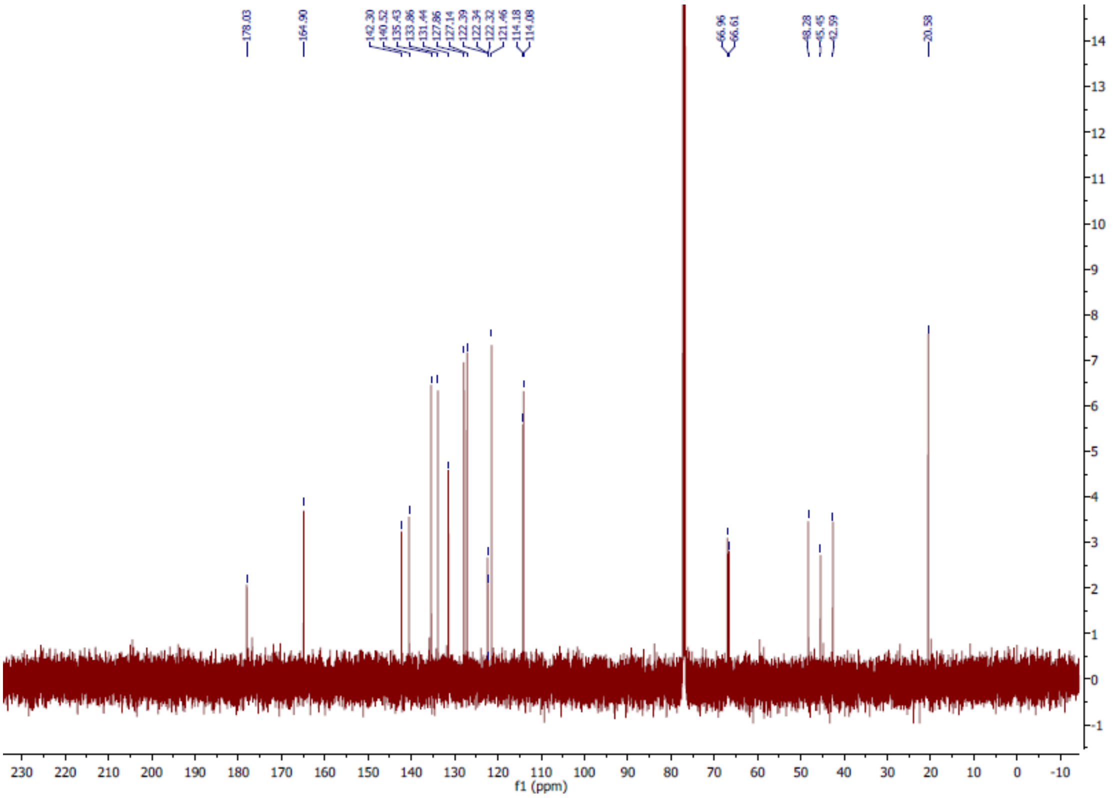

178.03
164.90
142.30 140.52 135.43 133.86 131.44 127.86 127.14 122.39 122.34 122.32 121.46 114.18 114.08
66.96 66.61
48.28 45.45 42.59
20.58
230 220 210 200 190 180 170 160 150 140 130 120 110 100 90 80 70 60 50 40 30 20 10 0 -10
f1 (ppm)

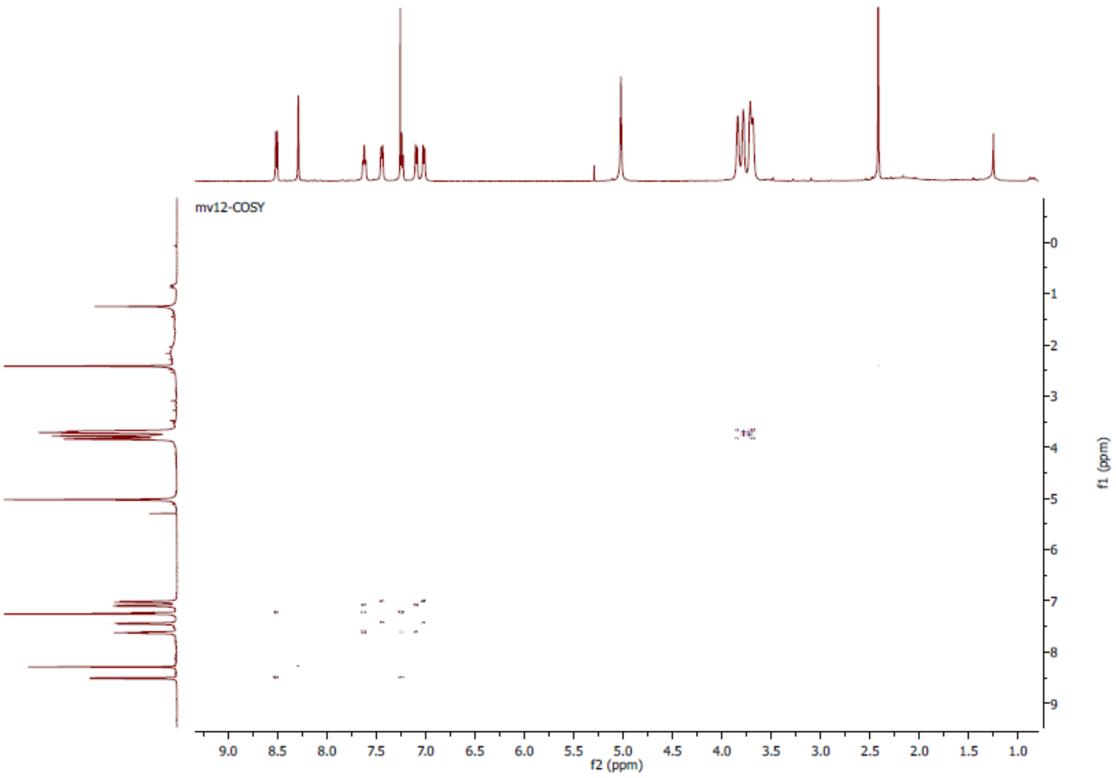

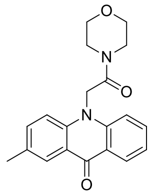

7a

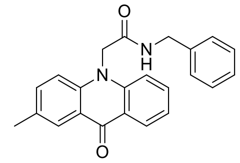
O
N
H
N
O

7b

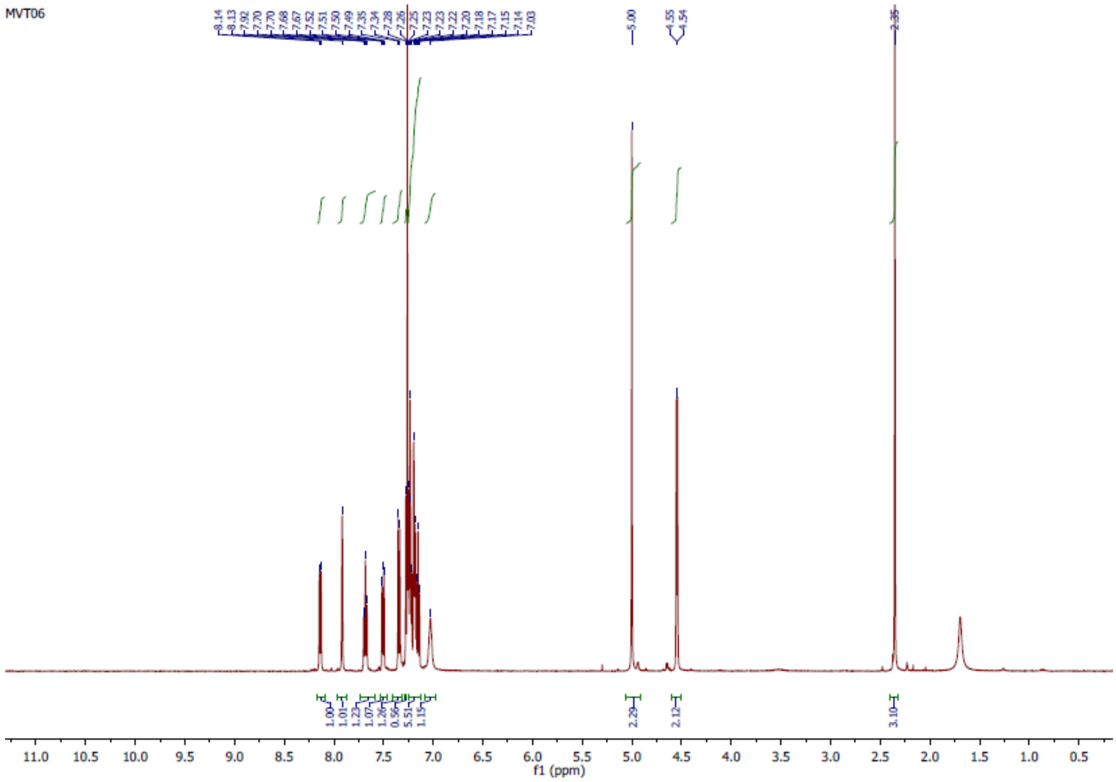
MVT06
f1 (ppm)

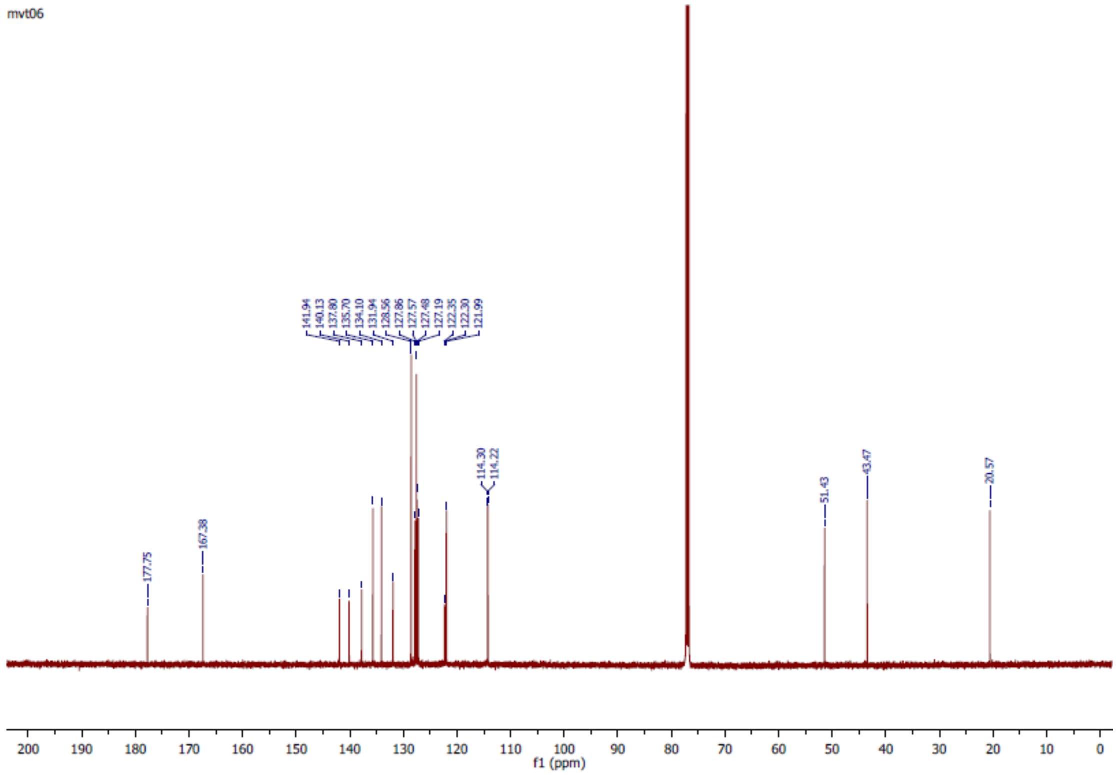
mvt06
177.75
167.38
51.43
43.47
20.57
114.30
114.22
f1 (ppm)

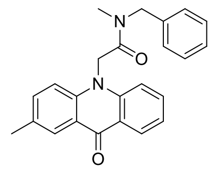

7c

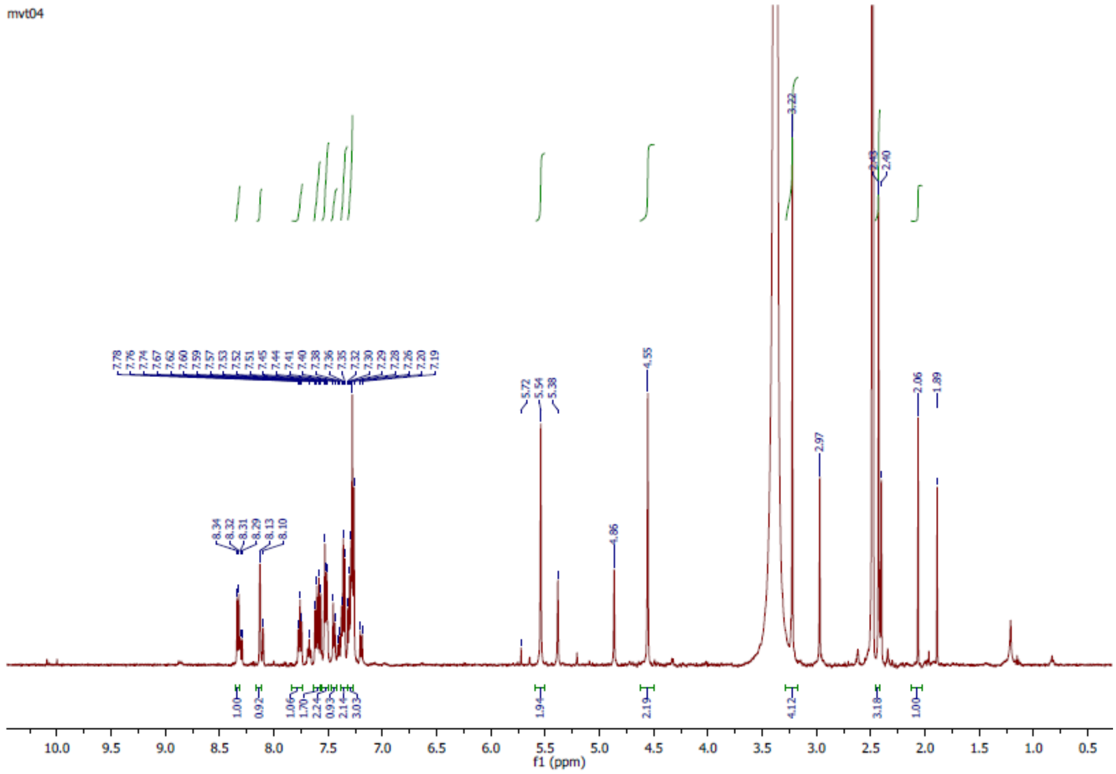
mvt04
7.78
7.76
7.74
7.67
7.62
7.60
7.59
7.57
7.53
7.52
7.51
7.45
7.44
7.41
7.40
7.38
7.36
7.35
7.32
7.30
7.29
7.28
7.26
7.20
7.19
5.72
5.54
5.38
4.55
4.86
8.34
8.32
8.31
8.29
8.13
8.10
3.22
2.97
2.43
2.40
2.06
1.89
1.00
0.92
1.06
1.70
2.24
0.93
2.14
3.03
1.94
2.19
4.12
3.18
1.00
f1 (ppm)

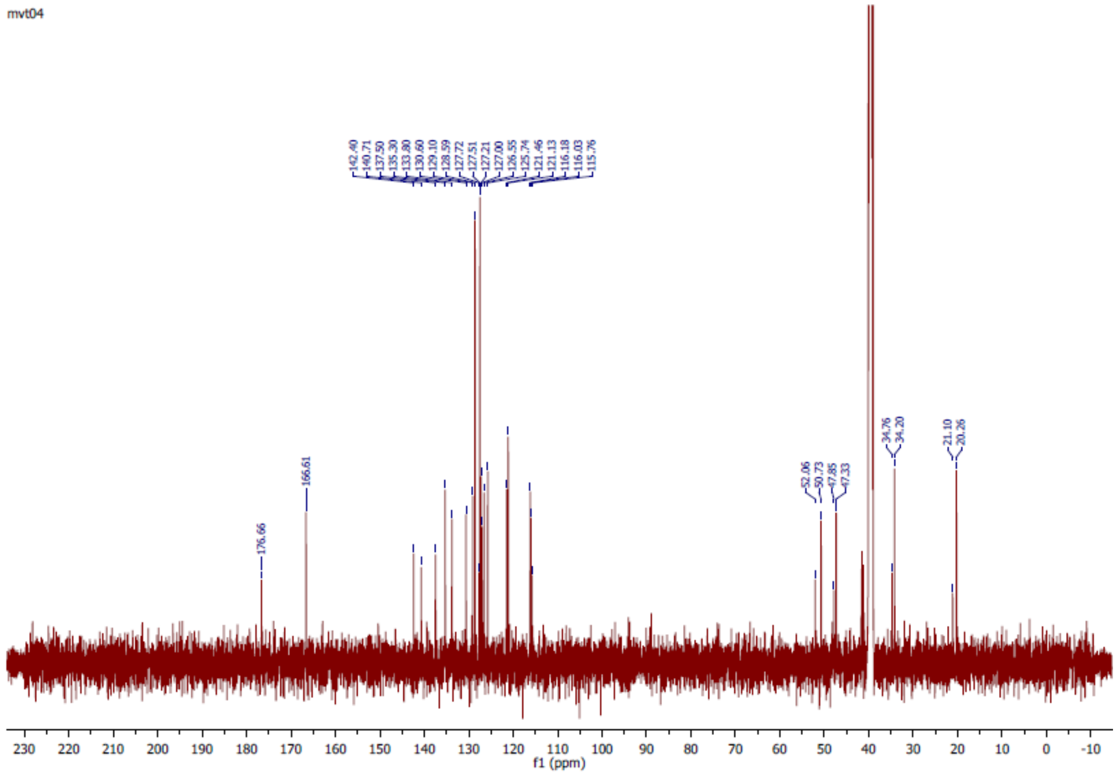
mvt04
142.40
140.71
137.50
135.30
133.80
130.60
129.10
128.59
127.72
127.51
127.21
127.00
126.55
125.74
121.46
121.13
116.18
116.03
115.76
176.66
166.61
52.06
50.73
47.85
47.33
34.76
34.20
21.10
20.26
f1 (ppm)

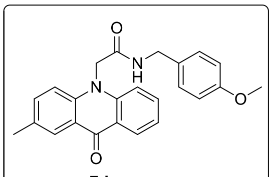

7d

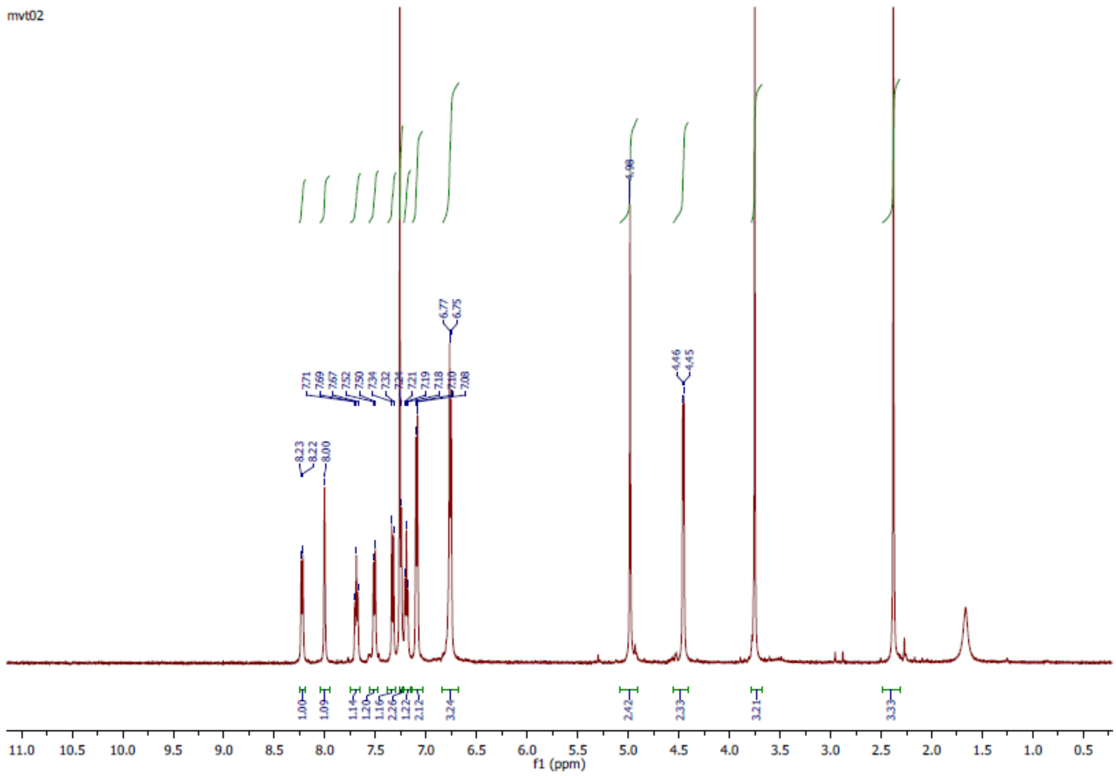

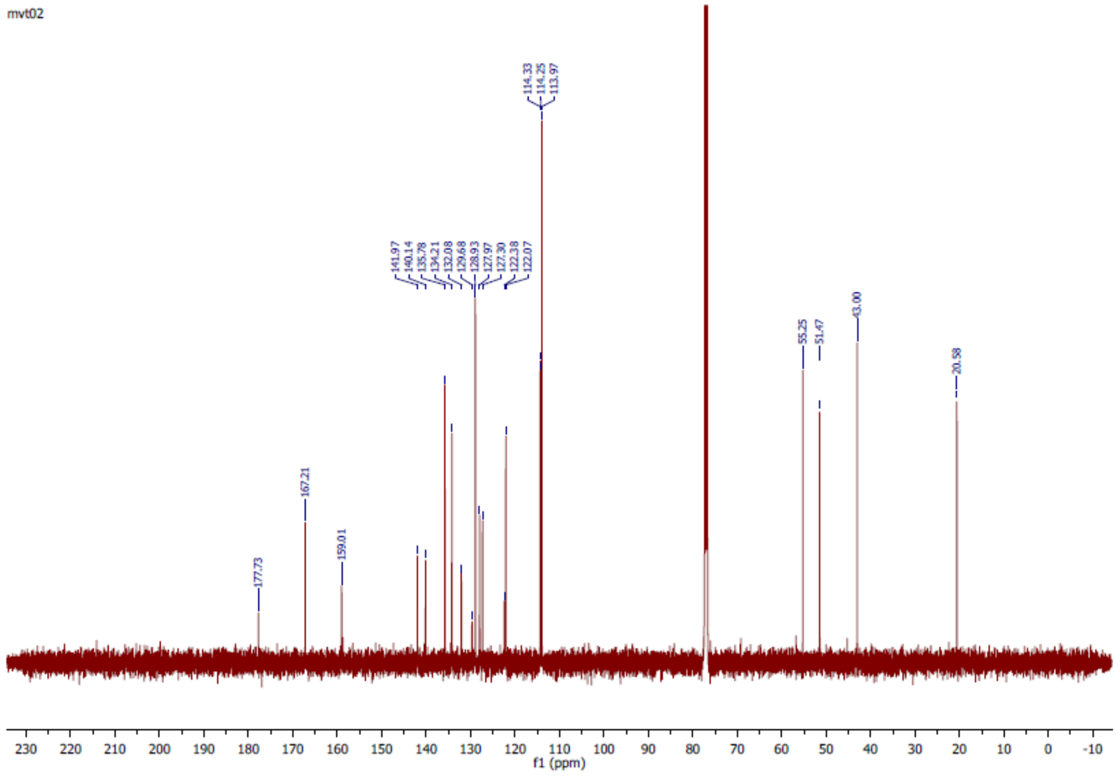

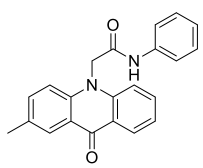

7e

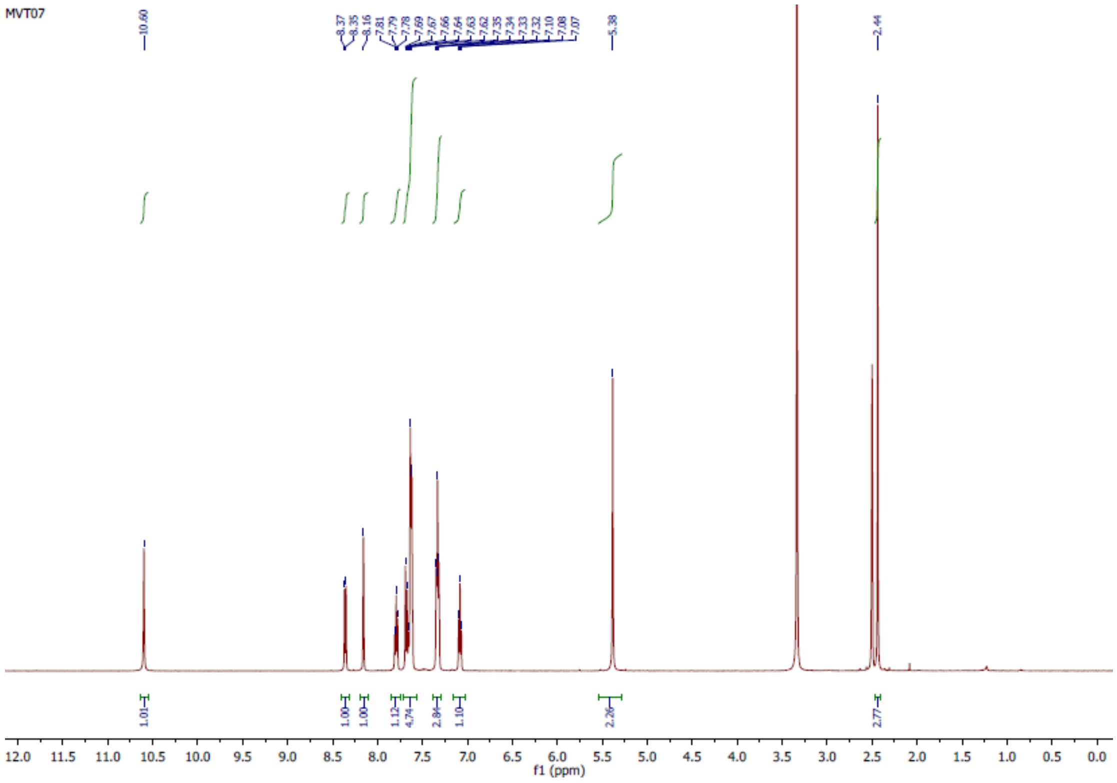
MVT07
10.60
8.37
8.35
8.16
7.81
7.79
7.78
7.69
7.67
7.66
7.64
7.63
7.62
7.35
7.34
7.33
7.32
7.10
7.08
7.07
5.38
2.44
1.01
1.00
1.00
1.12
4.74
2.84
1.10
2.26
2.77
12.0
11.5
11.0
10.5
10.0
9.5
9.0
8.5
8.0
7.5
7.0
6.5
6.0
5.5
5.0
4.5
4.0
3.5
3.0
2.5
2.0
1.5
1.0
0.5
0.0
f1 (ppm)

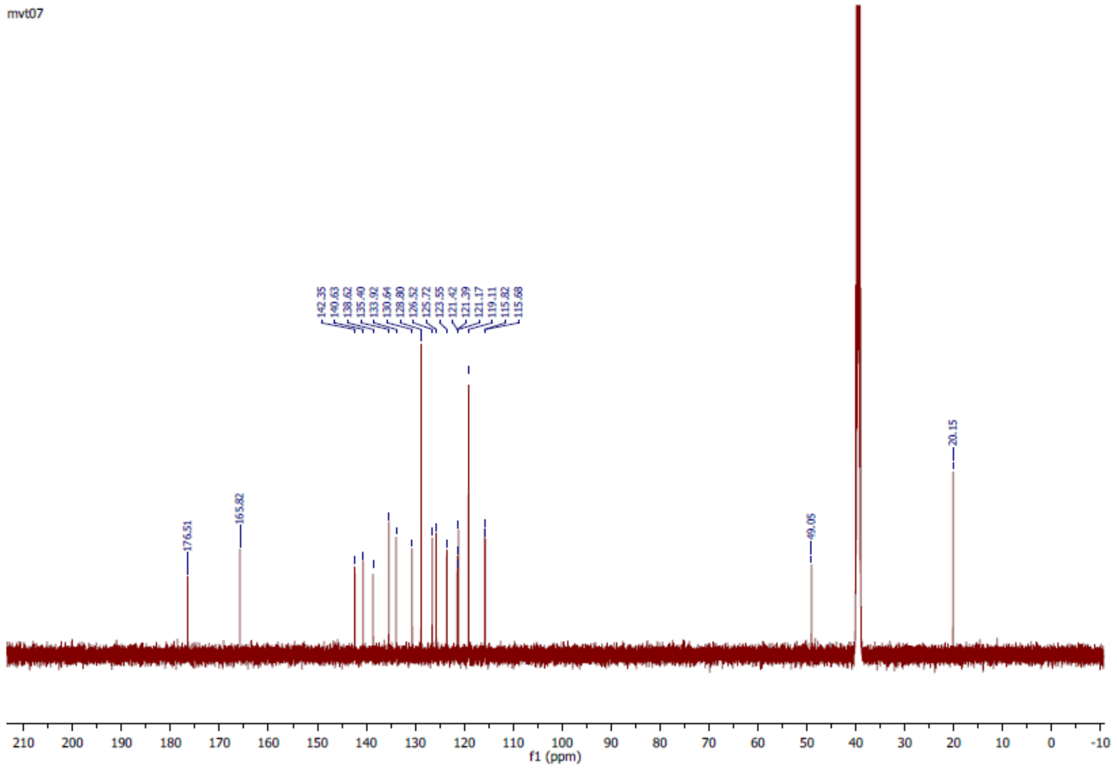
mvt07
176.51
165.82
142.35
140.63
138.62
135.49
133.92
130.64
128.80
126.52
125.72
123.55
121.42
121.39
121.17
119.11
115.82
115.68
49.05
20.15
210
200
190
180
170
160
150
140
130
120
110
100
90
80
70
60
50
40
30
20
10
0
-10
f1 (ppm)

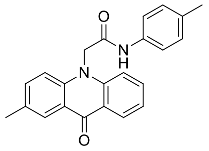

7f

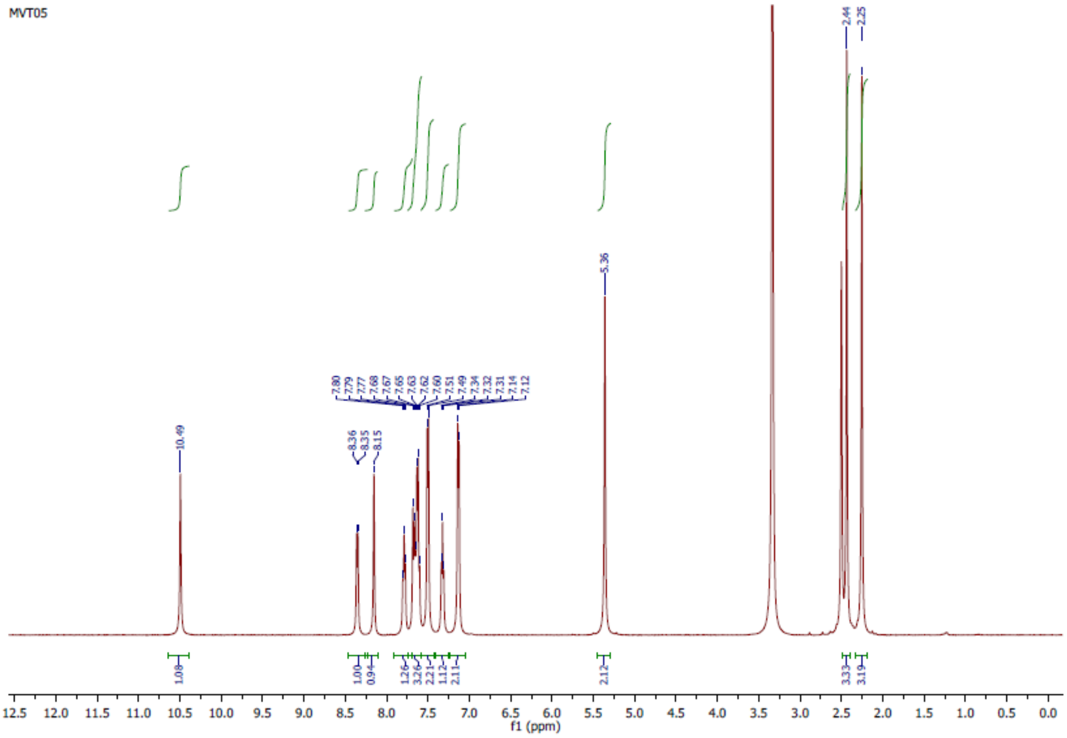
MVT05
10.49
8.36
8.35
8.15
7.80
7.79
7.77
7.68
7.67
7.65
7.63
7.62
7.60
7.51
7.49
7.34
7.32
7.31
7.14
7.12
5.36
2.44
2.25
1.08
1.00
0.94
1.26
3.26
2.21
1.12
2.11
2.12
3.33
3.19
f1 (ppm)

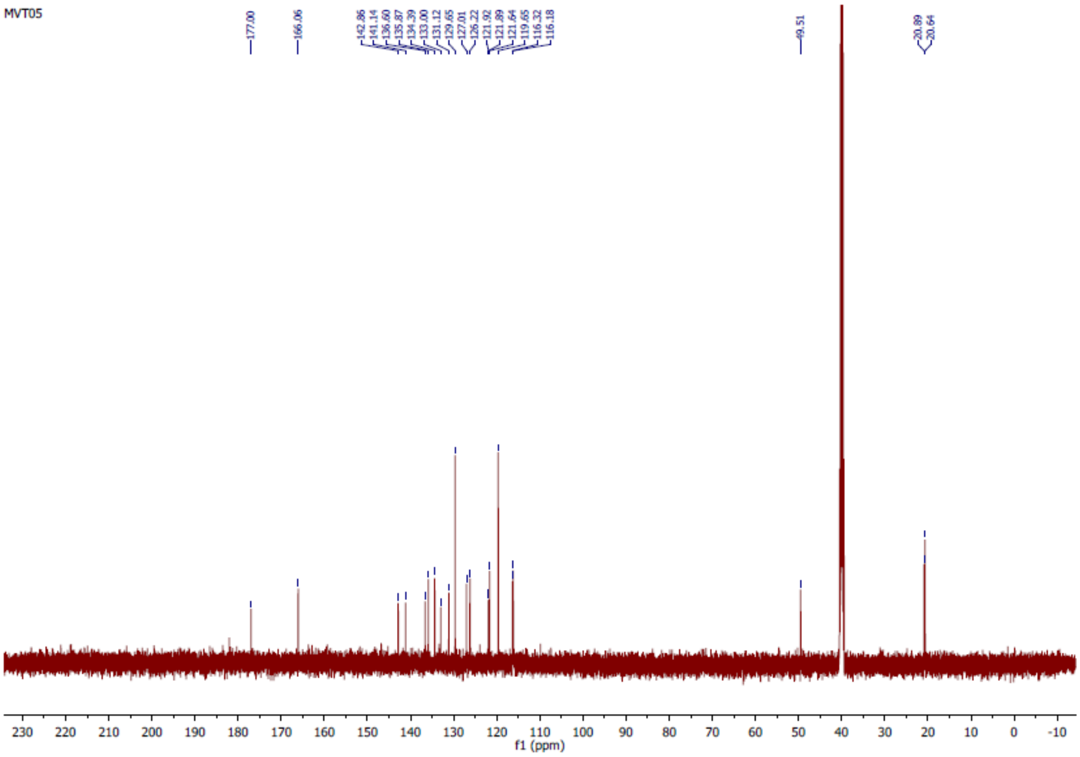
MVT05
177.00
166.06
142.86
141.14
136.60
135.87
134.39
133.00
131.12
129.65
127.01
126.22
121.92
121.89
121.64
119.65
116.32
116.18
49.51
20.89
20.64
f1 (ppm)

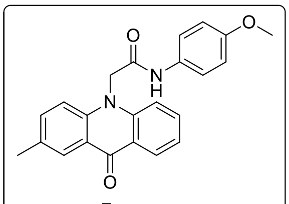

7g

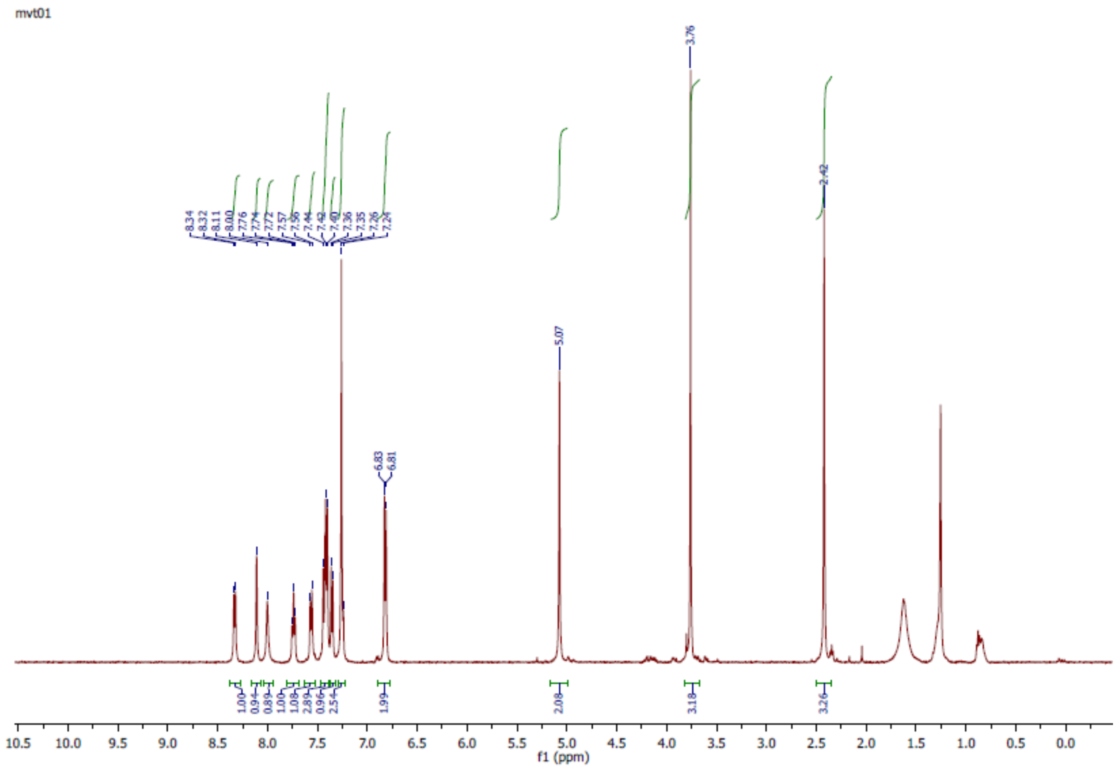
mvt01
3.76
2.42
5.07
6.83
6.81
1.00
0.94
0.89
1.00
1.08
2.89
0.96
2.54
1.99
2.08
3.18
3.26
f1 (ppm)

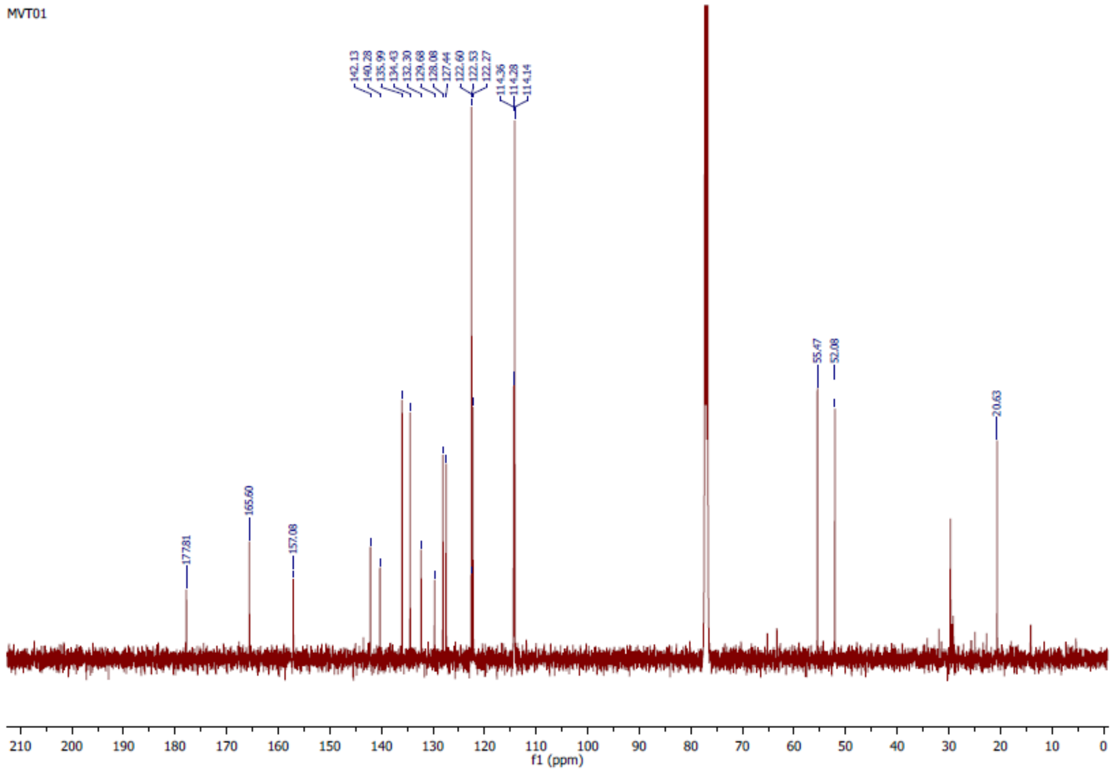
MVT01
142.13
140.28
135.99
134.43
132.30
129.68
128.08
127.44
122.60
122.53
122.27
114.36
114.28
114.14
177.81
165.60
157.08
55.47
52.08
20.63
f1 (ppm)

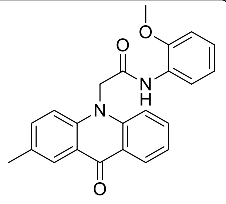

7h

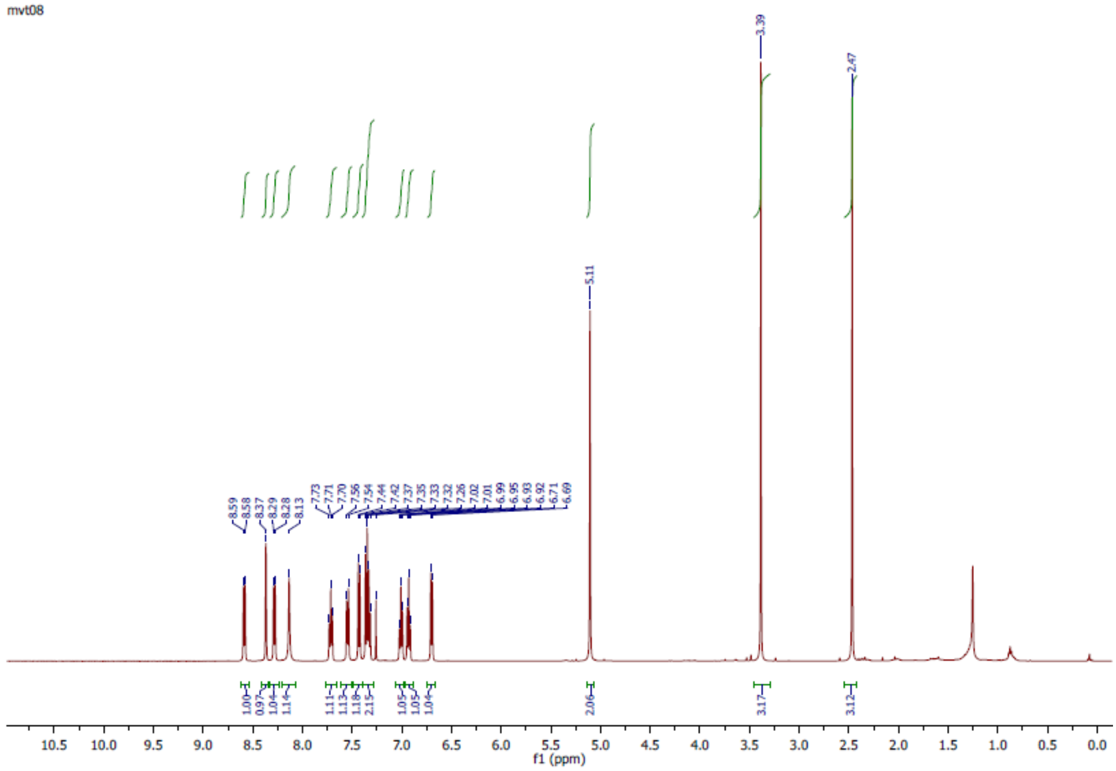

mvt08
8.59
8.58
8.37
8.29
8.28
8.13
7.73
7.71
7.70
7.56
7.54
7.44
7.42
7.37
7.35
7.33
7.32
7.26
7.02
7.01
6.99
6.95
6.93
6.92
6.71
6.69
5.11
3.39
2.47
1.00
0.97
1.04
1.14
1.11
1.13
1.18
2.15
1.05
1.05
1.04
2.06
3.17
3.12
f1 (ppm)

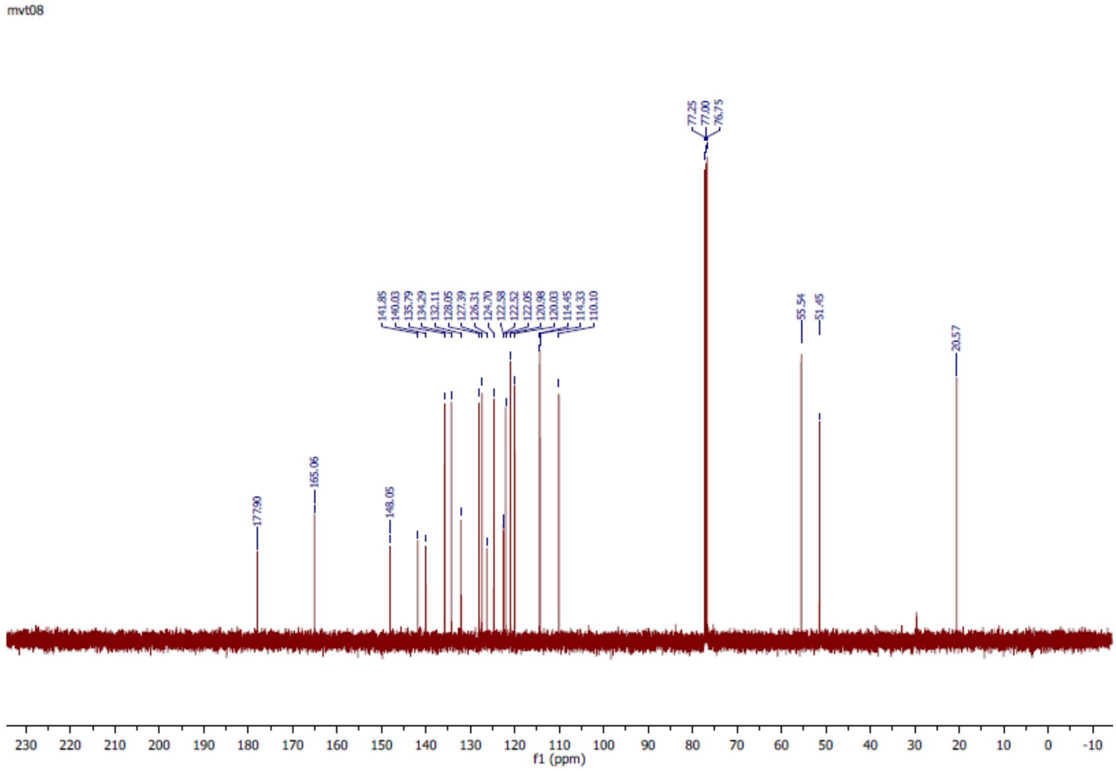

mvt08
177.90
165.06
148.05
141.85
140.03
135.79
134.29
132.11
128.05
127.39
126.31
124.70
122.58
122.52
122.05
120.98
120.03
114.45
114.33
110.10
77.25
77.00
76.75
55.54
51.45
20.57
f1 (ppm)

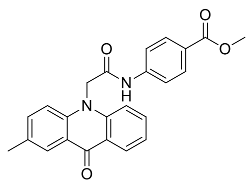

7i

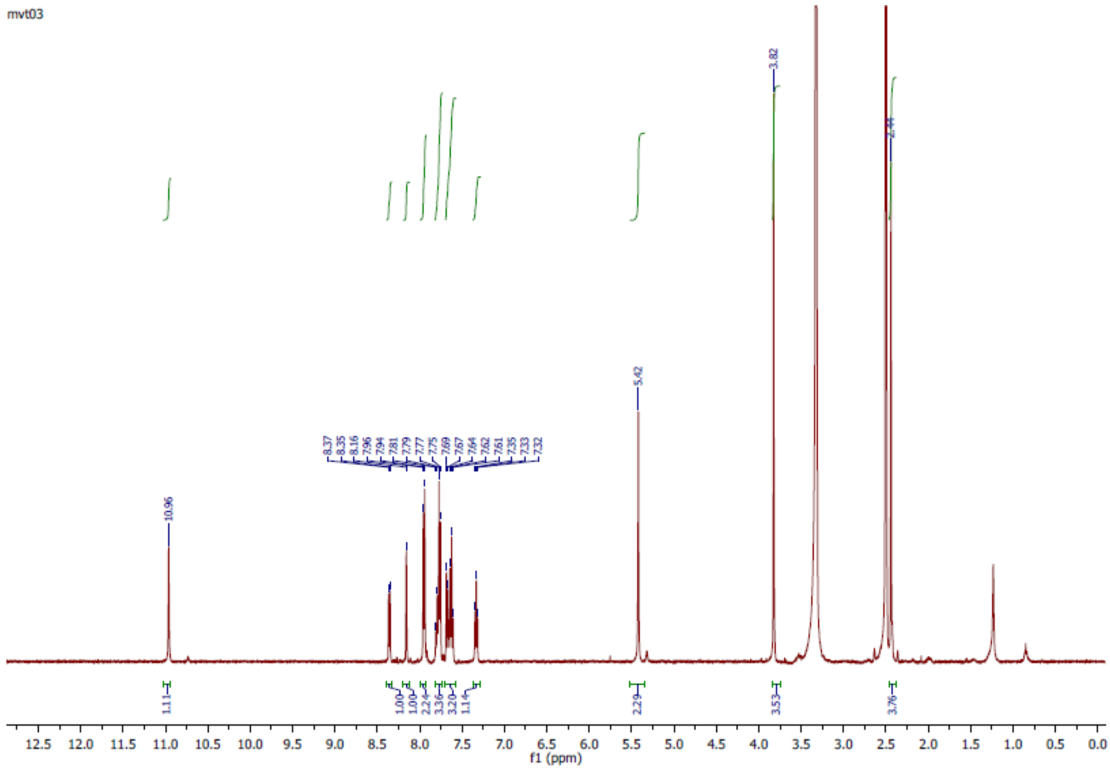

mvt03
10.96
8.37 8.35 8.16 7.96 7.94 7.81 7.79 7.77 7.75 7.69 7.67 7.64 7.62 7.61 7.35 7.33 7.32
5.42
3.82
2.44
1.11
1.00 1.00 2.24 3.36 3.20 1.14
2.29
3.53
3.76
12.5 12.0 11.5 11.0 10.5 10.0 9.5 9.0 8.5 8.0 7.5 7.0 6.5 6.0 5.5 5.0 4.5 4.0 3.5 3.0 2.5 2.0 1.5 1.0 0.5 0.0
f1 (ppm)

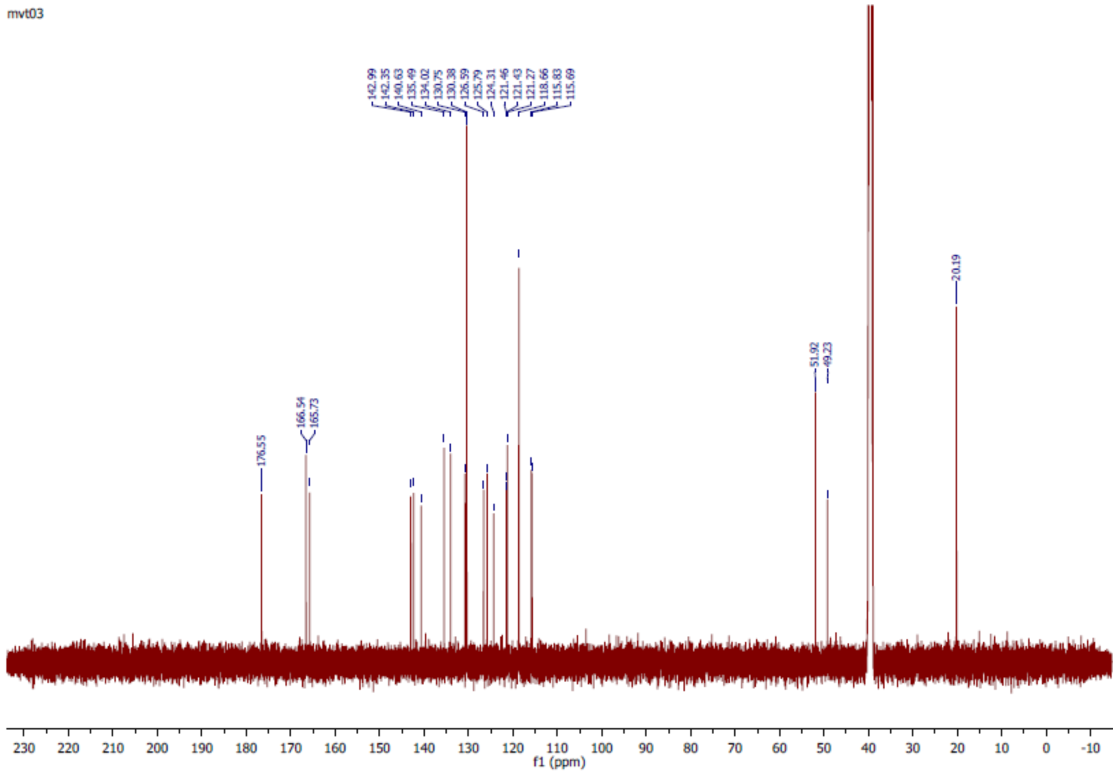

mvt03
176.55
166.54 165.73
142.99 142.35 140.63 135.49 134.02 130.75 130.38 126.59 125.79 124.31 121.46 121.43 121.27 118.66 115.83 115.69
51.92 49.23
20.19
230 220 210 200 190 180 170 160 150 140 130 120 110 100 90 80 70 60 50 40 30 20 10 0 -10
f1 (ppm)

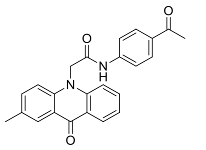
O
O
N
H
N
O

7j

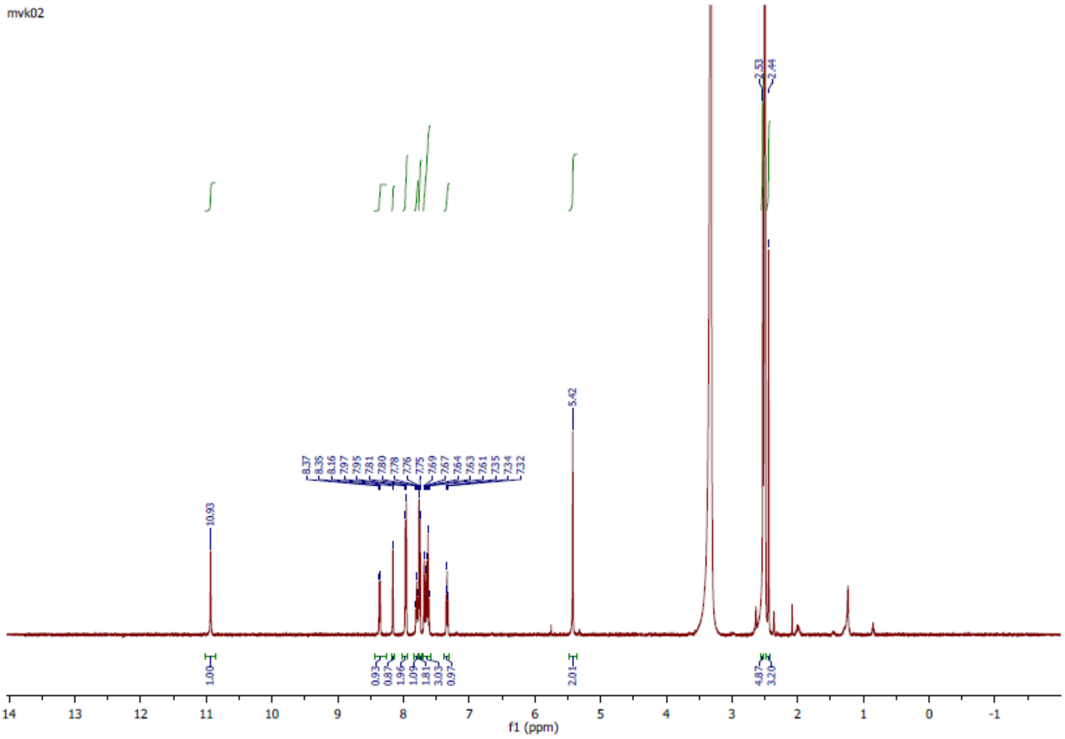
mvk02
10.93
8.37 8.35 8.16 7.97 7.95 7.81 7.80 7.78 7.76 7.75 7.69 7.67 7.64 7.63 7.61 7.35 7.34 7.32
5.42
2.53 2.44
1.00
0.93 0.87 1.96 1.09 1.81 3.03 0.97
2.01
4.87 3.20
f1 (ppm)

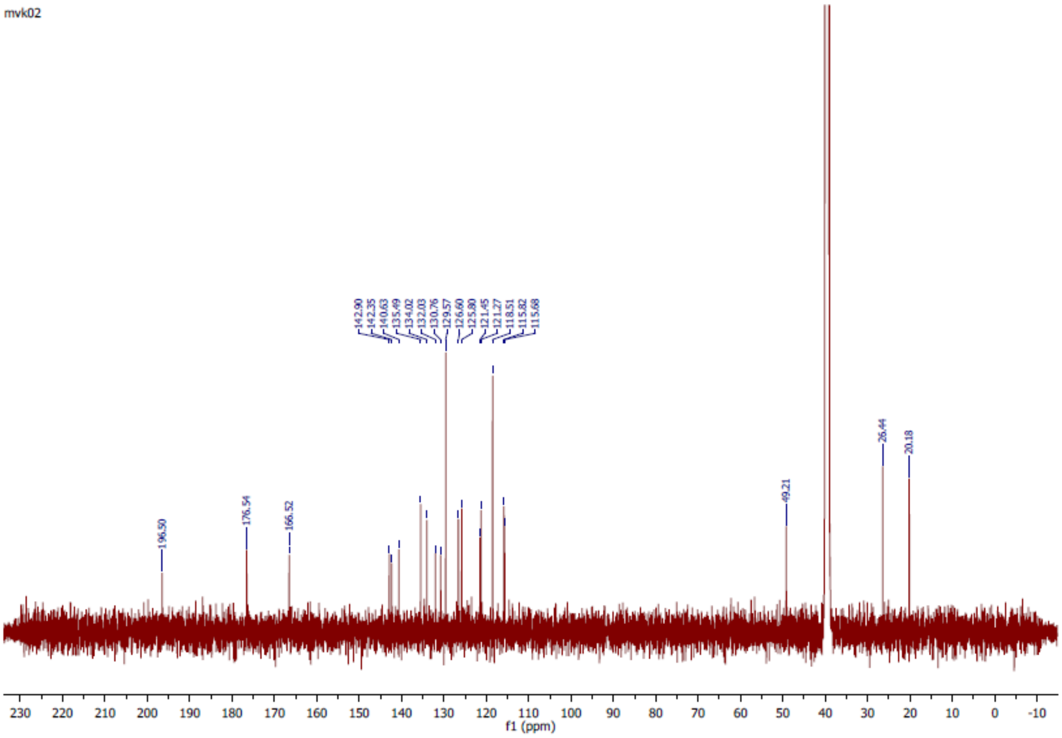
mvk02
196.50
176.54
166.52
142.90 142.35 140.63 135.49 134.02 132.03 130.76 129.57 126.60 125.80 121.45 121.27 118.51 115.82 115.68
49.21
26.44
20.18
f1 (ppm)
